# A tumor profiling resource for ovarian cancer: insights into chemotherapy-driven heterogeneity and personalized treatment strategy

**DOI:** 10.1038/s41467-026-74585-w

**Published:** 2026-07-13

**Authors:** Francis Jacob, Rebekka Wegmann, Joanna Ficek-Pascual, Ulrike Lischetti, Jack Kuipers, Stéphane Chevrier, Michael Prummer, Nora C. Toussaint, Ilaria Alborelli, Ricardo Coelho, Ruben Casanova, Sandra Goetze, Gabriele Gut, Monica-Andreea Baciu-Drăgan, Anne Bertolini, Ximena Bonilla, Jon Brugger, Magdalena M. Brune, Byron Calgua, Stefanie Engler, Cinzia Esposito, Pedro F. Ferreira, Andrea Jacobs, Flavio C. Lombardo, Paola Malsot, Julien Mena, Nicola Miglino, Emanuela S. Milani, Jacobo Sarabia Del Castillo, Sujana Sivapatham, Bettina A. Sobottka-Brillout, Tanmay Tanna, Tibor A. Zwimpfer, The TumorProfiler Consortium, Rebekka Wegmann, Michael Prummer, Ilaria Alborelli, Ruben Casanova, Byron Calgua, Stefanie Engler, Pedro F. Ferreira, Julien Mena, Sujana Sivapatham, Melike Ak, Faisal S. Al-Quaddoomi, Silvana I. Albert, Jonas Albinus, Sonali Andani, Per-Olof Attinger, Monica-Andreea Baciu-Drăgan, Daniel Baumhoer, Beatrice Beck-Schimmer, Lara Bernasconi, Anne Bertolini, Bernd Bodenmiller, Ximena Bonilla, Lars Bosshard, Stéphane Chevrier, Natalia Chicherova, Ricardo Coelho, Maya D’Costa, Esther Danenberg, Natalie R. Davidson, Reinhard Dummer, Martin Erkens, Katja Eschbach, Cinzia Esposito, André Fedier, Joanna Ficek-Pascual, Anja L. Frei, Bruno Frey, Sandra Goetze, Linda Grob, Gabriele Gut, Detlef Günther, Pirmin Haeuptle, Viola Heinzelmann-Schwarz, Sylvia Herter, Rene Holtackers, Tamara Huesser, Alexander Immer, Anja Irmisch, Francis Jacob, Andrea Jacobs, Tim M. Jaeger, Katharina Jahn, Alva R. James, Philip M. Jermann, André Kahles, Abdullah Kahraman, Werner Kuebler, Jack Kuipers, Christian P. Kunze, Christian Kurzeder, Kjong-Van Lehmann, Mitchell Levesque, Ulrike Lischetti, Flavio C. Lombardo, Sebastian Lugert, Gerd Maass, Philipp Markolin, Martin Mehnert, Julian M. Metzler, Nicola Miglino, Emanuela S. Milani, Holger Moch, Riccardo Murri, Charlotte KY Ng, Stefan Nicolet, Marta Nowak, Monica Nunez Lopéz, Patrick GA Pedrioli, Lucas Pelkmans, Salvatore Piscuoglio, Laurie Prélot, Natalie Rimmer, Mathilde Ritter, Christian Rommel, María L. Rosano-González, Gunnar Rätsch, Natascha Santacroce, Jacobo Sarabia del Castillo, Ramona Schlenker, Petra C. Schwalie, Severin Schwan, Tobias Schär, Gabriela Senti, Wenguang Shao, Franziska Singer, Bettina Sobottka, Vipin T. Sreedharan, Stefan Stark, Daniel J. Stekhoven, Tanmay Tanna, Alexandre PA Theocharides, Tinu M. Thomas, Vinko Tosevski, Nora C. Toussaint, Mustafa A. Tuncel, Marina Tusup, Audrey Van Drogen, Marcus Vetter, Tatjana Vlajnic, Sandra Weber, Walter P. Weber, Michael Weller, Fabian Wendt, Norbert Wey, Mattheus HE Wildschut, Bernd Wollscheid, Shuqing Yu, Johanna Ziegler, Marc Zimmermann, Martin Zoche, Gregor Zuend, Rudolf Aebersold, Marina Bacac, Niko Beerenwinkel, Christian Beisel, Viktor H. Koelzer, Simone Muenst, Markus G. Manz, Berend Snijder, Markus Tolnay, Andreas Wicki, Rudolf Aebersold, Marina Bacac, Niko Beerenwinkel, Christian Beisel, Bernd Bodenmiller, Viktor H. Koelzer, Kjong-Van Lehmann, Mitchell P. Levesque, Holger Moch, Simone Muenst, Lucas Pelkmans, Markus G. Manz, Gunnar Rätsch, Franziska Singer, Berend Snijder, Alexandre P. A. Theocharides, Markus Tolnay, Andreas Wicki, Bernd Wollscheid, Viola Heinzelmann-Schwarz

**Affiliations:** 1 Ovarian Cancer Research, Department of Biomedicine, University Hospital Basel and University of Basel, Basel, Switzerland; 2 Institute of Molecular Systems Biology, Department of Biology, ETH Zurich, Zurich, Switzerland; 3 Department of Computer Science, ETH Zurich, Zurich, Switzerland; 4 Department of Biosystems Science and Engineering, ETH Zurich, Basel, Switzerland; 5 Department of Quantitative Biomedicine, University of Zurich, Zurich, Switzerland; 6 SIB Swiss Institute of Bioinformatics, Lausanne, Switzerland; 7 NEXUS Personalized Health Technologies, ETH Zurich, Zurich, Switzerland; 8 Institute of Medical Genetics and Pathology, University Hospital Basel, Basel, Switzerland; 9 Department of Molecular Health Sciences, ETH Zurich, Zurich, Switzerland; 10 Swiss Multi-Omics Center, PHRT-CPAC, ETH Zurich, Zurich, Switzerland; 11 Department of Molecular Life Sciences, University of Zurich, Zurich, Switzerland; 12 AI Center, ETH Zurich, Zurich, Switzerland; 13 Department of Medical Oncology and Hematology, University Hospital Zurich, Zurich, Switzerland; 14 Department of Pathology and Molecular Pathology, University Hospital Zurich, Zurich, Switzerland; 15 University of Zurich, Faculty of Medicine, Zurich, Switzerland; 16 Roche Pharmaceutical Research and Early Development, Roche Innovation Center Zurich, Zurich, Switzerland; 17 Department of Dermatology, University of Zurich Hospital, Zurich, Switzerland; 18 Department of Gynecology and Gynecologic Oncology, University Hospital Basel, Basel, Switzerland; 19 ETH Zurich, Department of Health Sciences and Technology, Zurich, Switzerland; 20 ETH Zurich, Department of Computer Science, Institute of Machine Learning, Zurich, Switzerland; 21 University Hospital Zurich, Biomedical Informatics, Zurich, Switzerland; 22 F. Hoffmann-La Roche Ltd, Basel, Switzerland; 23 University of Zurich, VP Medicine, Zurich, Switzerland; 24 University Hospital Zurich, Clinical Trials Center, Zurich, Switzerland; 25 ETH Zurich, Institute of Molecular Health Sciences, Zurich, Switzerland; 26 University Hospital Basel and University of Basel, Department of Biomedicine, Basel, Switzerland; 27 University of Zurich, Institute of Molecular Life Sciences, Zurich, Switzerland; 28 Life Science Zurich Graduate School, Biomedicine PhD Program, Zurich, Switzerland; 29 Roche Diagnostics GmbH, Penzberg, Germany; 30 ETH Zurich, Department of Chemistry and Applied Biosciences, Zurich, Switzerland; 31 Cantonal Hospital Baselland, Medical University Clinic, Liestal, Switzerland; 32 University Hospital Basel, Gynecological Cancer Center, Basel, Switzerland; 33 Max Planck ETH Center for Learning Systems, Zurich, Switzerland; 34 FHNW, School of Life Sciences, Institute of Chemistry and Bioanalytics, Muttenz, Switzerland; 35 University Hospital Basel, Spitalstrasse 21/Petersgraben 4, Basel, Switzerland; 36 University Hospital Basel, Department of Information- and Communication Technology, Basel, Switzerland; 37 University Hospital Basel, Brustzentrum, Basel, Switzerland; 38 Cancer Research Center Cologne-Essen, University Hospital Cologne, Cologne, Germany; 39 Center for Integrated Oncology Aachen (CIO-A), Aachen, Germany; 40 Joint Research Center Computational Biomedicine, University Hospital RWTH Aachen, Aachen, Germany; 41 University Hospital Zurich, Department of Gynecology, Zurich, Switzerland; 42 University of Zurich, Services and Support for Science IT, Zurich, Switzerland; 43 University of Bern, Department of BioMedical Research, Bern, Switzerland; 44 ETH Zurich, Department of Biology, Zurich, Switzerland; 45 Roche Pharmaceutical Research and Early Development, Roche Innovation Center Munich, Roche Diagnostics GmbH, Penzberg, Germany; 46 ETH Zurich, Swiss Data Science Center, Zurich, Switzerland; 47 University Hospital Basel, Brustzentrum & Tumorzentrum, Basel, Switzerland; 48 University Hospital Basel and University of Basel, Department of Surgery, Brustzentrum, Basel, Switzerland; 49 University Hospital and University of Zurich, Department of Neurology, Zurich, Switzerland; 50 University Hospital Zurich, Zurich, Switzerland; 51 Present Address: Navignostics AG, Horgen, Switzerland; 52 Present Address: Swiss Data Science Center, ETH Zürich, Zurich, Switzerland

**Keywords:** Ovarian cancer, Cancer screening

## Abstract

In women with high-grade serous ovarian cancer, chemotherapy remains the primary standard treatment, despite growing recognition of the disease as highly heterogeneous. Here, we examine the feasibility and clinical utility of comprehensive multimodal molecular profiling to inform treatment decisions. We analyze blood, single-cell and bulk tumor tissue, and malignant ascites using up to eleven technologies (DNA, RNA, protein, and functional assays) within a four-week turnaround time. Hypothetical treatment recommendations are altered for 76% of patients, and multi-omics-guided maintenance therapy is associated with prolonged overall survival in a subset of patients. Subsequent cohort analysis reveals distinct cellular and molecular profiles in ascites-derived single-cells compared to solid tumor tissue, unique per-patient ex vivo drug responses, and a marked increase in cancer cell heterogeneity following chemotherapy exposure. This coincides with genomic signature alterations in whole-genome-amplified patients. Our data suggest that molecularly guided treatments should be tested as adjuvant therapies prior to chemotherapy in the future.

## Abstract (web summary)

High-grade serous ovarian cancer is clinically challenging due to marked molecular heterogeneity and variable treatment response. Here, the authors demonstrate that integrated multimodal tumor profiling can inform personalized maintenance treatment decisions and that chemotherapy reshapes tumor cell diversity.

## Introduction

Based on molecular tumor profiling, cancer treatment decisions are modified from a general towards a personalized approach, mostly based on genomic biomarkers^1^. To identify effective personalized therapies, clinical trials- such as the BOUQUET trial (NCT04931342) – are focussing on the efficacy of predictive marker-based interventions. Although targeted therapies have shown promise, several studies reported only marginal benefit when comparing genomics-guided treatment with physicians’ choice^2–4^. This is often observed in heterogeneous patient populations, where the absence of targetable mutations and predictive biomarkers limits the ability to stratify patients for tailored treatments. Moreover, most personalized clinical trials investigate a heavily pretreated and thus heterogeneous patient cohort. Therefore, studies focusing on earlier treatment phases and applying more sophisticated matching schemes showed improved treatment outcomes with genomics-guided precision medicine^5–7^. The inclusion of transcriptomic measurements alongside genomics seems to further enhance the effectiveness of drug-to-patient matching^8–10^. These findings highlight the need for research that investigates the utility of integrative, multi-omics profiling in earlier treatment lines.

High-grade serous ovarian cancer (HGSOC) presents significant challenges due to its poor prognosis^11^ and high heterogeneity, which complicates treatment responses. *BRCA* mutations and homologous-repair deficiency (HRD) have emerged as predictive biomarkers for targeted therapies with PARP inhibitors (PARPi) in the maintenance phase^12–18^. PARPi have significantly improved recurrence-free and overall survival in these patients. However, a substantial number of patients, most of whom are HR-proficient (HRP), do not benefit from PARPi and also are carboplatin chemotherapy resistant. Both HRD and HRP are characterized by complex heterogeneity, significantly influencing treatment responses and contributing to inherent and acquired drug resistance^19–21^. Consequently, patients without response to PARPi experience a low 5-year survival rate of 30–40%^22,23^. In addition, HGSOC shows a high genomic instability and mutations in various tumor suppressor genes like *TP53, NF1*, *RB1*, and *PTEN*^24^. Tumor cells also exhibit *MYC*-driven proliferation, dysregulated cell cycle checkpoints and copy number alterations (CNA)^25^. The interplay between cancer cells and the tumor microenvironment (TME) further complicates disease evolution and therapeutic response^26^. Given the complex genetic landscape and TME interactions, innovative strategies are essential for enhancing precision oncology approaches to improve outcomes in HGSOC^27^.

In this prospective, multi-center, exploratory gynecological Swiss Tumor Profiler (TuPro) research project (NCT06599749), we implement comprehensive clinical, experimental and computational workflows to identify molecular signatures and predict effective treatments for patients. This was performed within a clinically useful timeframe of one month^28^. Our goal is to assess the biological insight gained from multi-omics guidance compared to clinical routine and standard genomic analysis. By analyzing tumor samples from patients at different treatment stages, spanning from chemo-naïve to relapsed status, we aim to uncover insights that could inform future clinical trials and enhance the efficacy of personalized treatment approaches. The resulting unique dataset captures the molecular complexity of HGSOC before and after chemotherapy, revealing that chemotherapy alters cancer cell populations, coinciding with increased whole-genome amplification and greater patient divergence toward disease recurrence.

## Results

### Tumor Profiler analyses enable multi-modal profiling of HGSOC in a clinically useful workflow

**Fig. 1:**
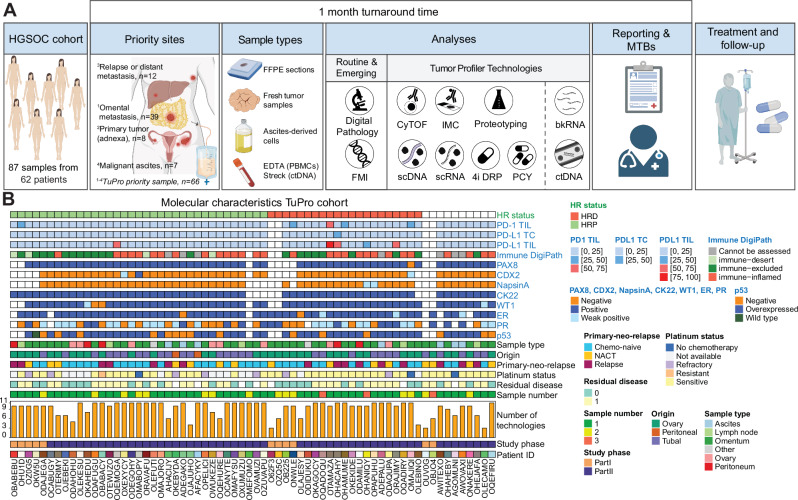
Ovarian cancer Tumor Profiler Cohort: Clinical cohort overview and molecular assessment using routine and emerging technologies. **A** Overview of the Swiss Tumor Profiler (TuPro) study design with cohort structure and applied technologies. Because some patients contributed multiple specimens from the same time point, a single priority sample was defined for each patient according to a pre-specified hierarchy: (1) omental metastasis, (2) other tissue (distant metastasis or primary tumor), and (3) ascites. **B** Routine and emerging diagnostic features of HGSOC assessed by immunohistochemistry (blue row annotation) and targeted next-generation sequencing (green row annotation). White squares indicate missing data. Routine clinical and pathological characteristics are ordered by homologous recombination (HR) status (defined by pathogenic *BRCA1/BRCA2* variants and loss-of-heterozygosity (LOH) determined by FMI), followed by study phase (Part I: proof-of-concept and platform establishment; Part II: profiling phase for MTB recommendations). TuPro technology coverage (Number of technologies) for each sample is shown as a bar chart. See also Supplementary Figs. 1–3, Supplementary Table 1, 2, Supplementary Data 1–11. Source data are provided as a Source Data file.

In this TuPro trial, tumor tissue and ascites samples, along with longitudinal blood samples from chemotherapy-naive (chemo-naive), neoadjuvant chemotherapy (NACT)-treated, and recurrent HGSOC patients, were prospectively profiled. A total of 87 samples were obtained from 62 patients, resulting in the collection of 66 samples from pre-determined “priority sites” (Fig. 1A and Supplementary Data 1). Several state-of-the-art omics technologies and functional readouts were applied to support hypothetical clinical decision-making. Treatment suggestions provided by “explorative technologies” were compared to “emerging diagnostics” and routine clinical care (Fig. 1A and Supplementary Table 1). The routine and emerging diagnostics arm included digital pathology^29^ and next-generation sequencing (NGS) by FoundationOne^Ⓡ^CDx (FMI, Supplementary Data 2), while the exploratory arm encompassed bulk RNA-sequencing (bkRNA-seq, Supplementary Data 3), proteotyping (Supplementary Data 4)^30–32^, circulating-tumor DNA-seq (ctDNA-seq, Supplementary Data 5)^33–35^, single-cell RNA-seq (scRNA-seq, Supplementary Data 6)^36–38^, scDNA-seq for copy number (CN) assessment (Supplementary Data 7)^39^, mass cytometry (CyTOF, Supplementary Data 8)^40^, spatial imaging mass cytometry (IMC, Supplementary Data 9) on formalin-fixed, paraffin-embedded (FFPE) samples^41^, and ex vivo drug response testing (Pharmacoscopy/PCY, Supplementary Data 10)^42–46^ and iterative indirect immunofluorescence imaging drug response profiling (4i DRP, Supplementary Data 11)^47^. These technologies encompass DNA, RNA and protein analyses, providing insights into molecular biology at various levels of resolution, while also integrating measurements of drug response effects. Solid tumor tissue, ascites and blood samples were obtained from three Swiss hospitals (Supplementary Table 2). Samples were processed and stored in a central laboratory, which also applied quality control measures prior to distribution of samples to individual TuPro laboratories specializing in the various technologies. These laboratory expert groups are referred to as “TuPro nodes” (Supplementary Fig. 1). Multiple samples were collected from each patient at each time point, and samples were prioritized based on tissue type (with omental metastasis given the highest priority, followed by other solid tumor tissues). Technologies were also prioritized; therefore, due to limitations in cell numbers and technology-dependent quality criteria, not all samples were analyzed using every platform (Supplementary Table 1). Given the priority lists and technology-specific quality requirements, 29 (43.9%) and 49 (74.2%) of the total 66 priority HGSOC tissue samples were analyzed by all 11 or at least 9 technologies, respectively (Fig. 1B and Supplementary Fig. 2). Corresponding blood-derived samples were also analyzed using CyTOF and the plasma fraction was used for ctDNA-seq. A tumor-matching FFPE-derived sample per patient was assessed digitally by routine pathology (Fig. 1B and Supplementary Table 1). This evaluation revealed that the majority of priority samples arose from patients carrying an immune-excluded or -inflamed TME. This predominance reflects the characteristic immune landscape of advanced-stage HGSOC, which frequently presents with omental and peritoneal metastases. As the majority of samples were derived from metastatic sites in advanced stage chemo-naïve patients with HGSOC, this likely preserved the intrinsic immune composition and minimized treatment-related alterations. This distinction is important, as previous studies have primarily analyzed primary ovarian tumors^48,49^, although metastatic lesions in HGSOC are known to differ in their immune contexture^50–52^. The TuPro patient samples also showed a trend towards low PD-1 and PD-L1 expression. Immunohistochemistry (IHC) performed on matched routine pathology specimens confirmed an expected overall high abundance of PAX8, CK22, WT1, ER, and p53 protein, together with maintained ARID1A expression (Fig. 1B and Supplementary Fig. 3). As reported previously for HGSOC, the high proportion of p53-aberrant cases was also observed through routine IHC. All samples were microsatellite stable.

### Multi-omics tumor profiling alters treatment recommendations

The TuPro data were aggregated and summarized within a four-week turnaround time and subsequently presented to a dedicated molecular tumor board (MTB). Each MTB involved a multidisciplinary team of approximately 16 participants, including gynecologic and medical oncologists, molecular pathologists, and scientists representing each TuPro technology platform. To ensure clinical relevance and transparency, every MTB followed a standardized three-step workflow.

**Fig. 2:**
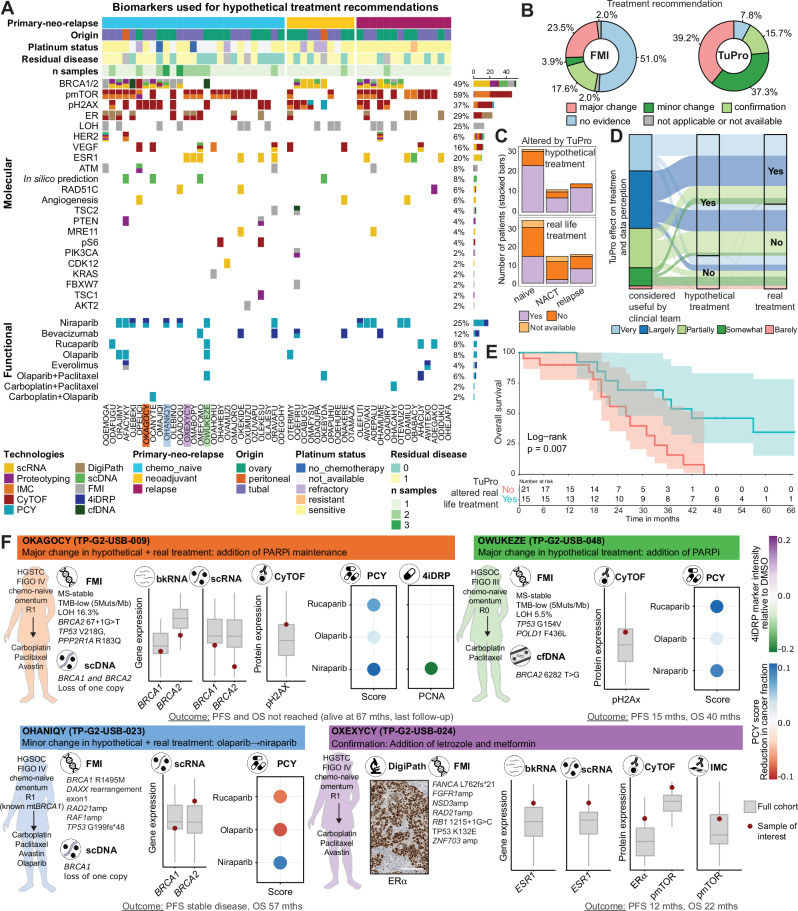
Biomarker-driven treatment recommendations and the impact of TuPro data on hypothetical and real clinical decision-making. **A** Overview of biomarkers and technologies contributing to hypothetical treatment recommendations. Oncoprint summarizes biomarker findings stratified by clinical setting (chemo-naive, neoadjuvant chemotherapy (NACT), and relapse), with contributing technologies color-coded. The proportion of patients for whom each biomarker informed a treatment recommendation is shown as a bar chart on the right. **B** Pie charts comparing MTB treatment recommendations based on routine clinical assessment, FMI-derived targeted genomic profiling, and TuPro multi-omics data. **C** Stratification of hypothetical (left) and real (right) TuPro-altered treatment recommendations across chemo-naive, neoadjuvant (NACT), and relapse patients, shown as stacked bar charts (total number of time points indicated). **D** Alluvial plot illustrating the perceived usefulness of each MTB, as rated by participating clinicians, further split into hypothetical and real treatment recommendations. **E** Kaplan-Meier analysis of overall survival for all newly diagnosed patients (chemo-naive and post-NACT patients). Shaded regions represent 95% confidence intervals; *p*-value from log-rank test. **F** Selected patient cases illustrating FMI- and TuPro-informed treatment modifications. Representative examples highlight clinical details, molecular findings, and multi-omic evidence (FMI, cfDNA, scDNA, scRNA, CyTOF, PCY, 4iDRP, DigiPath, IMC) supporting hypothetical and real treatment recommendations, including changes in PARP inhibitor maintenance. Box plots show the median (horizontal line), interquartile range (box), and whiskers extending to 1.5 x the interquartile range. Human female outline adapted from NIAID Visual & Medical Arts, NIAID NIH BioArt Source (bioart.niaid.nih.gov/bioart/221). See also Supplementary Fig. 4 and Supplementary Data 12. Source data are provided as a Source Data file.

First, the treating physicians presented the clinical case, including disease history and, if applicable, prior therapies, and outlined a treatment plan based on current clinical guidelines (e.g., ESGO/ESMO consensus recommendations). Second, targeted genomic profiling data generated by FMI were reviewed, and the MTB assessed whether these results would support, refine, or change the guideline-based treatment strategy, classified as major, minor, confirmatory, or no evidence for treatment modification. This step reflected the incorporation of a clinically implemented genomic platform used in some clinical centers, allowing assessment of how useful additional genomic information was in refining standard-of-care treatment decisions. Third, the integrated TuPro multi-omics and functional data were discussed, which represent the new and unique opportunity of the TuPro study. The MTB then re-evaluated the case and determined whether the TuPro dataset provided supporting or conflicting evidence that might alter the therapeutic recommendation made in the previous two steps. Across all cases, analysis of the decision reports revealed that each TuPro technology contributed to at least one MTB by providing supportive biomarkers or functional validation from ex vivo assays. Several biomarkers were recurrently considered in treatment discussions, with *BRCA1/2* status, phosphorylated mTOR (pmTOR), and γH2AX among the most influential (Fig. 2A). Overall, the inclusion of TuPro data significantly increased the proportion of patients with a minor treatment change (drug change within the same drug class) from 4% to 37% (Pearson’s Chi-squared test: χ² = 15.35, df = 1, *p* = 8.9 × 10⁻⁵), while reducing cases with no evidence for a change from 51% to 8% (χ² = 20.83, df = 1, *p* = 5.0 × 10⁻⁶) (Fig. 2B and Supplementary Fig. 4A). When considering changes of a drug class by either replacing, removing, or adding a new treatment (‘major treatment change’), we observed a moderate increase in the likelihood of a treatment change from 24% using FMI only (*p* = 0.14) to 39% using TuPro technologies (Fig. 2B). Importantly, these recommendations were independent of clinical treatment setting (chemo-naive, post-NACT, or recurrent disease) (Pearson’s Chi-squared test: X^2^ = 2.51, df = 4, *p* = 0.64; X^2^ = 5.60, df = 4, *p* = 0.23, respectively) (Fig. 2C). The molecular summary report provided at each MTB was considered clinically relevant for the majority of patients (‘very’ and ‘largely useful, 30/51, 60.1%, and Fig. 2D, see Methods for details). In 21 out of 39 cases (54%) where TuPro altered the treatment decision, this change was also reflected in the physician’s final treatment choice. As this trial was an exploratory setup, from an ethical point of view, we could not change acute therapies in the frontline or second-line setting. In addition, the study cohort lacked a significant number of patients beyond standard-of-care treatment, where such modifications could have been ethically permissible. Therefore, the only adaptations made based on the MTB in this trial, were in regards to the maintenance therapy following standard-of-care chemotherapy. These alterations were involving the choice and type of PARPi, or the addition of letrozole, metformin or bevacizumab (Fig. 2E, F, see Supplementary Data 12 for details). In the group of newly diagnosed patients (chemo-naïve or post-NACT, FIGO stages III or IV) this maintenance alteration led to a significant overall survival benefit in patients compared to patients where TuPro did not alter the treatment (unadjusted Cox model, log-rank *p* = 0.007; HR = 0.30 [0.12-0.75], df = 1; Cox model adjusting for age, FIGO Stage, residual disease, LOH and treatment status *p* = 0.07, HR = 0.27 [0.064-1.12], df = 8); Fig. 2E and Supplementary Fig. 4B). While this represents a first indication that should be interpreted with caution given the lack of randomization, these findings suggest a potential clinical impact of TuPro-guided treatment recommendations.

In summary, our proposed framework for evaluating the clinical usefulness of routine and emerging diagnostics in combination with experimental multi-modal tumor profiling in a representative HGSOC cohort enabled personalized drug recommendations independently of treatment status. This approach demonstrates a feasible precision-oncology workflow that can inform treatment decisions beyond standard-of-care therapy.

### Whole-genome amplification predicts poor outcome

**Fig. 3:**
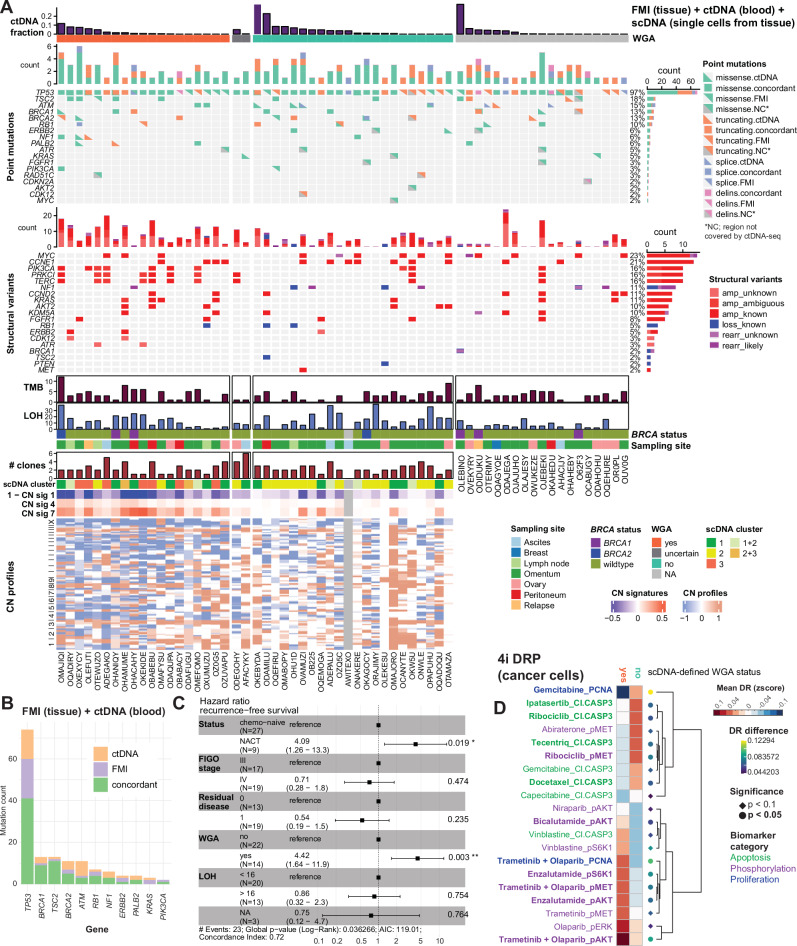
Trimodal assessment of the cancer genome identifies WGA as a predictor of poor outcome and an indicator of altered ex vivo drug response. **A** Integrative oncoprint displaying the genomic landscape of HGSOC defined by the FoundationOne^Ⓡ^CDx (FMI) targeted sequencing panel, targeted ctDNA sequencing, and scDNA-seq, ordered by WGA status. The top panel shows mutations detected by FMI and ctDNA; the middle panel shows structural variants identified by FMI; and the lower panel displays the average copy number (CN) profiles of the tumor clones detected by scDNA. Gray indicates missing data. **B** Bar charts comparing point mutations identified in cancer specimens (FMI) and matched blood-derived samples (ctDNA). **C** Forest plot showing hazard ratios and 95% confidence intervals for recurrence-free survival. **D** Dendrogram comparing WGA groups using 4iDRP readouts. Groups were compared using the Kruskal-Wallis Test with multiple-testing correction (Tukey-Kramer) per drug. The accompanying heatmap shows the mean of each 4iDRP for both WGA groups (yes vs. no), for which the *p*-value was below 0.1. Each 4iDRP feature is characterized by a drug name followed by an underscore (“_”) and a 4i readout, and represents the respective marker intensity in this drug condition relative to DMSO control. 4iDRP features were clustered using hierarchical clustering. *P*-values achieved by each 4iDRP feature are displayed as circles or rhomboids at the leaf end of the dendrogram. See also Supplementary Figs. 5, 6. Source data are provided as a Source Data file.

HGSOC carry *TP53* mutations and are enriched with amplifications of 3q26 (OncCassette^53^) and 8q24/*MYC*. These tumors are also characterized by losses of chromosome arms 4q, 5q, 8p, and 18q^27^, and harbor inactivating mutations in genes associated with HR repair^54^. We first studied the genetic landscape of the TuPro HGSOC cohort by incorporating results obtained from FMI, scDNA-seq and ctDNA-seq. The majority of the cohort harbored *TP53* mutations (97%) in at least one of the two applied NGS technologies (Fig. 3A). Additional mutations that occurred at a rate of 10–20% in the entire TuPro cohort were observed in *TSC2*, *ATM*, *BRCA1*, and *RB1*. *MYC* (23%) and *CCNE1* (21%) were the most commonly amplified genes in tumor samples (Fig. 3A). Blood plasma and tumor specimens taken at the same time and measured by FMI and ctDNA-seq, respectively, resulted in 55.4% concordant and 44.6% discordant findings considering the ctDNA-seq gene panel (Fig. 3B). The mutations detected by ctDNA-seq varied across different sampling time points, potentially recapitulating tumor burden and response to treatment (Supplementary Fig. 5A).

The genomic profile described in our cohort was strongly correlated with the TCGA ovarian cancer project^55^ (Pearson correlation, *r* = 0.95, *p* = 2.5 × 10^−15^, df = 50) (Supplementary Fig. 5B, C). In addition, scDNA-seq analysis revealed two distinct patterns: (1) patient-specific clustering of aneuploid cells and (2) a lack of discernible distinctions among diploid cells. These results indicate that each tumor is genomically unique, and no commonly shared CN profiles are observed between patient samples (Supplementary Fig. 5D, E). When employing scDNA-seq and comparing analyses that include whole-genome amplification (WGA) to those that exclude this event (see “Methods”), we identified 22 samples (43.1%) with aneuploid cells harboring WGA. Similar to previous approaches^56^, we compared CN changes relative to diploid or tetraploid references, however, with scDNA-seq, we resolved CN at the single-cell level, directly accounting for tumor purity and subclonal structure. This contrasts with bulk sequencing approaches^57^, where whole-genome duplication detection is influenced by these factors and requires computational modeling^56^. Notably, the subgroup harboring WGA displayed an enrichment of tumor samples characterized by an elevated number of amplified genes, with an average of 52% of the genome affected by CNAs compared to 22% in samples without WGA (*t* = 3.8, df = 35, *p* = 4.9 × 10⁻⁴), underscoring the increased genomic instability associated with WGA. We did not find any statistically significant evidence that the increased genome amplification was affected by tumor mutational burden (TMB) and LOH (both not significant in a multiple linear regression, with regression coefficients of 0.012 and 0.004, respectively and *p* = 0.5, compared to the adjusted effect of 0.29 of WGA with *p* = 0.0017). In general, scDNA-seq revealed a diverse genomic landscape with frequent CNAs, a high degree of inter-patient and intra-tumor heterogeneity harboring diverse aneuploid clones (Supplementary Fig. 5E). Along with analyzing point mutations and structural variants detected by both targeted NGS platforms and scDNA-seq, we also investigated the presence of previously identified CN signatures^58^ in our scDNA-seq data (see “Methods”). The entire cohort of aneuploid cells was predominantly characterized by CN signature 1, which is defined by a low number of breakpoints (up to 2 breakpoints per chromosome arm) and large segment sizes (> 30 Mb), with a mean of 0.81 (*t* = 29, df = 50, *p* < 1 × 10^−16^ compared to uniform distribution across the seven CN signatures). This finding may result from the signatures having been trained on bulk data. However, CN signature 4 (high segment CN, 4-8 copies, and CN changes of 2-3 copies) and signature 7 (CN changes from tetraploid to 3 copies and breaks distributed evenly across the genome) were enriched in samples with WGA (*t* = 2.9, df = 28, *p* = 6.7 × 10^−3^; and *t *= 2.2, df = 43, *p* = 0.035, respectively). In addition, scDNA-seq cluster 2 appeared less frequently, while cluster 3 was more prevalent in samples with WGA (*p* = 5.9 × 10^−4^, Fisher’s exact test on the 2 × 5 contingency table) (Fig. 3A). This seems to be clinically important, since all newly diagnosed patients harboring WGA experienced an earlier disease recurrence compared to those without WGA (log-rank, *p* = 0.0017) (Supplementary Fig. 5F). We further constructed a multivariate Cox proportional hazard model, including FIGO stage, residual disease and LOH, with relapse-free survival as the outcome. Using this model, we found that WGA and samples taken post-NACT were the strongest predictors of poor outcome (Fig. 3C). Next, we explored whether samples with WGA displayed altered gene and protein expression. Only marginal molecular differences were observed across omics-technologies comparing scDNA-seq groups (Supplementary Fig. 6), but we noticed significant differences in 4i DRP ex vivo drug responses. Samples with WGA showed increased mean drug response scores for 6 of 45 tested drug-marker combinations (Kruskal-Wallis Test, *p* < 0.05), using the cell signaling (phosphorylation) associated readouts pAKT, pS6K1, and pMET and the proliferation marker PCNA. For samples without WGA, we noticed that cleaved caspase 3, an apoptosis marker, was the primary elevated readout in 5 out of 45 tested drugs (Kruskal-Wallis Test, *p* < 0.05). These results point towards WGA-dependent ex vivo drug responses (Fig. 3D).

Our trimodal genetic analysis of HGSOC, utilizing a combination of NGS technologies, revealed a predominance of *TP53* mutations accompanied by common amplifications in *MYC* and *CCNE1*. The analysis also showed a heterogeneous genomic landscape characterized by frequent CN alterations. The identification of WGA in a subgroup of patients was associated with earlier disease recurrence and altered drug response profiles. Collectively, these findings highlight WGA as a potential predictive biomarker with meaningful clinical and therapeutic implications for patients with HGSOC.

### TME diversity outweighs cancer cell variation across sample sites

**Fig. 4:**
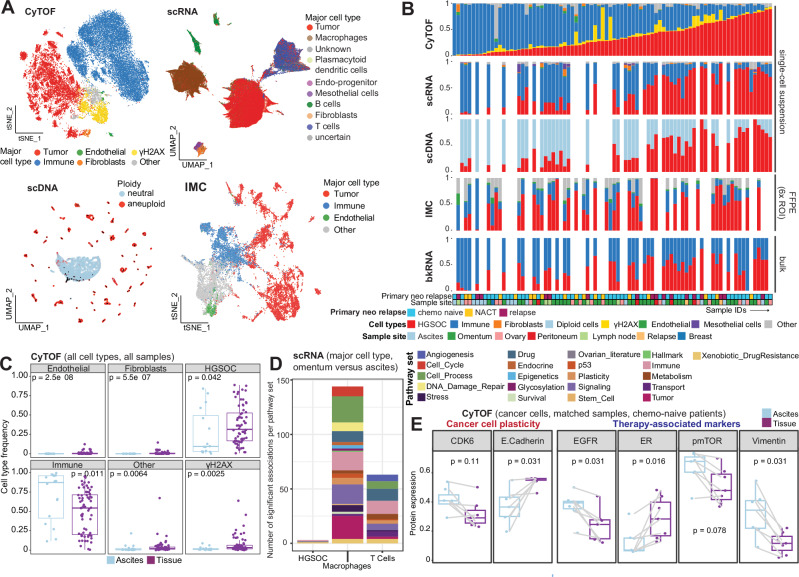
Distinct immune microenvironment and tumor cell heterogeneity in ascites compared to solid tumor tissue. **A** Dimensionality reduction of all single-cell technologies in the HGSOC cohort using non-linear methods (UMAP and t-SNE). Major cell types identified by each technology are shown alongside their respective cluster embeddings. **B** Stacked bar chart sorted by increasing tumor (HGSOC) cell content as quantified by CyTOF, aligned with other technologies reporting at least two cell types, including tumor cells. **C** Distribution of major cell types derived from CyTOF, comparing samples originated from ascites versus solid tumor tissue. Groups were compared using a Wilcoxon rank-sum test; box plots are formatted as in Fig. 2F. **D** Number of significantly differentially activated pathways between ascites and omentum samples (FDR < 0.1), per pathway set and cell type. Pathway scores are computed from bulkified scRNA-seq data per cell type and sample. All available samples were included irrespective of treatment status. **E** Differentially expressed markers on cancer cells derived from matched ascites and solid tumor tissue samples of the same chemo-naive patient. CyTOF-derived groups were compared using a Wilcoxon signed-rank test; box plots follow the format in Fig. 2F, with lines connecting matched samples. See also Supplementary Fig. 7, Supplementary Table 1, 2 and Supplementary Data 13. Source data are provided as a Source Data file.

Cell types in the TME are strongly influenced by the anatomical site and the mutational processes to which they are exposed^51^. Here, we constructed individual cell type maps derived from each single-cell technology (CyTOF, scRNA-seq, scDNA-seq, IMC, and PCY). These included HGSOC and non-malignant cells, such as immune cell types (lymphocytes and myeloid cells), as well as fibroblasts, endothelial and mesothelial cells (Fig. 4A). Each TuPro approach employs distinct, system-specific markers, either direct gene expression measurements or antibody-based detection, to identify specific cell types. Despite these methodological differences, we observed a consistent sample composition regarding the major cell types across approaches, based on overlapping markers (Fig. 4B and Supplementary Fig. 7A, B). The quantified tumor content of each sample compared across technologies revealed moderate to strong correlations for fresh sample-derived viable single-cells (mean Pearson’s *R* = 0.82) (Supplementary Fig. 7C). However, correlations between sample-derived single-cell suspensions and FFPE were weak to moderate (mean Pearson’s *R* = 0.29). This is not unexpected, as these samples were processed and stored in different ways. In addition, tissue heterogeneity likely contributes to this discrepancy - technologies using FFPE samples analyze pre-defined regions of interest, which may explain the lower correlations to the single-cell suspensions, as well as the variability among the three FFPE-based technologies (FMI, DigiPath, IMC; mean Person’s *R* = 0.62). Since our cohort comprises heterogeneous sample types (Supplementary Table 2) and the literature reports on a distinct cell repertoire in ascites compared to solid tumor tissue samples^51,59^, we compared cell composition and molecular profiles between sample types. Due to priority technology ranking and the available resources (Supplementary Table 1), the most comprehensively analyzed dataset was CyTOF (*n* = 76 samples). It revealed an enrichment of immune cells in ascites, while endothelial, fibroblasts, and DNA-damaged pH2AX^+^ cells were almost absent (Fig. 4C). To further characterize cells within the TME, we examined the molecular characteristics of distinct cell types within specific sample types. Using a curated set of 2,896 GeneSets categorized into 22 groups based on their property (Supplementary Data 13), we assessed by scRNA-seq significant differential pathway activation (DESeq false discovery rate (FDR) < 0.1 per customized GeneSet category). Our analysis focused on ascites and omentum samples of the entire cohort, highlighting major cell types like T cells, macrophages, and HGSOC cells (Fig. 4D). In these cell populations, we observed significant differences in pathway activation, predominantly in macrophages (*n* = 144 pathways/ 104 unique) and T cells (*n* = 63/ 43 unique pathways). In contrast, HGSOC cells displayed minimal variation comparing ascites and omental metastases with only three distinct GeneSets: *‘GOBP: MHC class II biosynthetic process’*, *‘Xenobiotic drug resistance’* (up in ascites), and *McCollum: Geldanamycin resistance up* (down in ascites)’, and *‘Literature: Stress_associated*^60^ (down in ascites)’ (see source data for details). From this observation, we conclude that the transcriptional changes are modest when comparing ascites- and solid tissue-derived cancer cells. Consistent with these findings across sample sites at the cohort level, CyTOF revealed only mild differences in HGSOC marker expression (Supplementary Fig. 7D). However, significant disparities were detected for cancer cell plasticity protein markers E-cadherin and Vimentin^61^, along with therapy-associated markers analyzing inter-site compositional variation within patients at the time of diagnosis (Fig. 4E and Supplementary Fig. 7D). A similar pattern was observed for PCY-derived ex vivo drug responses, where global cohort analysis revealed significantly higher drug response similarities for patient-matched samples compared to patient-unmatched samples regardless of sample site (Supplementary Fig. 7E). Analysis of matched samples taken at the same time revealed similar drug responses with only a trend towards higher cancer-specific response to rucaparib in ascites compared to tissue (Supplementary Fig. 7F, G). In contrast to cancer cells, the immune compartment demonstrated differences in the cell fractions. Plasmacytoid dendritic cells (pDC) were elevated in ascites, whereas plasma cells, exhausted CD8 T cells, and regulatory T cells were more abundant in solid tissue (Supplementary Fig. 7H). Together, these data suggest that inter-patient differences in the cancer cells exceed subtle differences between sample sites within individual patients.

### HGSOC molecular subtypes are defined by the TME cell composition and cancer cell-specific traits

**Fig. 5:**
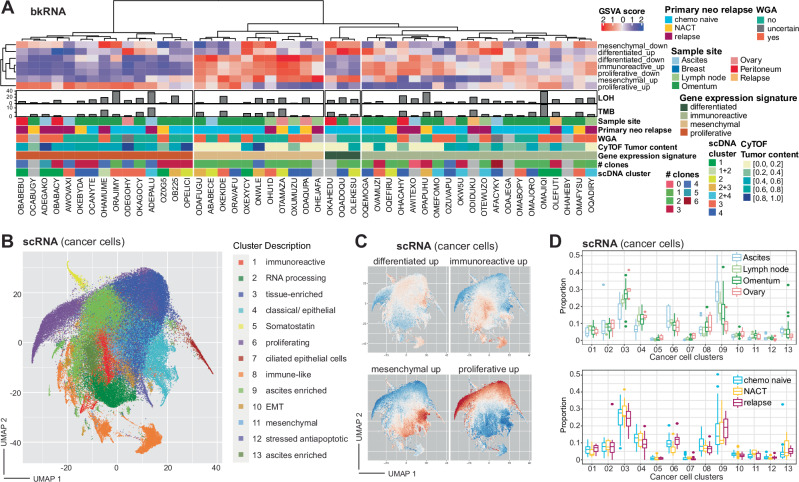
Diverse gene expression profiles within tumors reflect TME composition and extensive cancer cell heterogeneity. **A** BulkRNA-seq-derived clustering of samples based on their GeneSet activation scores for the mesenchymal, differentiated, immunoreactive, and proliferative signatures (each separated into up- and down-regulated components) - Clustering was performed using Euclidean distance metric and Ward’s method. **B** UMAP visualization of all HGSOC cells from the entire TuPro cohort, colored by Louvain-based unsupervised clustering. Clusters were manually annotated based on their characteristic gene expression signatures. **C** UMAP representation of HGSOC cells, as in panel B, colored by relative activation of the mesenchymal, differentiated, immunoreactive, and proliferative subtype signatures. **D** Box plots (formatted as in Fig. 2F) showing tumor cell proportions per cluster for each patient, separated by tissue type (top) and by clinical setting (primary tumor, neoadjuvant, or relapse; bottom). See also Supplementary Fig. 8 to 10. Source data are provided as a Source Data file.

The literature describes four molecular HGSOC subtypes based on bulk transcriptomics^49^. These have been widely studied and associated with distinct tissue origins, clinical traits, tumor pathology, and unique TME compositions^49,62–66^. To integrate these previously proposed subtypes^67^ into our analysis and assess their clinical significance, we applied the *‘subtype gene expression signatures’* from Konecny et al.^48^ to our bkRNA-seq data. Unsupervised clustering classified tumor samples into all four known subtypes: immunoreactive, mesenchymal, differentiated, and proliferative. Consistent with previous reports, the proliferative subtype was notably distinct from the others^48^ (Fig. 5A). Moreover, in line with prior findings^68^, the *‘subtype gene expression signatures’* were influenced by the sample’s cell type composition, which also demonstrated robustness across different technologies (Supplementary Fig. 8A-E). Next, we integrated the *‘subtype gene expression signatures’* into scRNA-seq data. Not surprisingly, when considering all of the cell types, the *‘proliferative signature’* was primarily elevated in a distinct cancer cell population, whereas the *‘immunoreactive signature’* was enriched in immune cells. Interestingly, all *‘subtype gene expression signatures’* were also detectable within the cancer cell population (Supplementary Fig. 8F), suggesting that ‘*subtype gene expression signatures’* are not solely driven by overall TME composition. We therefore hypothesized that these signatures also reflect intrinsic characteristics of the cancer cells themselves. Thus, we performed a subcluster analysis on the scRNA-seq (*n* = 55 samples) data and compiled a cancer cell atlas in this cohort cell subpopulation. Unsupervised subclustering of scRNA-seq-derived cancer cells identified 13 distinct clusters, each exhibiting unique gene expression profiles and GeneSet signatures, highlighting the heterogeneity of cancer cells in this cohort (Fig. 5B, Supplementary Fig. 9, Supplementary Fig. 10). From these, large clusters such as cluster 3 (tissue-enriched) and cluster 9 (ascites-enriched) lacked specific pathway activity, whereas medium-sized and smaller clusters displayed distinct functional characteristics, including proliferative (cluster 6), immune-like (cluster 8), epithelial (cluster 4), and stem-like (cluster 11) signatures, supporting the diverse cellular states of cancer cells within the TME (see Supplementary scRNA-seq cluster description for more details). This analysis further confirmed that all four *‘subtype gene expression signatures’* were also present within cancer cells, and associated with distinct cell clusters (Fig. 5B-C). Interestingly, each HGSOC sample was represented in virtually all unsupervised clusters, indicating that each tumor biospecimen intrinsically contains a heterogeneous mix of cancer cells in varying phenotypic states (Supplementary Fig. 10B). Apart from sample site-specific alterations in cancer cell cluster proportions (Fig. 5D, top panel), incorporating clinico-pathological data also revealed profound differences for chemotherapy status. Here, the proportion of cells in the proliferative cluster 6 was reduced in post-NACT samples, demonstrating the impact of chemotherapy on dividing cancer cells (Fig. 5D, bottom panel). In summary, these findings suggest that cancer cell heterogeneity is intrinsically embedded within each tumor, with individual cancer cells exhibiting varying degrees of all molecular HGSOC subtype characteristics. This cancer cell heterogeneity is further modulated by the TME, sample site, and chemotherapy status, as reported by shifts in cancer cell cluster proportions.

### Drug sensitization and cell cycle arrest in cancer cells occurs post-neoadjuvant chemotherapy

**Fig. 6:**
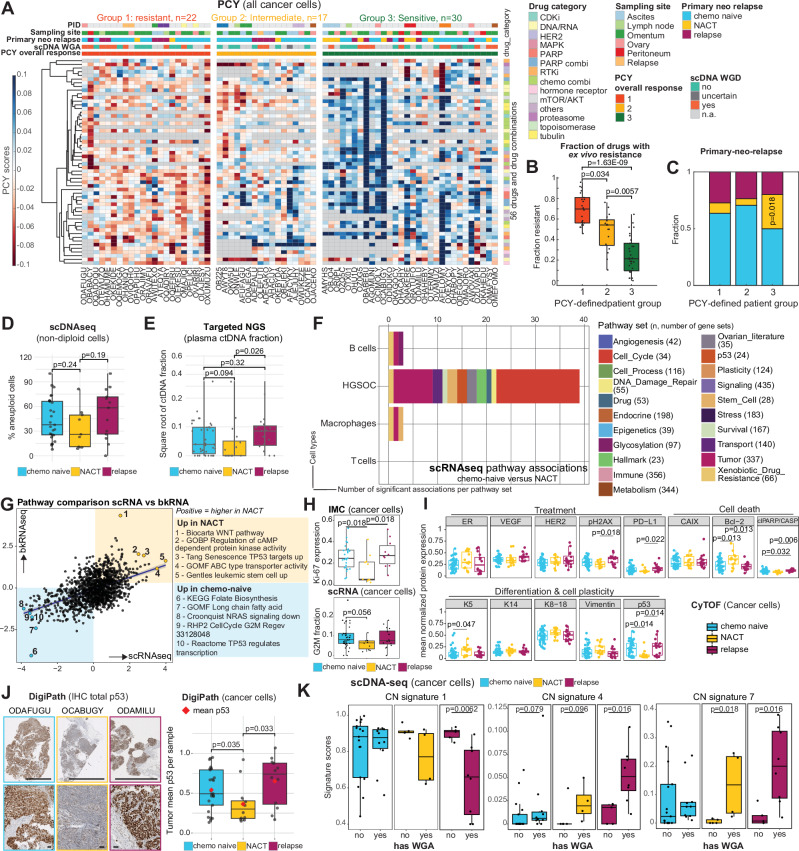
Neoadjuvant chemotherapy sensitizes cancer cells ex vivo and alters cell cycle and plasticity pathways. **A** Cohort-wide heatmap of PCY-based ex vivo drug responses (PCY scores; see methods) across 69 biopsies from 54 patients and 54 drugs and drug combinations. Drugs with a similar mode of action show consistent drug responses across samples, with PARP inhibitors emerging as the class of drugs which most often leads to an on-target effect. The row dendrogram is calculated based on cosine distance, using average linkage. Columns (samples) are stratified into 3 groups based on spectral clustering (cosine distance), sample order within each cluster is arbitrary. **B** Fraction of drugs showing ex vivo resistance separates patient samples into three groups (box plot, *p*-values from Tukey’s honestly significant difference test). **C** Stacked barchart shows significant enrichment (hypergeometric test, *p* = 0.018) of neoadjuvant treated samples in ex vivo drug response sensitive group 3. **D** Box plot with a reduced percentage of malignant cells in neoadjuvant samples identified by scDNA-seq. **E** Box plot with reduced total ctDNA derived from blood in neoadjuvant samples. **F** Comparison of differential pathway activation between chemo-naive and NACT was conducted separately in bulk and scRNA-seq, followed by integration. Each dot corresponds to a pathway, the values shown are the *t*-statistics obtained from a linear regression model. Positive values indicate increased pathway scores in samples taken after NACT. **G** Differential pathway activation analysis comparing NACT to chemo-naive samples. Values represent the *t*-statistic of a linear regression model applied to pathway scores derived from bulkified scRNA-seq gene expression in HGSOC cells (x-axis) and bulk RNA-seq (y-axis). Selected pathways are annotated. **H** Cancer cell-specific comparison for the proliferation marker Ki-67 and a fraction of cancer cells in the G2M cell cycle phase using IMC (Dunn test with the BH method to correct for multiple testing across treatments and scRNA-seq (Wilcoxon rank-sum test), respectively. **I** Box plot displaying the mean normalized expression of the indicated markers across chemo naive, post-NACT and relapse samples. CyTOF-derived groups were compared using a Dunn test with the BH method to correct for multiple testing across treatments. **J** Representative immunohistochemistry p53 staining and corresponding quantification for chemo-naive, post-NACT, and relapse samples, mean p53 expression per sample, pairwise Wilcoxon rank-sum test comparing the three groups. **K** CN signatures scores (1, 4, and 7) stratified by the presence or absence of WGA and further separated by clinical stage. Relapse samples with the strongest CN signature differences between WGA-positive and -negative tumors, with smaller effects in NACT samples and marginal differences in chemo-naive samples. *P*-values from Wilcoxon rank-sum test. All box plots are formatted as in Fig. 2F. See also Fig. 3A, Supplementary Fig. 11 and Supplementary Data 10. Source data are provided as a Source Data file.

To characterize the drug response landscape in HGSOC and link it to clinical and molecular features, we analyzed a total of 69 specimens from 54 patients by PCY^42–44^. PCY employs automated microscopy and computational analysis to assess drug responses in patient samples after 24 hours of culture, testing 54 pre-selected drug compounds (Fig. 6A and Supplementary Data 10). Notably, the samples exhibited distinct sensitivity or resistance patterns independent of the specific drug tested. To stratify the cohort based on these global drug response patterns, we assigned the samples into one of 3 groups based on unsupervised clustering of their ex vivo responses. A subset of samples displayed resistance (Group 1, *n* = 22 samples), while another demonstrated responsiveness to most of the tested compounds (Group 3, *n* = 30 samples) (Fig. 6B). This pattern may indicate a common mechanism of drug response against specific drugs across different patient samples. Indeed, pathway analysis of single-cell and bkRNA-seq data revealed an association between this multi-drug resistance phenotype and pathways related to poor survival, cisplatin resistance, and EMT (Supplementary Fig. 11A).

In the context of HGSOC, patients may receive NACT with carboplatin and paclitaxel to reduce tumor burden before surgery^69^. This treatment regimen is particularly relevant for studying the impact of chemotherapy. Our data indicate that samples taken post-NACT were significantly enriched in Group 3 (hypergeometric test, *p* = 0.018, 9/12 neo-adjuvant samples in group 3), suggesting that NACT sensitizes tumor cells to subsequent treatments ex vivo (Fig. 6C and Supplementary Fig. 11B). To gain a deeper understanding of the cellular and molecular changes induced by chemotherapy, we conducted a multi-technology comparison within our cohort. As expected, measures associated with tumor burden notably decreased following NACT. This was evident in both the reduced numbers of malignant cells per sample (Fig. 6D and Supplementary Fig. 11C), a decline in ctDNA levels (Fig. 6E), as well as elevated exhausted CD4 + T cells, myeloid, and pDCs determined in PBMCs (Supplementary Fig. 11D). Pathway analysis of scRNA-seq data demonstrated that most changes occurred in cancer cells and were associated with cell cycle, tumorigenesis, and p53 signaling (Fig. 6F), consistent with the known cytoreductive effects of chemotherapy on highly proliferating tumor cells. These differential pathway activations were consistent also with bkRNA-seq results, where the number of genes with gene expression estimates is typically higher (Fig. 6G). Increased post-NACT pathway activation affected cell plasticity (cAMP-dependent kinase signaling) and stemness (Wnt signaling and leukemic stem cell signature), transmembrane transporter activity, as well as p53-mediated senescence. Decreased activation was found for pathways related to DNA synthesis and cell proliferation. The downregulation of cell cycle-associated GeneSets reported here is further supported by previous studies using scRNA-seq in longitudinal HGSOC post-NACT samples^60^. This finding was supported in our cohort by decreased Ki-67 and a reduction in cancer cell numbers in the G2 phase of the cell cycle, as observed in IMC and scRNA-seq, respectively (Fig. 6H). Further investigation on protein abundances measured by CyTOF revealed marginal alterations in the expression of treatment-relevant markers in post-NACT compared to chemo-naive samples. Cell death markers remained largely unchanged, except for Bcl-2, which was elevated in NACT samples. However, alterations were observed in differentiation and cell plasticity markers, with an increase in keratin 5 post-NACT (Fig. 6I). Coupled with heightened activity of Wnt signaling at the RNA level, these changes may suggest a transition towards a more mesenchymal cell state, indicating a treatment-specific adaptation or escape. Furthermore, there was a significant downregulation of total p53 in the NACT group compared to both chemo-naive and relapse samples (Fig. 6I). Given the crucial role of p53 in orchestrating G1 cell cycle arrest, apoptosis and cell cycle regulation^70^, and considering that most HGSOC harbor *TP53* mutations, we performed additional quantification of total p53 expression in whole FFPE tissue sections (*n* = 56 samples). This analysis confirmed the decrease in p53 expression post-NACT (Fig. 6J). The presence of a mixture of CN signatures in most HGSOC suggests that various mutational processes are at work. The occurrence of early *TP53* mutations, which is an initiating event in HGSOC, may facilitate the co-evolution of multiple mutational processes^58^. In response to these findings, we investigated the effect of chemotherapy (post-NACT and relapse) on the genomic signatures using matched scDNA-seq (Fig. 3A) and in the context of WGA. We observed differential scores for CN signatures 1, 4, and 7^58^. Interestingly, only samples post-NACT and relapse showed profound alterations in CN signature scores in the presence of WGA, while chemo-naive samples were marginally affected (Fig. 6K). Together, these data may support the co-existence of multiple genomic processes in the genetic background of *TP53* mutations. This further highlights the potential impact of WGA and chemotherapy-induced genomic instability as a result of treatment-induced DNA damage and tumor evolution.

### Increased phenotypic divergence and WGA are common in HGSOC recurrence

**Fig. 7:**
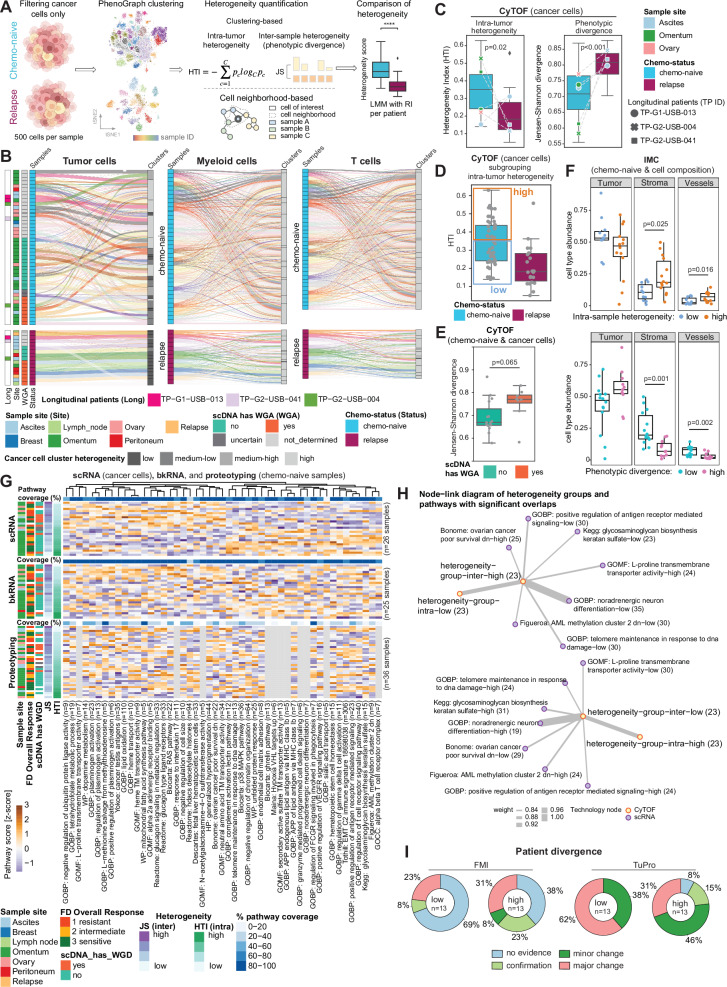
Chemotherapy pretreatment increases patient-specific cancer cell heterogeneity. **A** Schematic overview of the tumor heterogeneity assessment workflow: data filtering and subsampling, per-group phenograph clustering, heterogeneity quantification and statistical comparison of heterogeneity across the groups using a Linear Mixed Model (LMM). **B** Sankey diagram depicting heterogeneity of tumor cells (left), myeloid cells (middle) and T-cells (right) as captured by CyTOF. **C** Box plot comparing chemo-naive and relapse for calculated intra-tumor (HTI, LMM *p* = 1.7 × 10^-4^) and inter-sample (JS, LMM *p* = 3.4 × 10^-6^) heterogeneity. **D** Box plot showing median-cut stratification of chemo-naive samples into low- and high-complexity phenotypic groups. **E** Corresponding box plot of chemo-naive group with WGA defined (yes/no) with JS divergence being elevated in WGA samples with borderline significance, Mann-Whitney U test (“no” median of 0.67 (IQR: [0.66, 0.73]), “yes” median of 0.77 (IQR: [0.75, 0.79])). **F** IMC-derived cell-type proportions in chemo-naive samples stratified by low and high phenotypic complexity displayed for significant cell type proportions for intra-tumor heterogeneity (HTI) and phenotypic divergence. **G** Heatmaps showing pathway scores calculated on: the bulkified gene expression in HGSOC cells by scRNA-seq (top); tumor-content corrected gene expression by bulk RNA-seq (middle); bulk protein abundance by proteotyping (bottom). GeneSets were selected based on association with inter- and intra-tumor heterogeneity in scRNA-seq and bulk RNA-seq, respectively. The number of genes per pathway is written in brackets, the blue colorbar indicates the pathway coverage (percentage of genes detected for this pathway) per technology. Pathways are ordered based on hierarchical clustering on the scRNA-seq pathway scores. **H** Node-link diagram showing statistically significant (FDR < 0.05) similarities between patient groups based on tumor heterogeneity as determined by CyTOF analysis and pathway activity as assessed by scRNA-seq. Similarity is quantified using the overlap coefficient, with the weights of the links in the diagram indicating the degree of similarity. Statistical significance is determined through permutation testing. **I** Retrospective evaluation of MTB reports for chemo-naive HGSOC patients (*n* = 26). Pie charts summarize treatment recommendations based on routine clinical assessment, after presentation of FMI data, and after consideration of the full TuPro dataset stratified by CyToF-derived low and high patient divergence. All box plots are formatted as in Fig. 2F. See also Supplementary Figs. 12–14. Source data are provided as a Source Data file.

Several key factors have been described for disease progression in HGSOC: the emergence of platinum resistance from minor pre-existing populations, dynamic mutational processes that drive metastatic dissemination, and the persistence of stress-associated states following chemotherapy^60,71–75^. The profound multimodal differences observed in post-NACT samples prompted us to explore cancer cell heterogeneity. We noticed patient-specific clustering in both post-NACT and in relapsed patient samples, as revealed by our explorative unsupervised cluster analysis in CyTOF and matched IMC data (Supplementary Fig. 12). We quantified intra-tumor heterogeneity and phenotypic divergence (heterogeneity of the cohort) using the Shannon entropy and Jensen-Shannon (JS) divergence, respectively. Linear mixed models, accounting for potential intra-patient sample similarity by using a random intercept for each patient, were employed to compare heterogeneity scores across clinically defined groups (Fig. 7A). This analysis of tumor cell clusters based on CyTOF protein expression profiles (cancer antibody panel), termed tumor phenotypes, revealed a lower phenotypic complexity in relapse compared to the chemo-naive group. Specifically, samples from relapsed patients were associated with fewer distinct clusters (median number of distinct clusters in relapse patients are 10.5 compared to 13 in chemo-naive samples), whereas chemo-naive samples exhibited multiple connections from one sample leading to several clusters. Importantly, these patterns were characteristic only for cancer cells. In contrast, both T and myeloid cells demonstrated high inter-sample mixing and intra-sample heterogeneity across chemo-naive and relapse subgroups (Fig. 7B). When comparing heterogeneity scores calculated on cancer cells using a linear mixed model (LMM), we found significantly reduced intra-tumor heterogeneity (heterogeneity index (HTI), *p* < 0.001) and higher inter-sample divergence (JS, *p* < 0.001) in relapse samples compared to the chemo-naive group. Moreover, this pattern was consistent in three patients with longitudinal samples taken at both the time of initial diagnosis and disease recurrence (Fig. 7C and Supplementary Fig. 13A). Reduced intra-tumor heterogeneity in relapse samples was also reflected in the lower number of clusters needed to capture 90% of sample cells (Supplementary Fig. 13B). To mitigate any effects that could arise from sample site imbalances between the groups, we performed a more homogeneous subgroup analysis focusing on ascites samples only. This analysis further supported that relapse samples exhibit lower intra-tumor heterogeneity (HTI, *p* = 0.02) and elevated phenotypic divergence (JS, *p* < 0.001) (Supplementary Fig. 13B, C and Supplementary Fig. 14A). To better understand these findings, we stratified chemo-naive samples based on the median HTI score (Fig. 7D). This analysis indicated that elevated phenotypic divergence tends to coincide with the presence of WGA, albeit not statistically significant (two-sided Wilcoxon rank-sum test, difference in medians [95% CI] = -0.09 [-0.13, 0.003], *p* = 0.065) (Fig. 7E). In addition, we observed an altered cell type composition, characterized by an enrichment of stroma cells and blood vessels in samples with high intra-tumor heterogeneity and low phenotypic divergence. In contrast, myeloid and lymphoid cells exhibited marginal opposite trends using CyTOF data (Fig. 7F and Supplementary Fig. 14B). The selection of GeneSets in both bkRNA-seq and scRNA-seq revealed immune-like, metabolic and transmembrane transport GeneSets that distinguish chemo-naive patients with low and high intra-tumor heterogeneity (Fig. 7G). Integrative analysis of clinical and derived molecular features further strengthened the association of specific GeneSets, WGA, and structural variants with intra-tumor heterogeneity (Fig. 7H and Supplementary Fig. 15). Finally, we investigated whether prior knowledge of CyTOF-derived patient divergence would alter treatment recommendations given in MTBs. Interestingly, almost all newly diagnosed chemo-naive patients exhibited changes in treatment recommendations, ranging from minor to major. Notably, patients with high divergence, characterized by sharing fewer tumor clusters with the entire cohort, could benefit from FMI data alone. Conversely, all thirteen patients with low phenotypic divergence experienced changes in their treatment recommendations considering TuPro data (Fig. 7I).

## Discussion

The Swiss TuPro observational research project assessed multi-omics data from newly diagnosed and recurrent patients with HGSOC, incorporating up to eleven technologies providing insights into the TME and blood at the functional, DNA, RNA and protein level. The incorporation of multi-omics data into MTB - beyond genomic analysis - was feasible within a clinically relevant turnaround time of 4 weeks, and considered termed “useful” by the MTB attendants. Our study identified significant molecular and cellular variations within the patient cohort, influenced by factors such as sample type, collection timing during disease progression, as well as chemotherapy status — ranging from treatment-naïve to post-NACT chemotherapy and recurrent disease.

We explored differences in cancer cell populations across various matched sample sites using the CyTOF dataset, which includes the largest number of profiled samples. Our cohort analysis revealed diverse expression patterns of EMT and therapy response markers, including EGFR, phospho-mammalian target of rapamycin (pmTOR), and CDK6. Comparison between tissue- and ascites-derived cancer cells across the cohort revealed only marginal differences in gene- and protein expression as well as ex vivo drug responses. Samples from the same patient were more similar to each other compared to patient-unmatched samples, suggesting that inter-patient differences exceed subtle differences between sample sites within individuals. We therefore investigated sample site differences within a limited set of patient-matched samples, which revealed more pronounced differential expression patterns between cancer cells derived from ascites and those from solid tumor tissue. The mesenchymal markers and EGFR reported herein have been shown to be upregulated and activated in cancer cells grown in suspension^76^, suggesting that ascites-derived cancer cells may undergo an EGFR-driven EMT-like transition compared to those from solid tumors. This may imply that treatment-relevant markers could differ between ascites and solid tumors, potentially informing the use of targeted therapies such as EGFR inhibitors (e.g., gefitinib, erlotinib^77,78^), particularly in recurrent cases with the presence of ascites. Of note, a limited number of these paired samples showed marginal differences in ex vivo drug responses. Recent studies indicate that ascites-derived cells may be more sensitive to drugs targeting mTOR and phosphoinositide 3-kinase (PI3K)^79^, highlighting the need to tailor treatment based on tumor location rather than diagnosis alone, particularly in patients with high tumor burden. These findings underscore compositional and potential functional differences between ascites and solid tumor tissue^51,59^, particularly when considering patient-matched samples taken at the same time. This highlights the need for clinical trials to specify a single site for diagnostic sampling and predictive biomarker assessments.

Another important finding was the high prevalence (43%) of WGA, primarily in relapsed patients. The presence of WGA was also correlated with shorter progression-free survival in newly diagnosed patients, consistent with a recent study linking genome doubling to poor prognosis in ovarian cancer^80^. Genome doubling occurs in approximately 38% of ovarian cancers, and is also common in other cancers such as microsatellite stable colorectal cancer, where it is associated with *TP53* hotspot mutations and loss-of-function^81^. In our study, we observed downregulated p53 expression in post-NACT samples from patients who had received three cycles of chemotherapy before sampling. Altered p53 expression has been observed following chemotherapy in both breast and ovarian cancer^82–85^. In esophageal adenocarcinoma, *TP53* mutations are linked to higher levels of somatic CN alterations. Furthermore, evolutionary branches marked by the acquisition of whole genome duplication demonstrated significantly higher somatic CN alterations compared to non-whole genome duplication branches. Whole genome doubling is a common event in various human cancers, resulting in genetically unstable tetraploid cells that foster chromosomal instability, thereby fueling tumor acquisition of aneuploidies^86–88^. This may suggest that loss of wildtype *TP53* marks the onset of rapid accumulation of CN heterogeneity and complexity, in contrast to the continuous accumulation of single nucleotide variations^89^. Our data suggest that chemotherapy, together with disease progression, imposes selective pressure that may accelerate genomic evolution in *TP53*-mutant tumors. The reduced p53 expression observed in post-NACT samples may further diminish any remaining p53 tumor-suppressor activity, thereby facilitating the emergence or expansion of whole-genome-doubled clones. Together, these effects could contribute to increased chromosomal instability and patient-specific divergence. Such potential selective pressure has been described in late- compared to early-stage HGSOC, where increased ploidy and altered CN signatures, specifically CN signature 4 (whole-genome-duplication–enriched) and CN signature 3 (*BRCA1/2*-related HRD), have been observed^90^. In our study, scDNA-seq revealed a decline in the predominant CN signature 1, characterized by fewer breakpoints with ≤ 2 breakpoints/chromosome arm and > 30 Mb segments. CN signature 1 has been previously associated with oncogenic RAS signaling, including *NF1* loss and *KRAS* mutations^58^. Furthermore, this signature has also been linked as a precursor inducing chromosomal instability, which is thought to arise from disrupted G2 and mitotic checkpoint controls and chromosomal missegregation^91,92^. Conversely, our analysis revealed an increase of CN signatures 4 and 7 in pre-treated samples with WGA. CN signature 4 is associated with focal amplification resulting from failures in cell cycle control, while signature 7 corresponds to CN changes linked to whole genome duplication and non-*BRCA1/2* related HRD. Our findings, in the context of the current literature, may suggest that chemotherapy, in conjunction with disease progression, drive a cascade of chromosomal instability and accumulation of aneuploidies that contributes to genomic instability. This observation is supported by a recent Pan-Cancer study, which proposes that chemotherapy exposure scars the tumor genome and introduces an evolutionary bottleneck that selects for known therapy-resistant drivers in approximately half of treated cancer patients^93^. In addition, our CyTOF data suggest that cancer cells become increasingly patient-specific as the disease progresses. Importantly, this phenotypic shift was observed exclusively in cancer cells and was independent of the sample site, indicating that the changes are intrinsic to the cancer cells themselves. Our findings suggest that the enrichment of WGA, coupled with enhanced phenotypic divergence and chemotherapy exposure in relapsed patients, alters the tumor’s entire genome architecture, fostering inter-patient heterogeneity. This observation aligns with the understanding that polyploidy enables cancer cells to accumulate genetic alterations more readily, thereby increasing the likelihood of adaptive changes^94^. Recent studies have highlighted whole genome duplication as a key evolutionary event in HGSOC, which is consistent with data showing the frequent appearance of whole genome duplication in autopsy specimens from end-stage cancer patients. In addition, CN data demonstrate that whole genome duplication contributes to the differentiation of tumor samples^95^. Moreover, while p53 inactivation has been linked to fast intratumoral heterogeneity, selective pressures on cancer cells drive consistent patterns of genome evolution across patients^96^. These reproducible patterns of genome evolution, which extend beyond specific gene mutations, could have significant therapeutic implications, particularly for patient stratification in clinical trials.

This clinical trial employed multimodal profiling using up to eleven technologies to investigate HGSOC samples collected at different disease time points. Designed as a non-interventional feasibility study, it aimed to evaluate the practicality, turnaround time, and added value of integrating multi-omics and functional profiling into clinical decision-making workflows, rather than to assess direct clinical benefit. While the descriptive nature of the analysis precludes causal inference regarding treatment efficacy, the insights gained have informed a prospective interventional follow-up trial that will systematically evaluate the clinical impact of TuPro-guided recommendations. The applied technologies—implemented here in unprecedented breadth—characterized tumor cell composition together with genetic, transcriptomic, and proteomic features, as well as functional ex vivo drug responses. While multiple data modalities were analyzed in parallel, the study does not present a single unified, unsupervised multi-omics integration model, but instead relies on coordinated, modality-aware interpretation of complementary datasets. Although technologically comprehensive, the approach did not fully capture the phosphoproteome, metabolome, or epigenome, molecular layers that could provide additional markers such as pERK (MEK inhibitor response^97,98^) or *BRCA1* promoter methylation (PARPi sensitivity^99^). Variation in resource availability and technology access across TuPro nodes may also have influenced consistency between centers, while a limited number of matched and longitudinal samples—largely due to budget constraints—reduced statistical robustness and temporal resolution. In regard to the cohort, its composition primarily represents advanced-stage, chemo-naïve HGSOC and therefore may not fully capture the immune diversity observed in early-stage or HRD-positive disease. Future interventional studies will aim to systematically compare immune phenotypes across primary and metastatic sites. This study also revealed that differences between profiling modalities mainly reflect the intrinsic biology of the sampled compartments rather than methodological artifacts. These findings emphasize the value of integrated multi-modal tumor profiling, as molecular tumor board recommendations are shaped by complementary layers of evidence that cannot be captured by a single technology alone. Future interventional studies will systematically compare immune phenotypes across primary and metastatic sites, with specific emphasis on the anatomical side of sampling. Finally, potential treatment adaptations identified here remain hypothetical, as the technologies employed are not yet CE-certified for clinical use. These constraints define the ethical boundaries of the current study but also underscore the need for further research in personalized oncology to translate biology-driven treatment decisions into certified clinical settings.

The TuPro approach allowed in-depth cancer biology assessments and facilitated personalized treatment predictions at our MTB. Our findings highlight the need for multi-omics panel testing beyond only genomics. Namely, the addition of TuPro data indicated that 76% of all study patients would benefit from a treatment change compared to SoC. Importantly, while first-line chemotherapy with carboplatin and paclitaxel was generally considered SoC and was ethically not possible to be altered within this setup, most treatment recommendations affected maintenance therapy. Our findings also suggest potential treatment alternatives for chemo-naive patients. Currently, the only targeted therapies used in the maintenance setting after chemotherapy for HGSOC are PARPi and anti-VEGF antibody therapies. Ongoing trials are also exploring the use of endocrine treatment in this context (ENGOT-ov54/Swiss-GO2/MATAO, NCT04111978).

In this project, due to ethical limitations, we restricted drug alterations to maintenance therapies following the MTB discussion. Although this was the major change made, it already delivered a first indication that this could be of survival benefit for newly diagnosed patients. Specifically, the personalized change of PARPi drug or the addition of endocrine treatment seemed to have an impact on the outcome of patients. While a deviation from current practice would require supporting evidence from a prospective randomized clinical trial, our data suggest this could indeed have significant implications for patient outcomes.

Similar shifts in treatment strategy have already been applied in breast cancer, where de-escalation trials are exploring the use of targeted therapies instead of chemotherapy at the time of diagnosis (e.g., WSG-ADAPT trial, NCT01779206). Our trial supports that (neo-) adjuvant treatment in HGSOC should be guided by the tumor’s molecular profile, potentially favoring personalized molecularly guided targeted therapies over traditional chemotherapy. While this hypothesis is supported by the extensive data generated by our analysis, we acknowledge that further evidence from interventional clinical trials is needed to validate this approach. To further investigate this, we are currently enrolling patients in the prospectively randomized clinical OV Precision Trial (Swiss-GO Trial Group/ Tumor Profiler Center, NCT06466382). This trial aims to provide early insights into the feasibility and patient outcome of multi-modal, MTB-driven treatment guidance in clinical practice for newly diagnosed ovarian cancer patients.

In conclusion, we present a unique resource of HGSOC profiles at the DNA, RNA, protein and functional levels, both at bulk and single-cell resolution. These profiles were generated and discussed within four weeks of sampling, highlighting the clinical applicability of our approach and paving the way for follow-up interventional studies.

## Methods

All research was conducted in accordance with relevant ethical regulations. The study protocol was approved by the Ethics Committee of Northwest and Central Switzerland (EKNZ, BASEC: 2018-02052) at the University Hospital Basel, and all procedures were performed in accordance with institutional and national guidelines. Written informed consent was obtained from all participants prior to enrollment. This study is registered under ClinicalTrials.gov identifier NCT06599749. The Swiss Tumor Profiler (TuPro) ovarian cancer study is a prospective, multi-center, non-interventional feasibility study. The primary objective was to evaluate the feasibility of comprehensive multimodal molecular profiling within a four-week turnaround time and its utility to inform hypothetical treatment recommendations via a molecular tumor board. Secondary objectives included characterization of cellular and molecular profiles across disease stages, ex vivo drug response landscapes, and inter- and intra-tumoral heterogeneity. The primary and secondary outcomes reported here have not been previously published; the present manuscript constitutes the primary report of trial results.

### Patient selection and data acquisition (TuPro)

For TuPro in ovarian cancer, we enrolled a total of 62 female patients between December 2018 and March 2021 in three Swiss hospitals: University Hospital Basel, Cantonal Hospital Liestal and University Hospital Zurich. Inclusion criteria encompassed patients aged 18 years or older with an ECOG performance status score of 2 or lower, and a primary or recurrent high-grade serous adenocarcinoma of ovarian, tubal, or peritoneal origin. Exclusion criteria involved serious medical, psychiatric, psychological, familial, or geographical conditions that might interfere with the project, as determined by the project leader, and legal incompetence. Sixteen patients were excluded from the project due to different tumor entities, pathology-defined exclusions based on necrosis or absence of cancer cells, and screening failure or canceled surgery. All clinicopathological data are summarized in Supplementary Table S1 and clinical parameters per sample are provided in Supplementary Data 1. Written informed consent was granted by all patients, and ethical allowance granted by the Ethics Committee Northwest and Central Switzerland (EKNZ, TuPro; BASEC: 2018-02052). All treatments during this study were solely based on the current clinical guidelines, and the decisions made by the treating physician and the patient.

All patients were diagnosed with high-grade serous ovarian carcinoma (HGSOC) based on histopathologic examination of surgically resected tissue, following WHO diagnostic criteria. Diagnosis was supported by contrast-enhanced computed tomography (CT) as the preferred imaging modality and serum CA125 as a tumor marker. Representative slides were centrally reviewed by expert gynecopathologists and characterized by solid, papillary, glandular, or cribriform growth patterns with marked nuclear atypia, pleomorphism, and high mitotic activity. Immunohistochemical staining for WT1, PAX8, and p53 was performed in most cases to confirm the diagnosis.

### Statistics and reproducibility

Due to the exploratory nature of this study, the sample size was not predetermined. As this was an observational study, no randomization or blinding was performed. Data from all patients meeting the predefined inclusion criteria were included in the analyses. No specific samples were excluded. However, not all samples were analyzed across all technologies due to limited cell numbers (see Supplementary table 1 and Supplementary Fig. 1).

### TuPro Data aggregation and molecular tumor board evaluation

The TuPro data were aggregated and summarized within a 4-week turnaround time and subsequently presented to a Molecular Tumor Board (MTB). The MTB systematically evaluated and compared the information derived from routine clinical assessment (routine pathology) to FMI targeted genomic profiling in the first step, followed by an assessment incorporating all TuPro technologies in the second step.

The MTB assessed whether genetic mutations identified by FMI or data obtained from TuPro technologies influenced hypothetical treatment decisions compared to those based solely on standard clinical and histopathological evaluations. The MTB further categorized the impact of molecular and functional data on treatment decisions using the following classifications: No evidence – no impact on clinical treatment decision support, confirmation – supports the existing clinical decision, minor change – partially modifies the treatment decision (e.g., within the same drug class or secondary drug replacement), and major change – leads to a complete shift in the treatment strategy, such as initiating chemotherapy or targeted therapies, or removing a potential treatment based on insufficient evidence (e.g., excluding PARP inhibitors in low-loss-of-heterozygosity (LOH) tumors).

To assess the influence of TuPro-derived data, the MTB followed a structured three-step process: (1) Clinical case and initial treatment decision – the standard clinical assessment and histopathological evaluation served as the baseline for decision-making. (2) Review of FMI data – the incorporation of FMI results led to a reconsideration of the treatment decision. (3) Inclusion of the full TuPro dataset – all TuPro analyses, including functional ex vivo drug testing and biomarker evaluation, were presented to reach a final hypothetical treatment recommendation. This decision was then compared to both the initial clinical decision and the FMI-based recommendation. Following each MTB session, participating clinicians were asked to evaluate the usefulness of the MTB and affiliated molecular summary report in supporting treatment recommendations. Responses were collected using a six-point Likert scale (Not useful, Barely useful, Somewhat useful, Partially useful, Largely useful, Very useful). These assessments reflected each clinician’s individual perception of the contribution of the MTB to clinical decision-making, and were not guided by additional predefined criteria.

### Sample processing and distribution

According to routine procedure, the tumor material was assessed by a pathologist who identified a region to be processed to generate FFPE specimens, which were provided to DigiPath, FMI gene panel sequencing, and IMC. Viable solid tumor material or ascites was sent to a central laboratory for immediate dissociation of samples to obtain single-cell suspensions. The tumor sample was sliced into 2 mm pieces and incubated for 1 h in the presence of collagenase, hyaluronidase at 37 °C under rotation using a gentleMACS (Miltenyi Biotec). Upon dissociation, cells were prepared for the different TuPro nodes. For CyTOF, the cell suspension was stained for viability with 25 μM cisplatin (Enzo Life Sciences) in a 1 min pulse before quenching with 10% FBS. Cells were then fixed with 1.6% paraformaldehyde (Electron Microscopy Sciences) for 10 min at room temperature. For Proteotyping, cells were snap frozen in liquid nitrogen. For scRNA-/scDNA-seq, Pharmacoscopy, and 4i DRP, cells were viably frozen in RPMI + 20% FBS + 10% DMSO. Samples were kept at -80 °C until shipment.

### Digital pathology

One whole-section slide of each HGSOC was stained with antibodies against CD4, CD8, CD68, PD-1 and PD-L1. Immunohistochemical slides as well as one H&E stained slide were then scanned with the VENTANA DP200 scanner (Roche Diagnostics International AG, Rotkreuz, Switzerland) and analyzed digitally. FFPE tumor blocks were cut at a 4 μm thickness for immunohistochemistry staining. The sections were pre-treated with CC1 and subsequently incubated with prediluted antibodies (all from Ventana, Roche Diagnostics International AG) against CD4 (clone SP35, 4 min), CD8 (clone SP57, 4 min), ARID1A (poly, 1:100), p53 (clone DO-7, RTU), PR (clone 1E2, RTU), PAX8 (clone EP331, RTU), Napsin A (clone MRQ-60,RTU), CD20 (clone L26, RTU), CK22 (polyclonal, 1:2000), WT-1 (clone 6F-H2, RTU), ER (clone SP1, RTU), CD3 (clone 2GV6, RTU), FOXP3 (clone SP97, 1 :100), PD-1 (clone NAT105, 12 min) and PD-L1(clone SP263, 32 min) as well as CD68 (Dako clone IR613, 16 minutes). DAB was used as a chromogen, and counterstaining was performed with Hematoxylin (Roche Diagnostics International AG). All analyses were performed on the BenchMark XT automated immunostainer (Roche Diagnostics International AG) using the OptiView detection system (Ventana Medical System Inc., Roche Diagnostics International AG).

Assessment of digital slides in Basel was carried out by three expert pathologists (JB, MMB and SM), assessment of research slides in Zurich was carried out by BS and VHK. Tumors were defined as immune-excluded, immune-desert or immune-inflamed based on the definition by Chen and Mellmann^100^. For PD-1, the percentage of positive immune cells was assessed. For PD-L1, the percentage of positive tumor and immune cells was recorded.

### Targeted next generation sequencing

Mutational profiling was performed using the FoundationOne®CDx assay (FMI, Roche, Switzerland)^101^, which includes 324 cancer-related genes for the detection of base exchanges, insertions, deletions, and CN changes. In addition, specimens are simultaneously profiled for LOH, tumor mutational burden and microsatellite instability (see Foundation Medicine). The tumors were analyzed in the Foundation Medicine laboratory at the Department of Pathology and Molecular Pathology at the University Hospital Zurich as previously described. All cases included in this study were evaluated by an experienced board-certified pathologist at the time of specimen arrival in the laboratory and then reviewed by a single pathologist to confirm the diagnosis. DNA was extracted from FFPE tissue blocks with at least 20% tumor content with the Maxwell 16 FFPE Plus LEV DNA Purification Kit (AS1135, Promega, Dübendorf, Switzerland) according to the recommendations of the manufacturer. The DNA stock concentration was between 50 and 100 ng/µL for further analysis. Samples were assayed by adapter ligation hybrid capture, performed for all coding exons of 309 cancer-related genes plus select introns from 34 genes. Sequencing was performed using the Illumina HiSeq instrument (Illumina, Zurich, Switzerland) to a median exon coverage ≥ 500 x, and data were analyzed for all classes of genomic alterations. The computational pipeline used to analyze sequence patterns used Bayesian algorithms to identify base substitution mutations, local assembly to identify short insertions and deletions, comparisons with process-matched normal controls to determine gene amplifications and homozygous deletions and the analysis of chimeric read pairs to identify gene rearrangements and gene fusions^101^. Using 0.8 to 1.1 Mb of sequenced DNA for each case, the tumor mutational burden was determined using the number of somatic base substitution or indel alterations per Mb after filtering to remove germline and pathogenic mutations. Microsatellite instability (MSI) was defined as stable vs unstable as defined in the test. Loss of heterozygosity (LOH) was defined as the percentage of gene copy loss. The reporting of mutations / amplifications / copy loss / re-arrangements / truncations were specifically reported to each sample. In this manuscript, the attribution of the definition of “targetable” to any single gene or genomic pathways is based on the current knowledge of available therapeutic compounds already approved in any tumor types. Such a definition relied on an informal consensus among authors, based on the review of existing approved testing and therapy combinations, similar to what has been previously reported by the American Society of Clinical Oncology (ASCO) and the European Society for Medical Oncology (ESMO), added to emerging clinical trial data.

### RNA-sequencing (RNA-seq, bulk transcriptomics)

#### Sequencing, alignment and counting

Total RNA was isolated from cell suspensions using the Quick-RNA Microprep Kit (Zymo Research Corp, Irvine, USA), including DNase I treatment according to the manufacturer’s instructions. The quality of the isolated RNA was checked with a 4200 TapeStation device (Agilent Technologies Schweiz AG, Basel, Switzerland). RNA-seq libraries were prepared using TruSeq Stranded Total RNA Library Prep Gold reagent kits (Illumina, Zurich, Switzerland) according to Illumina’s guidelines. QC of sequencing libraries was performed using Fragment Analyzer systems (Advanced Analytical, AATI, Ankeny, USA). Libraries were pooled and sequenced PE100 on an Illumina NovaSeq 6000 system (Illumina, Zurich, Switzerland). Alignment to the reference genome (GRCh38) was performed with STAR^102^. The raw expression counts were obtained using simple counting, an in-house pipeline accessible through GitHub:gromics.

#### Sample quality control

Quality control (QC) of samples was performed based on their RNA Integrity Score (RIN) and the following FASTQC metrics: “Per Sequence GC Content”, “Overexpressed Sequences”, “Per Base Sequence Quality”, “Per Sequence Quality Scores”, and “Per Base N Content”. If multiple FASTQ files were generated per sample, e.g., due to sequencing separately forward and reverse reads or sequencing in multiple lanes or flowcells, the FASTQC metrics were aggregated per sample, with at least one FAIL/WARN resulting in FAIL/WARN for this metric for the considered sample. In order to determine the overall sample QC, taking into account all listed FASTQC metrics as well as RIN score, the following criteria were adopted: RIN below six and three or more failed FASTQC modules resulted in classifying a sample as FAIL, RIN score above 7 and less than two failed FASTQC modules classified samples as PASS, anything in-between or with missing information categorized sample as WARN. Applying these criteria to the cohort resulted in: 3 x FAIL, 11 x WARN and 38 x PASS, with both WARN and PASS samples being considered for preprocessing and all downstream analyses.

#### Data preprocessing

Only protein coding genes, according to Gencode release v32 (GRCh38.p13) were kept, with the exception of mitochondrial genes, and further filtered to a set of genes with more or equal to 10 counts in the cohort (sum across all samples). For pathway analysis, three subsequent preprocessing steps were taken: a variance stabilizing transformation (VST), batch-effect correction and regressing out effects of confounding variables. VST, which internally adjusts for differences in library sizes between samples, was performed with DESeq2^103^, without taking any design information into account. Moreover, batch correction using removeBatchEffect from the limma package^104^ was carried out using a sequencing date as a batch (with a total of 6 batches). Furthermore, the first principal component capturing differences in RIN score (Pearson’s r: − 0.68), as well as tumor content (estimated using CyTOF) were regressed out using the removeBatchEffect function.

#### Analysis

##### Differential pathway activation analysis

Pathway activation scores were computed using the GeneSet Variation Analysis (GSVA^105^) with the minimum GeneSet size per pathway set to five, with a Gaussian kernel, and using the normalized enrichment score (mx.diff = TRUE). A linear regression model is used to test for differential pathway activation between groups of interest. Any group with less than three five samples is excluded from testing. We perform the pairwise tests between all remaining groups, for each pathway group, e.g., Hallmark pathways or Tumor pathways, independently. All *p*-values are corrected for multiple testing using Benjamini-Hochberg per pathway category, and the pathways with FDR < 0.2 are considered differentially activated between the respective groups.

##### Subtyping

The GeneSets (“pathways”) defining the HGSOC subtypes differentiated, immunoreactive, mesenchymal, and proliferative (both UP and DOWN regulated) were obtained from literature^48^. The calculated pathway activation scores were z-scored across the cohort. Assignment to a group was performed using hierarchical clustering on pathway activations using Euclidean distance and the Ward linkage method. For clustering, *k* = 4 was pre-set on the samples, matching the number of original subtypes, and the number of clusters on the features was selected using the dynamicTreeCut algorithm^106^.

##### quanTIseq-based deconvolution

The count matrix was normalized to transcripts per million (TPM) using gene lengths from Ensembl Genes 110 in R (v 4.2.3), and then performed deconvolution with immune deconvolution v 2.1 using the Quantiseq method^107^. To calculate TPM, raw gene expression counts were normalized to reads per kilobase by dividing each count by its corresponding gene length in kilobases. The reads per kilobase values were then scaled and normalized by the sum across all genes within a sample to ensure that the sum of TPM across each sample equaled one million. The TPM calculation is given by the formula:

1 $${TPM}={{\rm{t}}}\left(\frac{{{\rm{t}}}\left(\frac{{{\rm{counts}}}.{{\rm{mat}}}}{{{\rm{gene}}}.{{\rm{length}}}}\right)^{\!.10^{6}}}{{{\rm{colSums}}}\left(\frac{{{\rm{counts}}}.{{\rm{mat}}}}{{{\rm{gene}}}.{{\rm{length}}}}\right)}\right).$$

### Proteotyping (bulk proteomics)

#### Experimental

Samples for proteotyping were generated using the PreOmics iST kit (PreOmics GmbH, Martinsried, Germany). Cells were lysed, digested and cleaned up according to the manufacturer’s recommendations. Lysis was supported by an additional sonication step using three 30 s sonication pulses in a VialTweeter (Dr. Hielscher, Tottweil, Germany). Samples were resuspended at a concentration of 1 μg/μl for MS measurements. Samples were analyzed on an Orbitrap Lumos mass spectrometer or alternatively on a Q Exactive HF-X mass spectrometer (Thermo Fisher Scientific, Reinach, Switzerland) equipped with an Easy-nLC 1200 (Thermo Fisher Scientific, Reinach, Switzerland). Peptides were separated on a C18 50 cm EASY-Spray™ HPLC column (2 µm, 100 Å, 75 µm i.d.(ES903, Thermo Fisher Scientific, Reinach, Switzerland). Mobile phase A consisted of HPLC-grade water with 0.1% formic acid, and mobile phase B consisted of HPLC-grade ACN (80%) with HPLC-grade water and 0.1% (v/v) formic acid. Peptides were eluted at a flow rate of 200 nl/min using a non-linear gradient from 4% to 52% mobile phase B in 117 min. For data-independent acquisition (DIA) on the HF-X, DIA isolation windows were set to 15 m/z and a mass range of m/z 400-1210 was covered. A total of 54 DIA scan windows were recorded at a resolution of 30,000 with an AGC target value set to 1e6^30,108^. For DIA on the Lumos, DIA isolation windows were variable, and a mass range of m/z 350-1650 was covered. Resolution was set to 30,000 with a normalized AGC target of 2000%. HCD fragmentation was set to 24%, 27%, 30% stepped (Lumos) or 28% normalized (HF-X) collision. Full MS spectra were recorded at a resolution of 120,000 with a normalized AGC target of 250% and a maximum injection time of 60 ms (Lumos) or an AGC target of 3e6 and a maximum injection time of 50 ms (HF-X).

#### Analysis

TuPro DIA data were analyzed using Spectronaut v13 (Biognosys, Schlieren, Switzerland). For the TuPro cohort, MS1 values were used for peptide quantification, peptide quantity was set to sum. Data were filtered using Qvalue with a precursor Qvalue cut-off of 0.001 and a protein Qvalue cut-off of 0.01 FDR. Interference correction was performed. Quantitation was set to Qvalue identified, missing values were imputed with the missing values derived from the experiment-wide distribution of quantities. Data were searched against a Uniprot human library (release April 2018) together with standards and common contaminants in standard DIA with a sample-specific spectral library generated in house using ph-fractionated samples from OVCAR3, SKOV3 and CD3, CD14 and CD138 pooled and sorted primary cells. Batch correction on the Spectronaut peptide output was performed using proBatch^109^.

### Cell-free DNA-sequencing

Blood samples were collected using Cell-Free DNA BCT™ (Streck, La Vista, USA). The plasma fraction was separated from the blood cells by two consecutive rounds of centrifugation for 30 minutes at room temperature at 1600 x *g*. The collected plasma was aliquoted and stored at − 80 °C until use. CtDNA was extracted using the MagMax Cell-Free Total Nucleic Acid Isolation Kit (Thermo Fisher Scientific, Waltham, USA) according to the manufacturer’s instructions. The extracted ctDNA was quantified using the dsDNA HS assay kit by the Qubit 4.0 Fluorometer (Thermo Fisher Scientific) and Tapestation (Agilent Technologies Schweiz AG, Basel, Switzerland). NGS libraries were prepared using a custom Ampliseq™ HD panel, covering cancer-hotspot regions in 13 selected genes (*AKT1, CCND1, CDK4, EGFR, ERBB2, ERBB3, ESR1, FGFR1, HRAS, KRAS, MET, PIK3CA*, and *SF3B1*) and full coding region for 25 genes (*ARID1A, MAP2K4, ATM, MAP3K1, BRCA1, NF1, BRCA2, PALB2, CBFB, PIK3R1, CDH1, PTEN, CDKN1B, RB1, CDKN2A, RUNX1, CDKN2B, TBX3, CTCF, TP53, FBXW7, TSC1, FOXA1, TSC2*, and *GATA3*). The panel covers a total of 0.114 Mb and contains 1672 amplicons. NGS libraries were prepared using 30 ng of input ctDNA and following an adapted version of the Ampliseq™ HD general protocol for 2-pools panels. Libraries were quantified by Tapestation (Agilent Technologies) and, after appropriate dilution, loaded on Ion Chef™ and Ion Genestudio S5™ instruments for templating and sequencing (Thermo Fisher Scientific). Quality control of run and sample metrics was performed on Ion Torrent Suite™ Software v5.10 or later.

#### Analysis

Variant calling and reporting was performed using a custom pipeline based on the Ion Reporter Analysis Software v5.10 or later and the Oncomine™ Reporter v4.5.3.r.2.bed58a (Thermo Fisher Scientific, Reinach, Switzerland). Common SNPs were filtered by cross-referencing with UCSC Common SNPs^110^ (PMID: 11125122), ExAC 1000 Genomes^111^ (PMID: 26432245). Somatic variants in homopolymer stretches longer than 8 bp were also excluded. Our minimum quality control criteria for sensitivity was set as the limit of detection (LOD) of the 50th percentile of amplicons higher than 3%. Variants detected in a minimum of 3 independent molecules, but with a molecular frequency below the local limit of detection or below the limit of detection reached at validation (0.7%), are deemed ‘low confidence variants’. The molecular frequency^112^ of pathogenic mutations was used as a proxy for ctDNA fraction. When multiple pathogenic mutations were detected, the driver mutation with known tissue status or the highest molecular frequency was chosen for ctDNA fraction approximation.

### Single-cell RNA sequencing

#### Experimental

Viability and cell count of the single-cell suspensions were assessed with a Cellometer K2 (Nexcelom Bioscience, Lawrence, USA). Single cells were captured with the Chromium Controller (10X Genomics, Pleasanton, USA), and scRNA-seq libraries were prepared with the Chromium Single Cell 3’ Library & Gel Bead Kit (10x Genomics) according to the manufacturer’s instructions. QC assessment of cDNAs and libraries were performed with Fragment Analyzers (Advanced Analytical, AATI, Ankeny, USA). Libraries were sequenced with Illumina NextSeq 500 and NovaSeq 6000 systems (Illumina, Zurich, Switzerland) according to the manufacturer’s guidelines.

#### Analysis

In total, 55 HGSOC samples were investigated for the cohort-level analyses. The basic processing of individual samples was performed using the scAmpi pipeline previously described^36^. This includes read mapping with Cellranger (version 3.1.0, 10x Genomics) against the GRCh38 reference with annotation Ensembl 93, quality control and filtering, count normalization, unsupervised clustering, as well as cell type classification and GeneSet analysis. The quality control and filter steps removed doublets identified by scDblFinder^113^, excluded non-protein coding genes and reads mapping to ribosomal RNA and mitochondrial genes, and excluded cells with less than 400 different transcripts or more than 50% of their reads mapping to mitochondrial genes. For the cell type classification of ovarian cancer samples, we applied the scROSHI method^114^ on cell type signatures derived from learning markers on a test cohort of ovarian cancer cell lines and patient samples, together with expert curated signatures from the literature^115^.

Differential expression analysis and GeneSet comparisons between groups of samples were performed on pseudo bulk data^116^. To this end, in each sample, raw counts were summed up per gene over all cells of a given cell type. The characteristics of the resulting data are similar to bkRNAseq count matrices and were analyzed according to the standard DESeq2 workflow^103^. A minimum threshold of at least 20 cells per cell type population and sample was required to perform the pseudo bulk analysis.

### Single-cell DNA-sequencing

#### Experimental

Viability and cell count of the single-cell suspensions were assessed with a Cellometer K2 (Nexcelom Bioscience, Lawrence, USA). Single cells were captured with the Chromium Controller, and scDNA-seq libraries were prepared with the Chromium Single Cell DNA Library & Gel Bead Kit (10x Genomics) according to the manufacturer’s instructions. QC assessment of sequencing libraries was performed with Fragment Analyzers (Advanced Analytical, AATI, Ankeny, USA). Libraries were sequenced with Illumina’s NextSeq 500 and NovaSeq 6000 systems according to the manufacturer’s guidelines.

#### Analysis

In total, 51 samples derived from 50 HGSOC patients were analyzed. From the sequencing data of each sample, after the barcoded reads are mapped, they are demultiplexed to assign them to individual cells. With the Cell Ranger software, the reads are binned into 20 kb genomic regions and corrected for GC content and mappability. The adjusted number of reads in each bin are then reflective of the underlying CN state for that cell in that genomic region.

Before identifying CNAs, we detect potential breakpoints by looking for differences in read counts in adjacent genomic regions by combining signals across all cells. Then, from the segmented genome, we cluster cells into clones and infer their phylogenetic relationships and hence their CN profiles using a version of the SCICoNE software^39^. Malignant cells are defined as those distinct from the root neutral state in at least 1% of the genome. To call WGAs, we run the inference twice, once with a diploid root and once with a tetraploid root. The latter allows for finer changes in CN state, and the classification is based on a visual inspection of the two analyses per sample. For the CN signature quantification, we evaluated the strength of the seven signatures described in ref. ^58^. These signatures were derived from the mixture modeling of the genome-wide distributions of six hallmark CN features obtained for HGSOC using shallow bulk whole-genome sequencing and targeted amplicon sequencing of *TP53* in the BriTROC-1 cohort, and reduced to seven signatures using non-negative matrix factorization^58^. The signature was evaluated for each clone in our cohort, while the signatures for each patient sample were computed as the average over their malignant sample clones weighted by the clone size.

### Single-cell mass cytometry (CyTOF)

#### Experimental

##### Antibody panel

Two antibody panels were developed for the needs of the HGSOC cohort analysis (Supplementary Data 14). Antibody conjugation was performed using the MaxPAR antibody conjugation kit (Standard BioTools, South San Francisco, USA) according to the manufacturer’s instructions. The concentration was adjusted to 200 mg/mL in Antibody Stabilizer (Candor GmbH, Wangen, Germany), and the optimal concentration was determined upon titration between 0.25 and 4 µg/mL. All antibodies used in this trial were managed using the cloud-based platform AirLab^117^. To ensure data consistency over the course of the trial, a master mix was created and frozen as 4x aliquots. The effect of freezing on antibody performance was assessed by comparing data obtained upon staining of reference cells with fresh vs frozen panels^118^.

PBMC and tumor cells from up to 5 patients were barcoded using a 3 chosen 6 palladium only barcoding scheme (102 Pd, 104 Pd, 105 Pd, 106 Pd, 108 Pd, and 110 Pd, Standard BioTools). Upon barcoding, patient sample cells were mixed with pre-barcoded reference cells, which were used to perform batch correction, as described previously^118^. Combined cells were split and incubated with FcR blocking reagent (Miltenyi Biotec, Adliswil, Switzerland) for 10 min at 4 °C before staining for 30 minutes at 4 °C with either the tumor or the immune antibody panels. Before acquisition on CyTOF (Helios system, Standard BioTools), the cells were resuspended in 1 mL of nucleic acid Ir-Intercalator in 1.6% PFA/PBS for 1 h at room temperature (Standard BioTools). Data was acquired on a Helios system (Standard BioTools) in multiple FCS files.

#### Analysis

CyTOF data were pre-processed for bead based signal normalization, signal spillover correction, single-cell de-barcoding, and batch correction as previously described^119^. During the course of the trials, two different versions of the immune and the tumor panel were used. Samples analyzed in phase one for which enough material was available were reanalyzed with the final version of the panel. Only samples assessed with the final version of the panel were included in the cohort analysis described in this manuscript. For the tumor panel, this corresponded to a total of 76 samples (Ascites = 15, Omentum = 35, Ovary = 15, Lymph node = 5, Peritoneum = 3, Relapse = 2, Breast = 1). For the immune panel, data from 62 samples were available (Ascites = 12, Omentum = 27, Ovary = 14, Lymph node = 4, Peritoneum = 3, Relapse = 1, Breast = 1). The main cell types, including fibroblast, tumor, immune, endothelial, and non defined cells, were identified using a random forest classifier trained on manually gated cell populations in Cytobank. The training was performed on 1700 to 40,000 cells, depending on the cell types, using the 25 common markers across all samples and 500 decision trees. Cells with a probability score below 0.6 or with a difference with the second- highest probability lower than 0.3 were identified as unassigned (median freq = 0.038). At each step of the analysis (all cells versus tumor cells), dimensionality reduction was performed using the *t*-SNE algorithm, with the default parameters on a maximum of 600 cells per sample. The same approach was applied to the immune data collected from the tumor and the PBMC samples. Tumor cell clusters across treatment types (naive, NACT, relapse) were identified using PhenoGraph.

#### Tumor heterogeneity assessment

##### Derivation of tumor phenotypes

To identify tumor phenotypes, we use the expression profiles based on the tumor panel. To avoid cell-count bias, we subsample the data to a maximum of 500 cells per sample. We cluster the tumor cells using the phenograph method^120^ with the Louvain community detection^121^. We use an R implementation of the described algorithm (Rphenograph, https://github.com/JinmiaoChenLab/Rphenograph) with *k* = 50 and otherwise default settings.

##### Intra-tumor heterogeneity assessment

We assess the intra-tumor heterogeneity, or phenotypic complexity, by calculating the heterogeneity index HTI^122^, a variation of the Shannon entropy. To ensure that the heterogeneity index HTI is bounded within the [0, 1] range, the logarithm base is set to the number of clusters. More formally, the heterogeneity index for a sample is defined as

2 $${HTI}=-{\sum }_{c=1}^{C}\,{p}_{c}{lo}{g}_{C}({p}_{c})$$

with C denoting the number of clusters and ${p}_{c}$ the proportion of cells from a given sample in cluster c. Under uniformity and equal-size assumptions, HTI is invariant to the number of clusters.

##### Inter-sample heterogeneity assessment

To investigate inter-sample heterogeneity, we first calculate the divergence of the tumor’s phenotypic profile between each given sample and the studied cohort. To this end, we employ the Jensen--Shannon (JS) divergence^123^, which is a symmetric version of Kullback--Leibler (KL) divergence^124^ that measures the difference between two probability distributions.

For two discrete probability distributions P and Q, JS divergence is defined as

3 $${JS}\,(P{{\rm{||}}}Q)=\frac{1}{2}{D}_{{KL}}\left(P {{\rm{||}}} \frac{P+Q}{2}\right)+\frac{1}{2}{D}_{{KL}}\left(Q{{\rm{||}}}\frac{P+Q}{2}\right)$$

where ${D}_{{KL}}$ denotes the Kullback-Leibler divergence. The KL divergence is defined as

4 $${D}_{{KL}}(P{{\rm{||}}}Q)={\sum}_{x\in \chi }\,P(x)\log \left(\frac{P(x)}{Q(x)}\right)$$

where χ denotes the sample space.

##### Comparison of heterogeneity scores between groups

In order to formally compare heterogeneity scores between clinically defined groups, we employ the class of linear mixed models. This was particularly possible due to the existence of multiple samples per patient for 14 (out of 44) patients, including three longitudinally profiled patients with both chemo-naive and relapse samples. To capture the potential intra-patient sample similarity, we use a random intercept per patient, which induces a block-diagonal covariance structure.

##### Data visualization with alluvial plots

In order to visually represent tumor heterogeneity within and across the samples, we used an alluvial plot. The visualization is performed for chemo-naive and relapse groups in separate blocks. The strata on the first horizontal axis or level (left) represent samples, and the strata on the second horizontal axis (right) correspond to tumor cell clusters, also referred to as phenotypes. The alluvia links between the axes depict the number of cells from a given sample that belong to a certain tumor cluster. To reduce noise in the visualization, we drop connections resulting from less than five cells. Alluvia are colored by patient ID, the objects corresponding to samples are colored by metadata, and, in the case of cancer cells, the objects corresponding to clusters by cluster heterogeneity. The cluster heterogeneity is defined based on the proportion (p) of samples whose cells are present in a given cluster, with the denominator corresponding to the number of samples in a given block, i.e., in the chemo-naive or relapse group. We then use the proportions to label the clusters as low p≤0.1, medium-low $p\in ({\mathrm{0.1,0.2}}]$, medium-high $p\in ({\mathrm{0.2,0.3}}]$ and high p≥0.3 heterogeneity.

### Spatial imaging mass cytometry (IMC)

#### Experimental

##### Slide Preparation and Staining

Using the antibody panel described in Supplementary Data 14. Tissue slides were stained in the following manner. Slides were deparaffinized using the AS-2 Automatic staining system (Pathisto GmbH) with the following program: 3 × 10 min Ultraclear, 2 × 5 min 100% EtOH, 2 × 3 min 96% EtOH, 2 × 3 min 90% EtOH, 1 × 3 min 80% EtOH, 1 × 3 min 70% EtOH, and Tris-buffered saline (TBS) for 5 min. Antigen retrieval was conducted with Tris-EDTA (pH 9) buffer at 95 °C in a NxGen decloaking chamber (Biocare Medical) for 30 min. Following cooling, slides were blocked in 3% BSA in TBS with 0.1% Tween for 1 h at room temperature. Samples were stained overnight at 4 °C with the antibody panel mixed in a blocking buffer. Samples were then stained with iridium (500 µM 1:100 in TBS) for 7 min, followed by three 5 min washes with TBS. Samples were shortly rinsed (3 sec) with double distilled H_2_O (ddH_2_O) and dried with compressed air.

##### Imaging mass cytometry

The IMC data collection was performed using a Hyperion Imaging System from Standard BioTools, operating at a laser frequency of 200 Hz. Raw image data were transformed into TIFF format via the ImcSegmentationPipeline (https://zenodo.org/record/3841961#.Y1vp5sFByFo). CellProfiler was utilized to perform cell segmentation on predicted nuclei and cytoplasmic compartments generated by a customized U-Net model^125,126^. In addition, CellProfiler was used to extract cell marker intensities following spillover compensation.

#### Analysis

IMC data were processed for signal spillover using CATALYST^119^, and cell counts were transformed using the inverse hyperbolic sine function. The main cell types, including tumor, vessels, and immune, were first identified using Leiden clustering on a subset of 1000 cells per sample, followed by a random forest trained on the annotated clusters. The Random forest model OOB estimate of error rate was 3.63% and was used to predict all cell types on the whole dataset. The same strategy was used on immune cells to identify B cells, CD8 and CD4 T cells, granulocytes, macrophages, NK cells and plasma cells. The random forest model OOB estimate of error was 2.93% on the immune cell data subset and was used to infer immune cell types on the rest of the immune data.

### Pharmacoscopy

#### Experimental

Pharmacoscopy was performed as previously described^42,43,127^. The single cell suspension prepared in the central lab was diluted to around 0.1 million cells / ml (corresponds to 2500 cells/well) and seeded into wells of a 384-well plate containing drugs and drug combinations (Supplementary Data 10) as well as DMSO controls in each well. If there were not enough cells to fill a whole plate, a smaller drug panel including only high-priority drugs as defined by the clinical team and omitting redundancy in drug targets was tested. Cells were incubated at 37 °C, 5% CO_2_ overnight, then the media was aspirated, and cells were fixed with 10% formaldehyde solution in PBS (37% formaldehyde solution, Sigma-Aldrich F8775-500ML) corresponding to 3.7% final concentration of formaldehyde in the fixative. The fixative was aspirated, and cells were permeabilized with 0.01% Triton-X in PBS containing 1% bovine serum albumin (BSA, Sigma-Aldrich). In the same step, nuclei were stained with 4′,6-diamidino-2-phenylindole (DAPI, BioLegend, San Diego, USA). Subsequently, cells were stained with fluorescent antibodies against PAX8 (Abcam #ab215953), EpCAM (BioLegend #368520), E-cadherin (BioLegend #324212), phospho-H2Ax (BD #560445), Vimentin (BioLegend #677809) and CD45 (BioLegend #368520) (Supplementary Data 10 and Supplementary Data 14). All samples were imaged on an automated spinning-disk confocal microscope (PerkinElmer Opera Phenix, Waltham, USA), using 10x magnification and 9 images per well to cover the entire well area. We used five channels with non-overlapping excitation/emission filters to image the following features: Channel 1 (transmission / 650–760 nm) for brightfield to capture general cell shape and texture, channel 2 (405 nm / 435–480 nm) for DAPI / nuclei, channel 3 (488 nm / 500–550 nm) for vimentin or pH2Ax, channel 4 (561 nm / 570–630 nm) for immune cells (CD45), and channel 5 (640 nm / 650–760 nm) for a combination of all HGSOC markers (EpCAM, E-cadherin, and PAX8).

#### Analysis

##### Data preprocessing and cleanup

Single-cell detection in the raw images was performed using CellProfiler v2.2.0^128^. Individual cells were detected based on maximum correlation thresholding of the DAPI signal. The exact parameters of the pipeline were adjusted per sample to account for differences in nucleus intensity, size and the presence of large spheroids. Staining intensities were extracted for the nucleus, and a region of 6 pixels around the nucleus that was used as a proxy for cytoplasmic intensity. For downstream analysis, intensities were log10 transformed and corrected for variation in the local background as previously described^127^. Cells with very low DAPI intensities or abnormally high or low nucleus areas likely represent segmentation artifacts and were therefore removed from the analysis by manual gating. In addition, outlier wells (very low or high total cell numbers, or aberrant staining patterns) were removed if the observed patterns could be attributed to pipetting mistakes or the presence of large cell clumps by visual inspection.

##### Cell type classification by immunofluorescence gating

To generate drug response scores for individual samples within the 4 weeks turnaround time from sampling to MTB discussion, ovarian cancer cells were identified by thresholding the immunofluorescence signal. A cell that stained positive for at least one of the cancer markers (Pax8, EpCAM, E-Cadherin) was considered a cancer cell. The drug responses shown in Fig. 2F are based on this type of cell classification.

##### Cell type classification using convolutional neural networks

HGSOC cells were identified from fluorescence microscopy images using convolutional neural networks (CNN). Due to differences in staining panels between trial phases (Supplementary Data 10), we implemented two different CNNs that were trained on a total of 10’335 (phase 1) and 87’950 (phase 2) manually labeled crops (50 × 50 pixels) centered on the nuclei of individual cells. Cells for training were randomly sampled from control (DMSO-containing) wells, and cells from all patients. We used a customized architecture based on ResNet^129^ as previously described^44,130^ that was implemented using the Deep Learning Toolbox in Matlab R2021a (MathWorks, Natick, USA). Subsequently, 75% of the images were used for training and 25% for CNN performance evaluation. The CNNs were trained to distinguish between immune cells (CD45 positive), HGSOC cells (Phase 1: EpCAM or E-cadherin positive; Phase 2: PAX8 or EpCAM or E-cadherin positive) and other cells (when markers were negative). We achieved an average test accuracy of 0.99 (Phase 1) and 0.91 (Phase 2). For classification, each cell was assigned to its most likely cell type based on the CNN class probabilities. Cells that could not be unambiguously classified (maximum class probability of < 0.6) were labeled as “unassigned”.

##### Samples analyzed and quality control

In total, 80 samples were processed and analyzed. Of those, 69 were kept for the final cohort analysis, with exclusion criteria as follows: failed experiment due to insufficient cell viability or presence of autofluorescent debris (2), less than 2% tumor content (6), or technical replication for reproducibility test (3).

##### Drug response calculation

Drug response scores were calculated per well as 1-fraction HGSOC cells in treatment/fraction HGSOC cells in control. Then, all response values were aggregated per condition (drug and concentration pair), and finally, responses were aggregated into a “PCY score” across concentrations per drug. Thus, positive values represent on-target cytotoxicity. The drug response scores were then corrected for systematic effects arising from different abundances of cancer cells. To this end, a scaling factor was calculated by regressing the interquartile range of each sample across all drugs against the tumor content. The normalization factor per sample corresponds to the fitted value of the IQR/mean(fitted_IQR). To avoid overcorrecting extreme values, the normalization factor was capped to its 0.1 and 0.9 quantile, respectively.

##### Clustering of drug response profiles

To group patients based on their drug response profiles, we used spectral clustering. Pairwise distances were calculated using a nan-robust cosine distance, which takes into account only shared observations between two vectors and returns a distance of 2 if there is no single shared observation. Spectral clustering was performed using the spectralcluster function in MATLAB R2021b (MathWorks Inc.) with a predefined number of 3 clusters.

### Iterative indirect immunofluorescence imaging drug response profiling

#### Experimental

Prior to cell seeding, 384-well plate were coated for 2 h at room temperature with a coating mix (1:1 Poly-L-Lysine (Sigma Aldrich, P4832-50ML), 1:100 Fibronectin in PBS (Sigma Aldrich, F0895-2MG). The coating mix was removed using an EL406 Washer-Dispense (WD), and the 384-well plate was left 1 h at RT to dry. Depending on the abundance of live cells in the patient samples, 3500–7500 cells were seeded per well of a 384-well plate using the WD’s peripump casette in culture medium (RPMI without L-Glutamine (Sigma Aldrich, R0883), 10% FCS (Sigma Aldrich), 1 mM Sodium Pyruvate (Thermo Fisher Scientific, 11360070), 2 mM L-Glutamine (Sigma Aldrich, G7513), Anti/Anti 100x (Gibco, 15240-096). The cells were incubated overnight at 37 °C and 5% CO2. The next day, drugs were added using a BRAVO liquid handling robot to the seeded cells at a final concentration of 5 µM and 0.5% DMSO. Vehicle-treated control cells were only incubated with 0.5% DMSO. The drug-treated cells were incubated at 37 °C and 5% CO2 for a further 8 h. After elapsing of the incubation time, the cells were fixed at 4% paraformaldehyde in PBS for 15 min. Afterwards, they were washed 4 times with PBS and permeabilized at 0.5% Triton X-100 in PBS for 15 min. Next, the cells were washed 4 times with double-distilled water (ddH2O). Iterative indirect immunofluorescence imaging^47,131^ was performed on the samples to acquire the following molecular markers: proliferating cell nuclear antigen (PCNA), phospho-ERK, phospho-AKT, phospho- ribosomal protein S6 kinase 1 (pS6K1), cleaved caspase 3 (Cl.CASP3), and phosph- mesenchymal-epithelial transition factor (Supplementary Data 14). Sample blocking, primary and secondary incubation was performed for 1 h. The nucleus of cells was stained using DAPI (every cycle). The cell out staining was performed as described in ref. ^132^. Imaging was performed using a Cytiva IN Cell 6000 at 20X magnification with a Nikon Plan Apo (0.95 NA), correction collar 0.11-0.23 CFI/Lambda objective. A total of 16 fields of view were imaged per well, with 3 z-sections per field. The Z-sections were collapsed to one image by Maximum Intensity Projection. Laser lines were used for the trial, namely 406, 488, 568, 625 nm.

#### Analysis

##### Image processing and data cleanup

Image processing and image analysis tasks on the 4i images were performed using TissueMaps (https://github.com/pelkmanslab/TissueMAPS). The tasks include image alignment of 4i cycles based on the DAPI signal, correction of the illumination bias, nuclear and cell segmentation, measurements of nuclear and cell morphology, as well as measurements of intensity moments of the different fluorescence signals in nucleus and cell objects. Using semi-supervised random forest classifiers, wrongly segmented objects or objects containing artifacts caused by sample preparation were excluded. In the next step, we excluded cells which originated from various categories of control wells (i.e., no-primary antibody control on patient cells, reference cell lines, no-primary antibody control reference cell lines) as well as cells originating from wells containing less than 100 cells. In rare cases, we detected so-called ghost cells in the datasets, which are cells which have a clear nucleus, but lack signals from the total protein or the 4i stains. These cells were excluded, based on a combination of features: area ratio (cellular area/ nuclear area), and the summed intensity of succinimidyl ester within cell segmentation mask (details on the procedure and whether it had to be used can be found in each individual upload script). In a further clean-up step, we z-normalized the nuclear DAPI mean intensity of cells for each cell separately and excluded cells for which their normalized DAPI signal exceeded 5.

##### Patient profile generation

Single-cell data exclusively containing the mean intensity readouts of the 4i antibodies (nuclear PCNA, cell-pAKT, cell-cleaved Caspase 3, cell-pS6K1, cell-pMET, cell-pERK) was normalized to the DMSO control using z normalization for each patient separately. Then the mean of each 4i readout for each drug treatment was calculated for each patient separately. The resulting vector of drug treatments * 4i readouts corresponds to a patient drug response profile. Drug-feature names were generated by importing the relevant list of drugs. Drug-features associated with the drug treatments “Control” and “Staurosporine” were excluded from further analysis.

##### Whole-Genome Amplification Comparison

Using the provided Whole-Genome Amplification (WGA) index index the patient profiles were either assigned to WGA groups *yes* or *no*. Next, it was determined by the Kruskal-Wallis test on each 4i feature whether the two WGA groups originated from the same probability distribution. Multiple testing was implemented using the Tukey-Kramer method.

### Survival analysis

Relapse-free survival was defined as the time from initial diagnosis to documented disease recurrence or death, whichever occurred first. Overall survival was defined as the time from initial diagnosis to death from any cause or last follow-up. All survival analyses were performed using the Cox proportional hazards model as implemented in the coxph function in the survival R package (version 3.8-3). Results were visualized using survminer (version 0.5.0).

### Analysis of heterogeneity associations

#### Selection of variables and patient stratification

Based on analysis performed by individual nodes using different technologies, derived variables were selected for integrative analysis (Supplementary Data 15). Features contained clinico-pathological characteristics and sample groupings derived from all technologies described in the results section. Pathway scores were calculated based on bulkified gene expression in HGSOC cells from scRNA-seq. Pathways were selected based on their association with intra- and inter- tumor heterogeneity in both bulk- and single-cell RNAseq. Specifically, we took the mean of *t*-statistic from scRNA-seq and bkRNA-seq for the comparisons CyTOF-derived cancer cell heterogeneity_group_intra and heterogeneity_group_inter, and took the 15 largest and smallest values for both comparisons. The pathway selection corresponds to the union of those two sets. All continuous variables were binarized or discretized, thereby stratifying patients into groups for each variable. For the selected pathways, patients were classified into ‘low’ or ‘high’ groups using the median as a cutoff.

#### Overlap analysis

We investigated the overlap between generated patient groups across variables by calculating Jaccard indices and overlap coefficients for patient group pairs as defined below.

For patient groups A and B: Jaccard index J(A, B) = | A ∩ B | / | A∪B| Overlap coefficient O(A,B) = | A ∩ B | / min(|A | , | B | ).

Patient groups with fewer than 4 patients were excluded from this analysis. Further, to calculate the significance of these overlaps between patient groups, we performed permutation testing as follows: for each pair of patient groups generated by two variables, we randomly sampled the same number of patients characterized by each variable as the set size of the corresponding patient group, and calculated the overlap between these randomly sampled groups. For each patient group pair, this was repeated 10^4 times, and the proportion of overlap metrics greater than or equal to the experimentally observed value was reported as the *p*-value. This permutation testing was followed by multiple testing corrections using the Benjamini-Hochberg method. False discovery rates (FDR) obtained for Jaccard indices and overlap coefficients were combined using Fisher’s method to identify significant overlaps (combined FDR < 0.05).

#### Patient grouping similarity analysis

In addition to the overlap analysis, we calculated global similarities for patient groupings generated using our selected variables with the adjusted Rand Index, which measures the similarity of two groupings while correcting for expected agreement by chance^133^. We performed permutation testing for identifying variable pairs with significantly similar groupings (FDR < 0.05) using the same procedure as described above.

### Inclusion & ethics statement

Each collaborator in this study has satisfied the authorship criteria set by Nature Portfolio journals, earning inclusion as an author. Their contributions to planning and performing the study were appropriately acknowledged. Collaborators established roles and responsibilities prior to initiating the research.

### Reporting summary

Further information on research design is available in the Nature Portfolio Reporting Summary linked to this article.

## Supplementary information

Supplementary information

Description of Additional Supplementary Files

Supplementary Data 1

Supplementary Data 2

Supplementary Data 3

Supplementary Data 4

Supplementary Data 5

Supplementary Data 6

Supplementary Data 7

Supplementary Data 8

Supplementary Data 9

Supplementary Data 10

Supplementary Data 11

Supplementary Data 12

Supplementary Data 13

Supplementary Data 14

Supplementary Data 15

Reporting Summary

Transparent Peer Review file

## Source data

Source Data

## Data Availability

The publicly available data used in this study are available from TCGA under https://www.cbioportal.org/study/summary?id=ov_tcga_pan_can_atlas_2018^55^. The raw sequencing data (bulk- and single-cell RNA-seq, single-cell DNA-seq, ctDNA) generated in this study are available from the European Genome-Phenome Archive (EGA) under the following accession codes: EGAD50000001413 (bkRNA-seq), EGAD50000001290 (scRNA-seq), EGAD50000001291 (scDNA), and EGAD50000001410 (ctDNA). The data is available under restricted access due to patient privacy concerns. Access can be requested by contacting the Tumor Profiler Center (TPC) leadership (https://tumorprofilercenter.ch/contacts). Data access will be granted to registered users listed on the with the TPC within four weeks of receipt of the Data Access Agreement, provided that the applicant provides all necessary ethics committee approval and supporting documents needed to meet the requirements of the agreement. The user institution agrees to destroy or discard the data once it is no longer used for the project, and in cases where data must be archived, it must be deleted within 10 years of the project’s completion. If data has not been archived, it must be deleted no later than 2 years following the completion of the project. An extension to this period can be provided upon request to the TPC leadership. The raw proteomics data generated in this study have been deposited in MassIVE under the accession MSV000097828, with the corresponding download link ftp://massive-ftp.ucsd.edu/v09/MSV000097828/. The processed, single-cell resolved data for scRNA-seq, IMC and CyTOF are publicly available on Zenodo: https://zenodo.org/records/19113819^134^. The remaining data are available within the Article, Supplementary Information or Source Data files. In addition, all publicly accessible data is available through the Tumor Profiler data page on https://www.tumorprofiler.com/resources/ovarian. Source data are provided in this paper.

## Code availability

No new algorithms were developed for this manuscript. All analyses were performed using existing tools in R, Python or MATLAB. The code used to reproduce the analysis and figures in this paper is available on Zenodo: https://zenodo.org/records/19116006.
