# Supplementary material for: A tumor profiling resource for ovarian cancer: insights into chemotherapy-driven heterogeneity and personalized treatment strategy
Source: Nat Commun. 2026 Jul 13;17:8624. doi: 10.1038/s41467-026-74585-w (PMC13486661; doi:10.1038/s41467-026-74585-w)
Supplement: Supplementary file 1 [file 41467_2026_74585_MOESM18_ESM.pdf]

nature portfolio

Corresponding author(s): Francis Jacob and Viola Heinzelmann-Schwarz

Last updated by author(s): Nov 28, 2025

Reporting Summary

Nature Portfolio wishes to improve the reproducibility of the work that we publish. This form provides structure for consistency and transparency in reporting. For further information on Nature Portfolio policies, see our Editorial Policies and the Editorial Policy Checklist.

Statistics

For all statistical analyses, confirm that the following items are present in the figure legend, table legend, main text, or Methods section.

n/a	Confirmed	
☐	☒	The exact sample size (*n*) for each experimental group/condition, given as a discrete number and unit of measurement
☐	☒	A statement on whether measurements were taken from distinct samples or whether the same sample was measured repeatedly
☐	☒	The statistical test(s) used AND whether they are one- or two-sided<br>*Only common tests should be described solely by name; describe more complex techniques in the Methods section.*
☐	☒	A description of all covariates tested
☐	☒	A description of any assumptions or corrections, such as tests of normality and adjustment for multiple comparisons
☐	☒	A full description of the statistical parameters including central tendency (e.g. means) or other basic estimates (e.g. regression coefficient) AND variation (e.g. standard deviation) or associated estimates of uncertainty (e.g. confidence intervals)
☐	☒	For null hypothesis testing, the test statistic (e.g. *F*, *t*, *r*) with confidence intervals, effect sizes, degrees of freedom and *P* value noted<br>*Give P values as exact values whenever suitable.*
☒	☐	For Bayesian analysis, information on the choice of priors and Markov chain Monte Carlo settings
☒	☐	For hierarchical and complex designs, identification of the appropriate level for tests and full reporting of outcomes
☐	☒	Estimates of effect sizes (e.g. Cohen's *d*, Pearson's *r*), indicating how they were calculated

Our web collection on statistics for biologists contains articles on many of the points above.

Software and code

Policy information about availability of computer code

Data collection	All data were collected using commercially available kits or previously published methods. Description of each method and references are provided in the Methods section. Use of publicly available data is described in the Methods section.
Data analysis	Computational analysis was performed using standard tools implemented in Matlab, Python, and R with Bioconductor. Details of packages applied is provided in the Methods section.

For manuscripts utilizing custom algorithms or software that are central to the research but not yet described in published literature, software must be made available to editors and reviewers. We strongly encourage code deposition in a community repository (e.g. GitHub). See the Nature Portfolio guidelines for submitting code & software for further information.

Data

Policy information about availability of data

All manuscripts must include a data availability statement. This statement should provide the following information, where applicable:

- Accession codes, unique identifiers, or web links for publicly available datasets
- A description of any restrictions on data availability
- For clinical datasets or third party data, please ensure that the statement adheres to our policy

Publicly available data used in this study are available from TCGA via cBioPortal (https://www.cbioportal.org/study/summary?id=ov_tcga_pan_can_atlas_2018). The raw sequencing data (bulk RNA-seq, single-cell RNA-seq, single-cell DNA-seq, and ctDNA) generated in this study have been deposited in the European

Genome-Phenome Archive (EGA) under accession codes EGAD50000001413, EGAD50000001290, EGAD50000001291, and EGAD50000001410. These data are available under controlled access due to patient privacy restrictions; access can be requested through the Tumor Profiler Center (TPC) (https://tumorprofilercenter.ch/contacts). Raw proteomics data have been deposited in MassIVE under accession MSV000097828.
Processed single-cell data (scRNA-seq, scDNA-seq, IMC, and CyTOF) are available on Zenodo (DOI 10.5281/zenodo.19116006).
Additional data supporting the findings of this study are available within the Article, Supplementary Information, or Source Data files.

Research involving human participants, their data, or biological material

Policy information about studies with human participants or human data. See also policy information about sex, gender (identity/presentation), and sexual orientation and race, ethnicity and racism.

Reporting on sex and gender	All participants in this study were biologically female, as ovarian cancer occurs almost exclusively in individuals with female reproductive organs. Accordingly, sex-based analyses were not applicable. Gender identity was not recorded, as it did not influence study design, clinical decision-making, or molecular analyses.
Reporting on race, ethnicity, or other socially relevant groupings	Information on race, ethnicity or other socio-economical parameters were not collected for this cohort.
Population characteristics	All clinicopathological data are summarized in Supplementary table S1 and clinical parameters per sample are provided in Supplementary Data 1.
Recruitment	Inclusion criteria encompassed patients aged 18 years or older with an ECOG performance status score of 2 or lower, and a primary or recurrent high-grade serous adenocarcinoma of ovarian, tubal, or peritoneal origin. Exclusion criteria involved serious medical, psychiatric, psychological, familial, or geographical conditions that might interfere with the project, as determined by the project leader, and legal incompetence. Additional information are provided on clinicaltrials.gov: Establishment and Standardization of a Platform for In-depth Tumour Profiling (TUPRO) in Patients With Advanced and Metastatic High-Grade Adenocarcinoma of Ovarian, Tubal or Peritoneal Origin (TUPRO-Gyn); ClinicalTrials.gov ID NCT06599749.
Ethics oversight	Written informed consent was granted by all patients and ethical allowance granted by the Ethics Committee Northwest and Central Switzerland (EKNZ, TuPro; BASEC: 2018-02052).

Note that full information on the approval of the study protocol must also be provided in the manuscript.

Field-specific reporting

Please select the one below that is the best fit for your research. If you are not sure, read the appropriate sections before making your selection.

☒ Life sciences ☐ Behavioural & social sciences ☐ Ecological, evolutionary & environmental sciences

For a reference copy of the document with all sections, see nature.com/documents/nr-reporting-summary-flat.pdf

Life sciences study design

All studies must disclose on these points even when the disclosure is negative.

Sample size	For TuPro in ovarian cancer, a total of 62 patients were prospectively enrolled between December 2018 and March 2021 across three Swiss hospitals. Due to the exploratory and observational nature of this study, no formal sample size calculation was performed. Instead, sample size was determined by patient availability within the study period and predefined inclusion criteria. This cohort size was considered sufficient to enable feasibility assessment and to capture inter-patient heterogeneity across the multimodal analyses.
Data exclusions	Due to limited availability of samples and resources, not all samples were measured by all technologies. Information are summatized in Extended Data Fig. 2.
Replication	Key findings in this study were supported by orthogonal approaches across multiple technologies and samples, and were consistent with previously published data and reanalysis of independent datasets. As this study is based on prospectively collected patient samples, experimental replication in the traditional sense was not applicable; however, results were reproducible across independent patient samples and modalities. No findings were identified that could not be reproduced.
Randomization	This is an observational study and focuses on 1) feasibility and 2) exploratory analysis, therefore there was no need for randomization.
Blinding	This is an observational study and focuses on 1) feasibility and 2) exploratory analysis, therefore there was no need for blinding.

Reporting for specific materials, systems and methods

We require information from authors about some types of materials, experimental systems and methods used in many studies. Here, indicate whether each material, system or method listed is relevant to your study. If you are not sure if a list item applies to your research, read the appropriate section before selecting a response.

Materials & experimental systems

n/a	Involved in the study
☐	☒ Antibodies
☒	☐ Eukaryotic cell lines
☒	☐ Palaeontology and archaeology
☒	☐ Animals and other organisms
☐	☒ Clinical data
☒	☐ Dual use research of concern
☒	☐ Plants

Methods

n/a	Involved in the study
☒	☐ ChIP-seq
☒	☐ Flow cytometry
☒	☐ MRI-based neuroimaging

Antibodies

Antibodies used: All necessary details are provided in Supplementary Data 14 "Antibody panels".

Validation: All used antibodies are quality control tested by manufacturer using immunofluorescent staining with flow cytometric analysis.

Clinical data

Policy information about clinical studies

All manuscripts should comply with the ICMJE guidelines for publication of clinical research and a completed CONSORT checklist must be included with all submissions.

Clinical trial registration: BASEC: 2018-02052 and NCT06599749

Study protocol: The study protocol is not publicly available, but was made available to all patients prior to study enrollment.

Data collection: All patients in this study were enrolled between December 2018 and March 2021 at three Swiss hospitals: University Hospital Basel, Cantonal Hospital Liestal, and University Hospital Zurich.

Outcomes: The TuPro study is a prospective observational clinical study. The primary objective was to evaluate the feasibility of multi-modal data acquisition within a clinically relevant timeframe and its utility for hypothetical treatment recommendations. Only maintenance therapy recommendations were adapted based on molecular guidance where ethically permissible. Recurrence-free survival and overall survival were assessed as clinical outcome measures.

Plants

Seed stocks: *Report on the source of all seed stocks or other plant material used. If applicable, state the seed stock centre and catalogue number. If plant specimens were collected from the field, describe the collection location, date and sampling procedures.*

Novel plant genotypes: *Describe the methods by which all novel plant genotypes were produced. This includes those generated by transgenic approaches, gene editing, chemical/radiation-based mutagenesis and hybridization. For transgenic lines, describe the transformation method, the number of independent lines analyzed and the generation upon which experiments were performed. For gene-edited lines, describe the editor used, the endogenous sequence targeted for editing, the targeting guide RNA sequence (if applicable) and how the editor was applied.*

Authentication: *Describe any authentication procedures for each seed stock used or novel genotype generated. Describe any experiments used to assess the effect of a mutation and, where applicable, how potential secondary effects (e.g. second site T-DNA insertions, mosiacism, off-target gene editing) were examined.*
