# Supplementary material for: A tumor profiling resource for ovarian cancer: insights into chemotherapy-driven heterogeneity and personalized treatment strategy
Source: Nat Commun. 2026 Jul 13;17:8624. doi: 10.1038/s41467-026-74585-w (PMC13486661; doi:10.1038/s41467-026-74585-w)
Supplement: Supplementary file 2 [file 41467_2026_74585_MOESM19_ESM.pdf]

nature portfolio

Peer Review File

A tumor profiling resource for ovarian cancer: insights into chemotherapy-driven heterogeneity and personalized treatment strategy

Corresponding Author: Dr Francis Jacob

This file contains all reviewer reports in order by version, followed by all author rebuttals in order by version.

Version 0:

Reviewer comments:

Reviewer #1

(Remarks to the Author)
The manuscript investigates the high heterogeneity of high-grade serous ovarian cancer (HGSOC) and highlights the potential of combining genetic landscapes with tumor microenvironment (TME) interactions to enhance precision oncology and improve patient outcomes. The researchers developed a comprehensive workflow in the TuPro trial to detect multiple molecular signatures and provide guidance for clinical treatments. This workflow allows the measurement of various biomarkers influencing treatment decisions and includes multi-omics data from clinical samples at multiple time points, including solid tumors, ascites, and blood. The authors aim to integrate these multi-omics data to characterize TME composition, assess chemotherapy-induced tumor changes, and evaluate genomic signatures.

The workflow's ability to perform comprehensive analyses within four weeks and its applicability in clinical settings are remarkable and represent a unique strength of the study. However, the effectiveness of the updated therapy could be better demonstrated, as the decision-making process of the Molecular Tumor Board (MTB) and how TuPro influences treatment remains unclear.
The integration of multi-omics data is another notable strength of this study. The breadth of data across patients and clinical time points presents a valuable resource for the field. However, while modality-specific analyses are well presented, the deeper integration of these technologies remains lacking. In addition, as the title of the manuscript is a resource - the data should be made publicly available to increase the impact of the work as a resource. This can be done in many ways that are compatible with patient privacy - including web portals for visualization etc.

Major Comments:
The manuscript claims that TuPro offers utility beyond FoundationOne (FMI) genomic testing, with the clinical team evaluating its usefulness through categorized assessments. However, this is primarily a descriptive analysis, not a true comparative analysis. To strengthen this claim, we recommend:

(1) Performing a benchmarking of clinical outcomes (e.g., relapse-free survival) between patients who received altered vs unaltered recommendations.

(2) If clinical outcome comparisons are not feasible, consider including case-level narratives to demonstrate the differential impact of FMI vs TuPro-guided treatment suggestions. Could the authors provide 1-2 cases where multi-modal data concretely changed treatment direction?

While the manuscript emphasizes the power of multi-omics integration, the analyses remain largely modality-specific. Although the study successfully combines bulk RNA-seq and scRNA-seq to characterize molecular subtypes of HGSC, there is a missed opportunity to present a multi-modal integration framework (e.g., consensus clustering, patient-level decision trees, or graph-based integration of drug response and omics features). This would provide a more integrated, comprehensive view of the data.

Minor Comments:
In the results, the authors mention "A total of 87 samples were obtained from 62 patients, resulting in 66 samples from pre-determined 'priority sites,'" but only n=39 samples from priority sites are shown in Figure X (labeled with *). Please clarify this discrepancy.

Regarding the statement, “This evaluation revealed that the majority of priority samples arose from patients carrying an immune-excluded or -inflamed TME, with a trend towards low PD-1 and PD-L1 expression,” please comment on whether these features are representative of the broader HGSC population. Does this potential bias limit the generalizability of the findings?

What criteria did the Molecular Tumor Board (MTB) use to categorize treatment changes as “Very,” “Largely,” “Partially,” or “Somewhat”? Was this assessment based on a structured questionnaire, Likert scale, or informal consensus? Please clarify the methodology used for these evaluations.

The authors note discrepancies between dissociation-based and tissue-based cell compositions, yet some conclusions (e.g., immune exclusion status, EMT markers) still rely on dissociated data. Will this difference introduce bias into the conclusions?

The manuscript would benefit from a dedicated limitations section, addressing the observational nature of the study, potential batch effects between platforms, and technology-specific dropout or sample failures.

While datasets (except for public datasets) are not publicly available due to patient privacy, the analysis scripts are also not accessible. To improve reproducibility, please consider providing the analysis code or making it available upon request.

Reviewer #2

(Remarks to the Author)
I co-reviewed this manuscript with one of the reviewers who provided the listed reports. This is part of the Nature Communications initiative to facilitate training in peer review and to provide appropriate recognition for Early Career Researchers who co-review manuscripts.

Reviewer #3

(Remarks to the Author)
This manuscript reports the findings of the Tumor Profiler observational trial, in which samples from 62 HGSOC patients were extensively profiled using multiple methodologies, with almost 75% of the priority samples undergoing analysis by at least 9 of 11 technologies. The authors have done an excellent job in placing their findings in the context of the current HGSOC profiling literature and adding interesting new data particularly on whole genome amplification and tumor microenvironment changes. Overall, the manuscript is well-written.

The manuscript would be strengthened by addressing a number of specific additional areas as follows.
Major:
1. It is unclear how the initial patient cohort was diagnosed with HGSOC. By conventional H+E pathology? Was there central review and/or re-review prior to analysis? The data shown in Figure 1B suggest a relatively large subset of cases for which none of PAX8, WT1, and p53 data are available. How was the diagnosis made in these cases, given these are frequent IHC methods for diagnosis of HGSOC?
2. The authors highlight that one of the major areas of impact of their work is the potential change in clinical management from multi-omics findings that extend beyond just genomic assessments. Please provide the actual changes in treatment recommendation provided by TuPro, as this information is necessary to evaluate the true clinical impact as suggested by the authors.
3. Figure 2. This is a mix of both biomarkers and drug response assays (drug response profiling and pharmacoscopy). The relative contribution of these different sets of data to treatment decisions is hard to tease out as shown, and would be clearer if the molecular and drug screening assays were separated out. Of the two categories, which is more commonly driving treatment changes?

Minor:
1. A number of box-and-whisker plots lack y-axis labels, e.g., Figure 7A right panel, Figure 6H top panel. Please check throughout.
2. Please clarify how the pH2AX biomarker influenced treatment decisions.

Reviewer #4

(Remarks to the Author)
This paper presents findings from the Tumor Profiler (TuPro) observational study, a comprehensive multi-omic profiling initiative for patients with high-grade serous ovarian cancer (HGSOC). The authors applied up to 11 high-resolution DNA, RNA, protein, and functional platforms on samples from 62 patients, integrating single-cell, spatial, and ex vivo drug-response data. A key innovation is the delivery of clinically actionable data within a 4-week turnaround, used to guide hypothetical treatment decisions via molecular tumor boards (MTBs). The study highlights how chemotherapy alters cancer cell heterogeneity, and identifies whole-genome amplification (WGA) as a poor prognostic factor.

Detailed comments:

1. The authors state that “In 21 out of 39 cases (54%) where TuPro altered the treatment decision, this change was also reflected in the physician’s final treatment choice.” Would it be possible to compare clinical outcomes between patients whose treatments aligned with the TuPro recommendations and those whose did not? Even an exploratory retrospective analysis could provide valuable insight into the clinical impact of implementing TuPro-guided decisions and would help strengthen the study’s claims regarding clinical relevance. Also, it would be interesting to see whether the effect of TuPro-informed treatment decisions on clinical outcomes differs by molecular subgroup (e.g., WGA vs. non-WGA, HRD vs. HRP).

2. The paper notes that while TuPro recommendations influenced treatment decisions in 76% of cases, most changes were related to maintenance therapy (e.g., addition of letrozole, bevacizumab, or PARPi), and not to first-line (neo)adjuvant chemotherapy, which was generally administered per standard of care. This distinction is important because it raises the question of how early in the treatment course TuPro data can have actionable value. Can the authors clarify:
• What proportion of patients received a TuPro-guided change to frontline therapy versus maintenance therapy?
• Whether the timing of TuPro recommendations (e.g., pre- vs. post-surgery) allowed meaningful integration into clinical decision-making?
• If there were any measurable short-term clinical effects (e.g., delayed progression, biomarker responses, fewer adverse events) in patients receiving TuPro-guided maintenance therapy?

3. The paper presents several notable associations, such as the link between whole genome amplification (WGA) and earlier disease recurrence, and suggests that TuPro profiling offers added value beyond standard genomic tests like FoundationOne CDx. However, the paper does not present formal statistical models to evaluate how the integration of TuPro data improves prognostic or predictive accuracy. Have the authors considered multivariate survival models (e.g., Cox regression) that include clinical variables, standard genomic, and TuPro-derived new variables to quantify the incremental prognostic value of TuPro? Reporting model performance metrics such as the concordance index (C-index), Akaike Information Criterion (AIC), or likelihood ratio tests would strengthen this claim.

4. The authors report that CN signatures 4 and 7 are enriched in samples with whole genome amplification (WGA), and that these signatures are associated with focal amplifications and genome doubling, respectively. However, the clinical implications of these findings remain unclear. Can the authors elaborate on whether these CN signatures provide predictive value for treatment response, beyond their descriptive association with WGA and genomic instability?

5. While the study shows modest differences between solid tumor and ascites samples, only a limited number of matched samples were profiled across all modalities. Can the authors specify the n for paired comparisons per platform? Similarly, for patients with longitudinal samples, can the authors provide more detail (e.g., a table) describing temporal changes in molecular features or treatment outcomes?

6. The paper suggests that TuPro data could guide frontline treatment de-escalation or substitution, yet the current cohort is largely retrospective and observational. Are there plans for a prospective interventional trial specifically testing TuPro-guided first-line therapy?

Version 1:

Reviewer comments:

Reviewer #1

(Remarks to the Author)
The authors hase mostly answered my comments. The only comment remaining is that the word integration is used interchangeably to incorporation and correlation. In their rebuttal letter they refer to integration, but finally the MOFA analysis did not reveal anything and was not added to the manuscript, and our comment on integration did not result in any change in the manuscript. As real integration of Multi-omics data is not presented in the manuscript, this should also be added to limitations in the discussion.

The word integration should be checked across the manuscript and changed. E.g.

Row 153 - Integration of a clinically implemented platform - do you mean application or usage? Discussion, first chapter, and

Line 470 Integration of multi-omics data - It is actually incorporation.

In Fig legend 6 F - Integration is more like a correlation? (shown in G).

Line 436 - inter-sample integration - what do you mean by this integration - likely not the right word for this. Intermixing? Please clarify.

Reviewer #2

(Remarks to the Author)
I co-reviewed this manuscript with one of the reviewers who provided the listed reports. This is part of the Nature Communications initiative to facilitate training in peer review and to provide appropriate recognition for Early Career Researchers who co-review manuscripts.

Reviewer #3

(Remarks to the Author)
Thank you for thoroughly addressing the prior feedback, which has strengthened the manuscript.

Reviewer #4

(Remarks to the Author)
The authors have provided thoughtful responses, and the addition of survival analyses together with clarification regarding the timing of TuPro-guided decisions meaningfully strengthens the manuscript. However, several issues related to the interpretation of clinical benefit and the incremental value of TuPro remain insufficiently addressed. My follow-up comments are below.

1. The newly added Kaplan–Meier analysis suggests improved overall survival among newly diagnosed patients whose treatment was influenced by TuPro recommendations (log-rank $p = 0.026$; HR = 0.36 [0.14–0.91]). While encouraging, additional clarification is needed to assess robustness.

Please clarify the analytic population and time origin for the OS analysis in Fig. 2E. Was OS measured from diagnosis, treatment initiation, or the start of maintenance? Because TuPro recommendations primarily affected maintenance therapy, defining time-zero earlier than maintenance may introduce immortal-time bias. If OS was anchored earlier, a landmark or time-dependent Cox analysis would help mitigate this concern.

The manuscript presents an adjusted Cox model for recurrence-free survival, but the OS analysis in Fig. 2E appears unadjusted. If feasible, please report an adjusted OS model including key prognostic covariates (e.g., stage, residual disease, BRCA/HRD status, age, ECOG). Even exploratory estimates would help determine whether the association persists after accounting for baseline differences.

2. In the rebuttal, the authors note that likelihood-ratio statistics were incorporated for outcome analyses related to treatment changes to evaluate the incremental prognostic value of TuPro. Could you clarify where these LR statistics are presented in the manuscript? From the text and figures, the only reported outcome comparisons appear to be log-rank tests (Fig. 2E), and I did not identify likelihood-ratio tests comparing nested Cox models. If LR analyses were performed, please specify where they are reported and describe the models being compared.

Version 2:

Reviewer comments:

Reviewer #1

(Remarks to the Author)
The authors have now addressed my final comment.

Reviewer #2

(Remarks to the Author)
I co-reviewed this manuscript with one of the reviewers who provided the listed reports. This is part of the Nature Communications initiative to facilitate training in peer review and to provide appropriate recognition for Early Career Researchers who co-review manuscripts.

Reviewer #4

(Remarks to the Author)
The additional adjusted analysis and clarification of methods meaningfully strengthen the manuscript. I appreciate the

inclusion of the multivariable OS model and transparent reporting of effect sizes and confidence intervals. The interpretation as exploratory and hypothesis-generating is appropriate.

Open Access This Peer Review File is licensed under a Creative Commons Attribution 4.0 International License, which permits use, sharing, adaptation, distribution and reproduction in any medium or format, as long as you give appropriate credit to the original author(s) and the source, provide a link to the Creative Commons license, and indicate if changes were made.
In cases where reviewers are anonymous, credit should be given to 'Anonymous Referee' and the source.
The images or other third party material in this Peer Review File are included in the article's Creative Commons license, unless indicated otherwise in a credit line to the material. If material is not included in the article's Creative Commons license and your intended use is not permitted by statutory regulation or exceeds the permitted use, you will need to obtain permission directly from the copyright holder.
To view a copy of this license, visit https://creativecommons.org/licenses/by/4.0/

Reviewer feedback:

Reviewer #1 (Remarks to the Author)

1. The manuscript investigates the high heterogeneity of high-grade serous ovarian cancer (HGSOC) and highlights the potential of combining genetic landscapes with tumor microenvironment (TME) interactions to enhance precision oncology and improve patient outcomes. The researchers developed a comprehensive workflow in the TuPro trial to detect multiple molecular signatures and provide guidance for clinical treatments. This workflow allows the measurement of various biomarkers influencing treatment decisions and includes multi-omics data from clinical samples at multiple time points, including solid tumors, ascites, and blood. The authors aim to integrate these multi-omics data to characterize TME composition, assess chemotherapy-induced tumor changes, and evaluate genomic signatures.The workflow's ability to perform comprehensive analyses within four weeks and its applicability in clinical settings are remarkable and represent a unique strength of the study. However, the effectiveness of the updated therapy could be better demonstrated, as the decision-making process of the Molecular Tumor Board (MTB) and how TuPro influences treatment remains unclear. The integration of multi-omics data is another notable strength of this study. The breadth of data across patients and clinical time points presents a valuable resource for the field. However, while modality-specific analyses are well presented, the deeper integration of these technologies remains lacking. In addition, as the title of the manuscript is a resource - the data should be made publicly available to increase the impact of the work as a resource. This can be done in many ways that are compatible with patient privacy - including web portals for visualization etc.

Response: We thank the reviewer for recognizing the strength of our multi-omics data and for highlighting the importance of accessibility. In line with this comment, we have now included a statement in the Data Availability section specifying that all publicly accessible Tumor Profiler datasets, including those from the ovarian cancer cohort, are available via the Tumor Profiler web portal (https://www.tumorprofiler.com).

Action: We have updated the data availability section. In addition, all publicly accessible data is available through the Tumor Profiler data page at https://www.tumorprofiler.com/resources/ovarian. Access is currently restricted to reviewers with username **reviewer** and password **_tupro_ovca_239**, but will be released upon publication of this manuscript."

We have also revised the section describing the setup, decision-making process of the molecular tumor board (MTB) and the following clinical implications. The corresponding text now reads:

Multi-omics tumor profiling alters treatment recommendations

"The TuPro data were aggregated and summarized within a four-week turnaround time and subsequently presented to a dedicated molecular tumor board (MTB). Each MTB involved a multidisciplinary team of approximately 16 participants, including gynecologic and medical oncologists, molecular pathologists, and scientists representing each TuPro technology platform. To ensure clinical relevance and transparency, every MTB followed a standardized three-step workflow.

First, the treating physicians presented the clinical case, including disease history and, if applicable, prior therapies, and outlined a treatment plan based on current clinical guidelines (e.g., ESGO/ESMO consensus recommendations). Second, targeted genomic profiling data generated by FMI were reviewed, and the MTB assessed whether these results would support, refine, or change the guideline-based treatment strategy—classified as major, minor, confirmatory, or no evidence for treatment modification. This step reflected the integration of a clinically implemented genomic platform used in some clinical centers, allowing assessment of how useful additional genomic information was in refining standard-of-care treatment decisions. Third, the integrated TuPro multi-omics and functional data were discussed, which represents the new and unique opportunity of the TuPro study. The MTB then re-evaluated the case and determined whether the TuPro dataset provided supporting or conflicting evidence that might alter the therapeutic recommendation made in the previous two steps. Across all cases, analysis of the decision reports revealed that each TuPro technology contributed to at least one MTB by providing supportive biomarkers or functional validation from *ex vivo* assays. Several biomarkers were recurrently considered in treatment discussions, with *BRCA1/2* status, phosphorylated mTOR (pmTOR), and γH2AX among the most influential (**Fig. 2A**). Overall, the inclusion of TuPro data significantly increased the proportion of patients with a minor treatment change (drug change within the same drug class) from 4% to 37% (Pearson's Chi-squared test: χ^2 = 15.35, df = 1, p = 8.9 × 10^{-5}), while reducing cases with no evidence for a change from 51% to 8% (χ^2 = 20.83, df = 1, p = 5.0 × 10^{-6}) (**Fig. 2B** and **Extended Data Fig. 4**). When considering changes of a drug class by either replacing, removing, or adding a new treatment ('major treatment change'), we observed a moderate increase in the likelihood of a treatment change from 24% using FMI only (p = 0.14) to 39% using TuPro technologies (**Fig. 2B**). Importantly, these recommendations were independent of clinical treatment setting (chemo-naive, post-NACT, or recurrent disease) (Pearson's Chi-squared test: $\chi2$=2.51, df=4, p=0.64; $\chi2$=5.60, df=4, p=0.23, respectively) (**Fig. 2C**). The molecular summary report provided at each MTB was considered clinically relevant for the majority of patients ('very' and 'largely useful, 30/51, 60.1%, and **Fig. 2D**, see Methods for details). In 21 out of 39 cases (54%) where TuPro altered the treatment decision, this change was also reflected in the physician's final treatment choice. As this trial was an exploratory setup, from an ethical point of view we could not change acute therapies in the frontline or second-line setting. In addition, the study cohort lacked a significant number of patients beyond standard-of-care treatment, where such modifications could have been ethically permissible. Therefore, the only adaptations made based on the MTB in this trial, were in regards to the maintenance therapy following standard-of-care chemotherapy. These alterations were involving the choice and type of PARPi, or the addition of letrozole, metformin or bevacizumab (**Fig. 2E** and **2F**, see **Supplementary Data 12** for details). In the group of newly diagnosed patients (chemo-naïve or post-NACT) this maintenance alteration led to a significant overall survival benefit in patients compared to patients where TuPro did not alter the treatment (log-rank p = 0.026; HR = 0.36 (0.14-0.91)); **Fig. 2E**). While this represents a first indication that should be interpreted with caution given the lack of randomization, these findings suggest a potential clinical impact of TuPro-guided recommendations.

In summary, our proposed framework for evaluating the clinical usefulness of routine and emerging diagnostics in combination with experimental multi-modal tumor profiling in a representative HGSOC cohort (**Extended Data Fig. 4**) enabled personalized drug recommendations independently of treatment status.

This approach demonstrates a feasible precision-oncology workflow that can inform treatment decisions beyond standard-of-care therapy.”

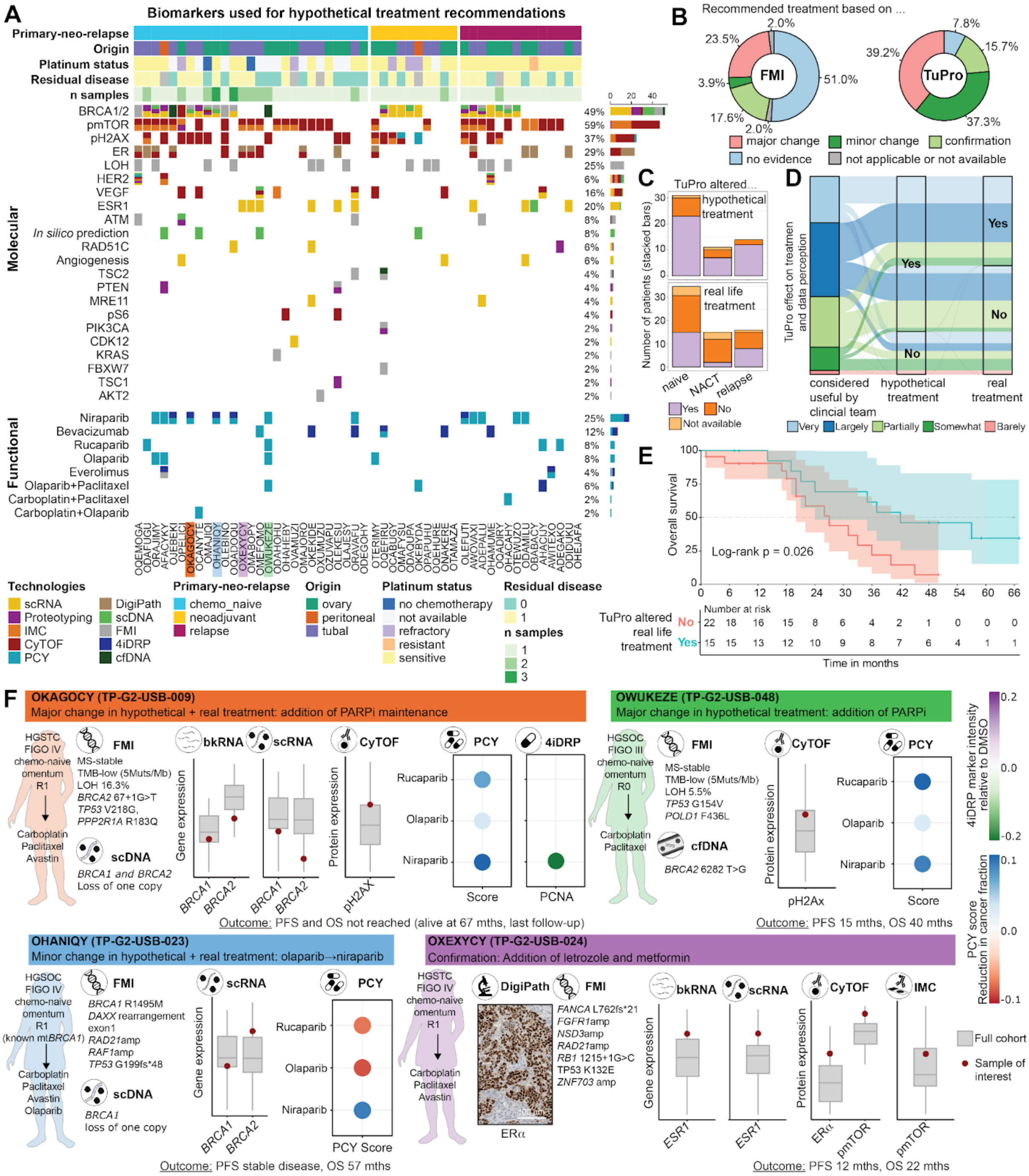

Figure 2. Biomarker-driven treatment recommendations and the impact of TuPro data on hypothetical and real clinical decision-making.

A) Overview of biomarkers and technologies contributing to hypothetical treatment recommendations. Oncoprint summarizes biomarker findings stratified by clinical setting (chemo-naive, neoadjuvant chemotherapy (NACT), and relapse), with contributing technologies color-coded. The proportion of patients for whom each biomarker informed a

treatment recommendation is shown as a bar chart on the right. **B)** Pie charts comparing MTB treatment recommendations based on routine clinical assessment, FMI-derived targeted genomic profiling, and TuPro-integrated multi-omics data. **C)** Stratification of hypothetical (left) and real (right) TuPro-altered treatment recommendations across chemo-naive, neoadjuvant (NACT), and relapse patients, shown as stacked bar charts (total number of time points indicated). **D)** Alluvial plot illustrating the perceived usefulness of each MTB, as rated by participating clinicians, further split into hypothetical and real treatment recommendations. **E)** Kaplan-Meier analysis of overall survival for all newly diagnosed patients (chemo-naive and post-NACT patients). Shaded regions represent 95% confidence intervals; *p*-value from log-rank test. **F)** Selected patient cases illustrating FMI- and TuPro-informed treatment modifications. Representative examples highlight clinical details, molecular findings, and multi-omic evidence (FMI, cfDNA, scDNA, scRNA, CyTOF, PCY, 4iDRP, DigiPath, IMC) supporting hypothetical and real treatment recommendations, including changes in PARP inhibitor maintenance. Box plots show the median (horizontal line), interquartile range (box), and whiskers extending to 1.5x the interquartile range. See also Extended Data Fig. 4, Supplementary Data 12. Source data are provided as a Source Data file.

Major Comments:

2. The manuscript claims that TuPro offers utility beyond FoundationOne (FMI) genomic testing, with the clinical team evaluating its usefulness through categorized assessments. However, this is primarily a descriptive analysis, not a true comparative analysis. To strengthen this claim, we recommend:

Response: We thank the reviewer for this thoughtful comment. We agree that the current analysis is descriptive, as the TuPro ovarian cancer study was designed as a non-interventional feasibility trial rather than a study powered to assess clinical benefit. Its primary objective was to evaluate whether comprehensive multi-omics and functional profiling could be implemented within a clinically meaningful timeframe and provide actionable information beyond clinical routine diagnostics and conventional genomic profiling. Importantly, changes to first-line treatment were neither intended nor permitted within the scope of the trial, as deviations from established ESGO/ESMO clinical guidelines would be unethical—particularly in newly diagnosed patients. Consequently, TuPro was designed to inform, rather than dictate, therapeutic decisions, with all recommendations discussed in the molecular tumor board remaining advisory and non-binding.

We now emphasize this point in the added “Limitations section” and note that the clinical impact of TuPro-guided recommendations is being prospectively assessed in an ongoing follow-up interventional trial. In addition, we have performed a follow-up analysis on the TuPro patients and added new findings to Fig. 2 (see response to comment 1 made by reviewer 1 above)

Action:

We have added limitations of the study to the end of the discussion.

This clinical trial employed multimodal profiling using up to eleven technologies to investigate HGSOC samples collected at different disease time points. Designed as a non-interventional feasibility study, it aimed to evaluate the practicality, turnaround time, and added value of integrating multi-omics and functional profiling into clinical decision-making workflows, rather than to assess direct clinical benefit. While the descriptive nature of the analysis precludes causal inference regarding treatment efficacy, the insights gained

have informed a prospective interventional follow-up trial that will systematically evaluate the clinical impact of TuPro-guided recommendations. The applied technologies—implemented here in unprecedented breadth—characterized tumor cell composition together with genetic, transcriptomic, and proteomic features, as well as functional *ex vivo* drug responses. Although technologically comprehensive, the approach did not fully capture the phosphoproteome, metabolome, or epigenome, molecular layers that could provide additional markers such as pERK (MEK inhibitor response [1,2]) or *BRCA1* promoter methylation (PARPi sensitivity [3]). Variation in resource availability and technology access across TuPro nodes may also have influenced consistency between centers, while a limited number of matched and longitudinal samples—largely due to budget constraints—reduced statistical robustness and temporal resolution. In regard to the cohort, its composition primarily represents advanced-stage, chemo-naïve HGSOC and therefore may not fully capture the immune diversity observed in early-stage or HRD-positive disease. This study also revealed that differences between profiling modalities mainly reflect the intrinsic biology of the sampled compartments rather than methodological artifacts. These findings emphasize the value of integrated multi-modal tumor profiling, as molecular tumor board recommendations are shaped by complementary layers of evidence that cannot be captured by a single technology alone. Future interventional studies will systematically compare immune phenotypes across primary and metastatic sites, with specific emphasis on the anatomical side of sampling. Finally, potential treatment adaptations identified here remain hypothetical, as the technologies employed are not yet CE-certified for clinical use. These constraints define the ethical boundaries of the current study but also underscore the need for further research in personalized oncology to translate biology-driven treatment decisions into certified clinical settings.

---

3. Performing a benchmarking of clinical outcomes (e.g., relapse-free survival) between patients who received altered vs unaltered recommendations.

Response/Action: We thank the reviewer for this valuable suggestion. The TumorProfiler Ovarian Cancer study was designed as a registered observational clinical study (*NCT06599749; "Establishment and Standardization of a Platform for In-depth Tumour Profiling (TUPRO) in Patients With Advanced and Metastatic High-Grade Adenocarcinoma of Ovarian, Tubal or Peritoneal Origin (TUPRO-Gyn)"*). Its primary aim was to evaluate the feasibility and clinical utility of multi-omics tumor profiling in guiding decision-making, and to compare the biological insights gained from these technologies with those obtained from routine clinical diagnostics.

All treating physicians had access to the TuPro data; however, there was no randomization, and final treatment decisions were made independently according to standard clinical guidelines. In patients with newly diagnosed high-grade serous ovarian cancer, these guidelines in 2019-2021 included carboplatin–paclitaxel-based chemotherapy with optional maintenance therapy using bevacizumab (for FIGO stage IV or R1 disease) or olaparib (or patients with a strong family history suggestive of *BRCA*ness or a known germline or somatic *BRCA1/2* mutation). Due to obvious ethical constraints, first-line chemotherapy regimens could not be altered based on TuPro results. Instead, TuPro primarily informed maintenance treatment choices, where physicians could consider molecular and functional findings when selecting PARP inhibitors or

introducing additional agents such as letrozole, metformin, or bevacizumab. To make the exact changes made based on TuPro guidance more clear, we have now added the exact details of how TuPro influenced the treatment decisions to Supplementary Data 12 (column M: comment_TuPro_altered_treatment). Moreover, we have performed a follow-up analysis on the cohort and included data in the manuscript (see response to comment 1 made by reviewer 1 above).

---

4. While the manuscript emphasizes the power of multi-omics integration, the analyses remain largely modality-specific. Although the study successfully combines bulk RNA-seq and scRNA-seq to characterize molecular subtypes of HGSC, there is a missed opportunity to present a multi-modal integration framework (e.g., consensus clustering, patient-level decision trees, or graph-based integration of drug response and omics features). This would provide a more integrated, comprehensive view of the data.

Response: We have initially tried to perform an unsupervised clustering of all patients based on all data modalities using multi-omics factor analysis (MOFA, see **Rebuttal Figure 1** below). However, the results were highly dependent on which features were selected for input to MOFA (e.g. whether we included the top 250 or top 500 most variable genes from bulk or scRNA-seq, whether we used tumor-content corrected values from bulk technologies or not etc.) and we did not obtain a stable patient grouping. Therefore, and to better streamline the manuscript, we decided to instead focus on specific features of ovarian cancer, like for instance whole genome amplification or tumor heterogeneity, and characterize these features using all of the available data modalities. We agree with the reviewer that there would be many additional angles to explore. In this paper, we highlight findings from our own analysis, but also provide all of the underlying data on our website www.tumorprofiler.com (ovarian cancer data access currently restricted to reviewers, with user name *reviewer* and password *_tupro_ovca_239*), providing an extensive resource to the community that can be further explored.

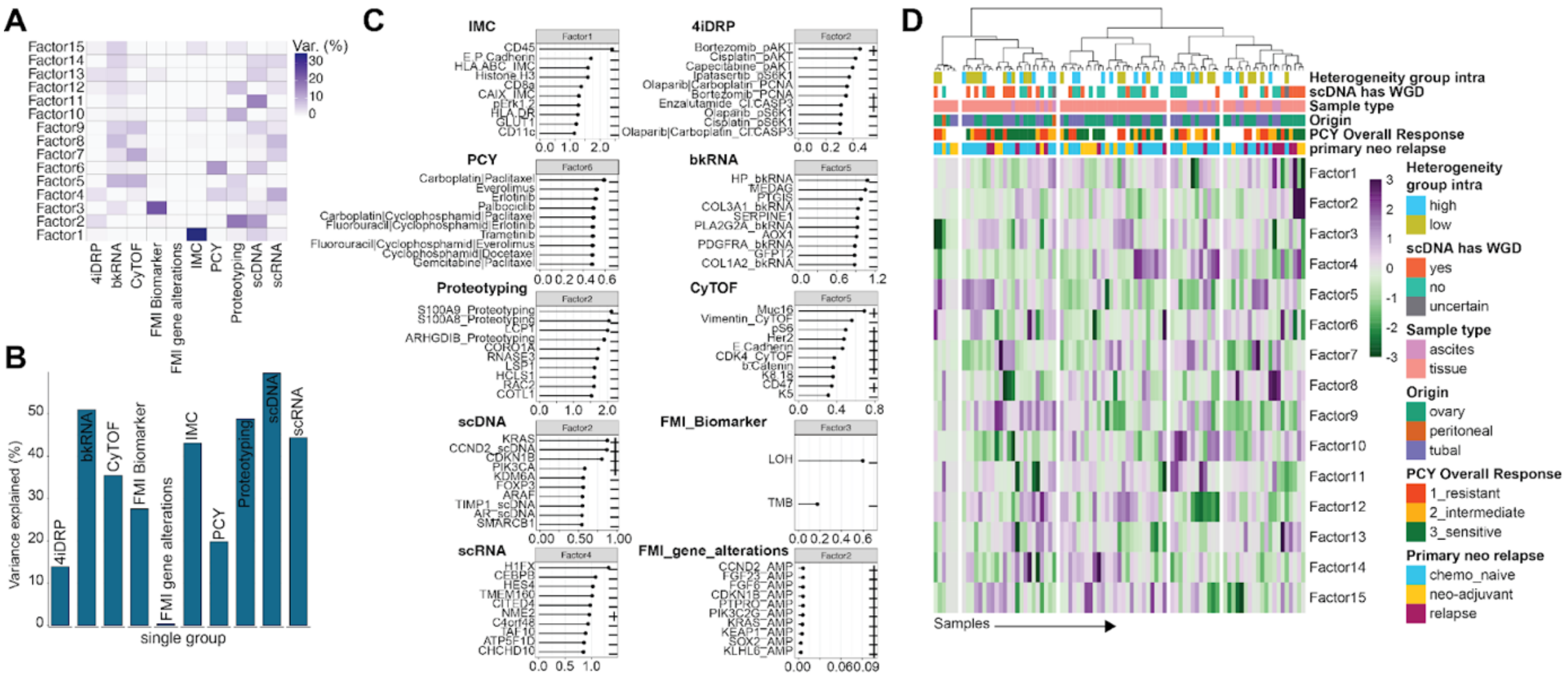

Rebuttal Figure 1. Exploratory Multi-Omics Factor Analysis (MOFA) showing shared and assay-specific sources of variation across the TuPro HGSOC cohort. **A)** Proportion of total variance explained (R^2) by each factor (rows) across the different omics layers (columns) included in the model. Each column represents an assay (4iDRP, bulk RNA-

seq, CyTOF, IMC, proteotyping, scDNA-seq, scRNA-seq, Foundation Medicine gene alterations, and associated biomarkers). **B)** Cumulative variance explained by each single omics group, illustrating the relative contribution of each data modality to the global model. **C)** Exemplary top factors and their strongest molecular feature loadings per TuPro technology. Shown are absolute loading values for the genes, proteins, or markers with the highest weights. Plus (+) or minus (–) signs at the right side of the plot indicate the direction of the loading. **D)** Heatmap visualization of the top 15 factors derived from MOFA, with samples (columns) hierarchically clustered (euclidean distance, complete linkage) based on their factor values (rows). Annotations on top indicate key clinical and molecular features, including intratumoral heterogeneity group, WGD (whole-genome duplication) status from scDNA-seq, sample type, tumor origin, ex vivo drug response (PCY overall response), and clinical treatment setting (chemo-naïve, neoadjuvant, or relapse). Together, the analysis highlights both shared and modality-specific latent factors capturing major axes of molecular and phenotypic heterogeneity in the TuPro HGSOC cohort.

<u>Minor Comments:</u>

5. In the results, the authors mention "A total of 87 samples were obtained from 62 patients, resulting in 66 samples from pre-determined 'priority sites,'" but only n=39 samples from priority sites are shown in Figure X (labeled with *). Please clarify this discrepancy.

Response: We thank the reviewer for noting this point and apologize for the unclear visualization. In several patients, more than one specimen was collected from the same time point; however, not all TuPro technologies had the capacity to process multiple samples per patient. To ensure consistency across analyses, we therefore defined a priority sample for each patient based on the anatomical site of collection, following a pre-specified hierarchy: (1) omental metastasis, (2) other tissue (distant metastasis or primary tumor), and (3) ascites. The “TuPro priority sample” thus corresponds to the specimen from the site with the highest assigned priority. For example, if both an omental metastasis and a primary tumor were available, the omental metastasis was designated as the priority sample; if only a primary tumor was available, it was considered the priority sample instead.

Accordingly, Fig. 1A now displays the distribution of priority samples (n = 66 total), consisting of omental metastases (n = 39), distant metastases (n = 12), adnexal/primary tumors (n = 8), and ascites samples (n = 7). We have revised the figure legend and restructured the panel to make this distinction clearer.

Action: We have revised Fig. 1A to improve clarity regarding the prioritization of sample sites and have included an additional description of this hierarchy in the updated figure legend (see below).

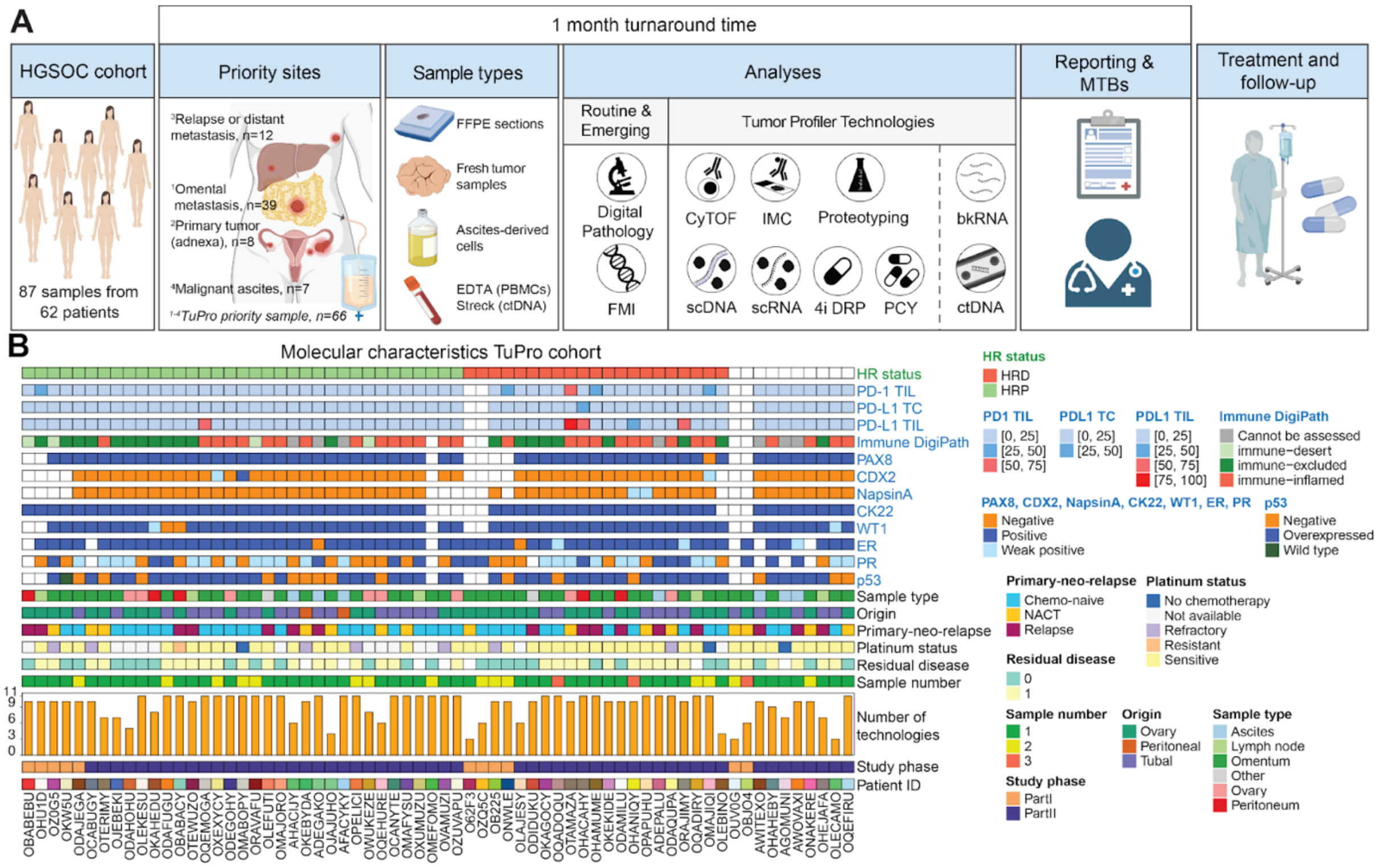

Figure 1. Ovarian cancer Tumor Profiler Cohort: Clinical cohort overview and molecular assessment using routine and emerging technologies.

A) Overview of the Swiss Tumor Profiler (TuPro) study design with cohort structure and applied technologies. Because some patients contributed multiple specimens from the same time point, a single priority sample was defined for each patient according to a pre-specified hierarchy: (1) omental metastasis, (2) other tissue (distant metastasis or primary tumor), and (3) ascites. **B)** Routine and emerging diagnostic features of HGSOC assessed by immunohistochemistry (blue row annotation) and targeted next-generation sequencing (green row annotation). White squares indicate missing data. Routine clinical and pathological characteristics are ordered by homologous recombination (HR) status (defined by pathogenic *BRCA1/BRCA2* variants and loss-of-heterozygosity (LOH) determined by FMI), followed by study phase (Part I: proof-of-concept and platform establishment; Part II: profiling phase for MTB recommendations). TuPro technology coverage (Number of technologies) for each sample is shown as a bar chart. See also Extended Data Fig. 1 to 3, Supplementary Table 1 to 2, Supplementary Data 1 to 11., Source data are provided as a Source Data file.

---

6. Regarding the statement, "This evaluation revealed that the majority of priority samples arose from patients carrying an immune-excluded or -inflamed TME, with a trend towards low PD-1 and PD-L1 expression," please comment on whether these features are representative of the broader HGSC population. Does this potential bias limit the generalizability of the findings?

Response: We thank the reviewer for raising this important point. The predominance of immune-excluded and -inflamed TMEs in our cohort reflects the biological and clinical characteristics of advanced-stage HGSOC, which typically presents with peritoneal or omental metastases—sites known to display heterogeneous yet frequently immune-excluded phenotypes. The relative enrichment of these TME types in

our dataset therefore mirrors the expected distribution in late-stage malignant disease rather than introducing a selection bias. Moreover, the majority of our samples were derived from metastatic sites in chemo-naïve patients, minimizing treatment-related immune alterations and preserving the intrinsic immune landscape. This is particularly relevant because previous studies have focused on primary ovarian tumors [4,5], despite more recently acknowledged biological and immunological differences between primary and metastatic sites in HGSOC [6–8]. However, we acknowledge that our cohort does not fully capture the entire immunological spectrum of HGSOC, particularly early-stage or HRD-positive tumors with higher immune infiltration [9].

We have now clarified this point in the Results section, emphasizing that while the immune composition observed here is representative for advanced HGSOC, the findings should be interpreted within this clinical context.

Action: We have revised the Results section to clarify representativeness and provide additional context: *"This predominance reflects the characteristic immune landscape of advanced-stage HGSOC, which frequently presents with omental and peritoneal metastases. As the majority of samples were derived from metastatic sites in advanced stage chemo-naïve patients with HGSOC, this likely preserved the intrinsic immune composition and minimized treatment-related alterations. This distinction is important, as previous studies have primarily analyzed primary ovarian tumors [4,5], although metastatic lesions in HGSOC are known to differ in their immune contexture [6–8]."*

We have also expanded the Discussion section to acknowledge the cohort scope: *"In regard to the cohort, its composition primarily represents advanced-stage, chemo-naïve HGSOC and therefore may not fully capture the immune diversity observed in early-stage or HRD-positive disease. Future interventional studies will aim to systematically compare immune phenotypes across primary and metastatic sites."*

---

7. What criteria did the Molecular Tumor Board (MTB) use to categorize treatment changes as "Very," "Largely," "Partially," or "Somewhat"? Was this assessment based on a structured questionnaire, Likert scale, or informal consensus? Please clarify the methodology used for these evaluations.

Response: The classification into "Very," "Largely," "Partially," and "Somewhat" useful was derived directly from a six-point Likert scale completed by participating clinicians after each MTB session. As described in the revised Methods section, this assessment reflected each clinician's individual perception of how informative the MTB discussion and molecular summary report were in shaping treatment decisions. No additional predefined criteria were applied, as the aim was to capture the subjective clinical judgment of MTB participants regarding the utility of integrated molecular and functional data.

Action: The methods section now included "**... individual perception…**" as stated below: "Following each MTB session, participating clinicians were asked to evaluate the usefulness of the MTB and affiliated

molecular summary report in supporting treatment recommendations. Responses were collected using a six-point Likert scale (Not useful, Barely useful, Somewhat useful, Partially useful, Largely useful, Very useful). These assessments reflected each clinician's **individual perception** of the contribution of the MTB to clinical decision-making, and were not guided by additional predefined criteria."

---

8. The authors note discrepancies between dissociation-based and tissue-based cell compositions, yet some conclusions (e.g., immune exclusion status, EMT markers) still rely on dissociated data. Will this difference introduce bias into the conclusions?

Response: We thank the reviewer for this important question. The comment may likely refer to two related but distinct aspects of our analysis:

(i) differences arising from technology type (FFPE tissue-based *versus* dissociation-based assays) and

(ii) biological variation between sample sources (solid tumor *versus* ascites).

Regarding the first, as shown in the current **Extended Data Fig. 6B**, we observed stronger correlations among single-cell suspension–based technologies (e.g., scRNA-seq, CyTOF, PCY) compared to tissue-based modalities applied to FFPE samples (IMC, FMI, DigiPath). These discrepancies may reflect inherent differences in cellular representation and preservation between fresh-dissociated and FFPE materials rather than analytical bias. It is important to note that the single-cell suspension and FFPE tissue were obtained as two distinct specimens from the same sampling site (ideally one tumor cut into two pieces). Slight variations in cell composition are therefore expected, as applied technologies require specific sample preparation and processing steps that do not permit perfect cross-comparison at single-cell resolution.

The second aspect refers to the biological variation, when comparing ascites and solid tumor samples, importantly, both of which were processed using an identical standardized enzymatic–mechanical dissociation protocol, we found that observed differences in cellular composition predominantly reflected biological heterogeneity. These were most pronounced within immune cell compartments, particularly in macrophage and T cell gene sets, whereas tumor cell profiles were largely consistent apart from EMT-related and diagnostic marker variation as shown in **Fig. 4** of the original manuscript.

Together, these observations suggest that the reported discrepancies arise from the intrinsic biology of the sampled compartments rather than methodological artifacts. Moreover, our multi-modal characterization of the tumor microenvironment further highlights the value of integrated tumor profiling, as treatment recommendations in molecular tumor boards are shaped by multiple complementary layers of evidence that would not be captured by any single technology alone. We have clarified this point in the revised text and emphasized the importance of considering sample type during MTB discussions, as the abundance of clinically relevant biomarkers may vary accordingly.

Action: We have added the following *"…This study also revealed that differences between profiling modalities mainly reflect the intrinsic biology of the sampled compartments rather than methodological artifacts. These findings emphasize the value of integrated multi-modal tumor profiling, as molecular tumor board recommendations are shaped by complementary layers of evidence that cannot be captured by a single technology alone…"*

---

9. The manuscript would benefit from a dedicated limitations section, addressing the observational nature of the study, potential batch effects between platforms, and technology-specific dropout or sample failures.

Response/ Action: We thank the reviewer for this helpful suggestion. We have now included a dedicated section highlighting limitations in the revised Discussion, which addresses the observational design of the study, potential batch effects between platforms, and technology-specific dropout or sample failures. This section has been added as described in our response to major comment 2 from Reviewer #1 (see above).

---

10. While datasets (except for public datasets) are not publicly available due to patient privacy, the analysis scripts are also not accessible. To improve reproducibility, please consider providing the analysis code or making it available upon request.

Response: We would like to take this opportunity to clarify that while the raw sequencing data are not available due to patient privacy concerns all of the derived data (such as gene / protein expression per cell / sample, drug responses, cell type compositions, mutations) will be made available to the public either as part of the supplementary data of this manuscript, or (for large files) via Zenodo. All of this data can also be accessed through the TumorProfiler data resource website, which we now added to the data availability statement:

"In addition, all publicly accessible data is available through the Tumor Profiler data page on https://www.tumorprofiler.com/resources/ovarian. Access is currently restricted to reviewers with username reviewer and password _tupro_ovca_239, but will be released upon publication of this manuscript."

We would also be happy to provide the code used to generate the figures on request, and have added this information in a separate Code Availability section.

Action: We have updated the data availability statement and added a code availability statement.

Reviewer #2 (Remarks to the Author)

I co-reviewed this manuscript with one of the reviewers who provided the listed reports. This is part of the Nature Communications initiative to facilitate training in peer review and to provide appropriate recognition for Early Career Researchers who co-review manuscripts.

Reviewer #3 (Remarks to the Author)

This manuscript reports the findings of the Tumor Profiler observational trial, in which samples from 62 HGSOC patients were extensively profiled using multiple methodologies, with almost 75% of the priority samples undergoing analysis by at least 9 of 11 technologies. The authors have done an excellent job in placing their findings in the context of the current HGSOC profiling literature and adding interesting new data particularly on whole genome amplification and tumor microenvironment changes. Overall, the manuscript is well-written.

Response: We thank the reviewer for the positive and encouraging feedback. We are pleased that the reviewer found the manuscript well-written and recognized the value of our comprehensive multi-technology profiling and its contribution to understanding genomic alterations and tumor microenvironmental changes in HGSOC.

The manuscript would be strengthened by addressing a number of specific additional areas as follows.

Major:

1. It is unclear how the initial patient cohort was diagnosed with HGSOC. By conventional H+E pathology? Was there central review and/or re-review prior to analysis? The data shown in Figure 1B suggest a relatively large subset of cases for which none of PAX8, WT1, and p53 data are available. How was the diagnosis made in these cases, given these are frequent IHC methods for diagnosis of HGSOC?

Response: All cases included in this study were diagnosed as high-grade serous ovarian carcinoma (HGSOC) based on conventional hematoxylin and eosin (H&E) histopathology, following WHO diagnostic criteria. Briefly, the diagnosis was supported by characteristic histologic features, including solid, papillary, glandular, or cribriform growth patterns composed of columnar to cuboidal epithelial cells with marked nuclear atypia and pleomorphism, prominent nucleoli, and a high mitotic index. Necrosis and psammoma bodies were variably present, and serous tubal intraepithelial carcinoma (STIC) was identified in the fimbrial region in selected cases. Representative slides were centrally reviewed by expert gynecopathologists prior to inclusion.

In all patients, diagnosis was established through surgical resection and histopathologic examination of resected tissue, supported by contrast-enhanced computed tomography and ultrasound and serum CA125

as a tumor marker. Immunohistochemical confirmation using WT1, PAX8, and p53 was available for the majority of cases and supported the HGSOC diagnosis. For a small subset lacking IHC data, diagnosis was made by expert morphological assessment in the context of clinical presentation, imaging findings, and tumor marker profile, consistent with established diagnostic standards. We have also revisited all pathology reports and incorporated the missing IHC information into **Fig. 1B**.

Action: Fig. 1B has been updated. In addition, we have added "All patients were diagnosed with high-grade serous ovarian carcinoma (HGSOC) based on histopathologic examination of surgically resected tissue, following WHO diagnostic criteria. Diagnosis was supported by contrast-enhanced computed tomography (CT) as the preferred imaging modality and serum CA125 as a tumor marker. Representative slides were centrally reviewed by expert gynecopathologists and characterized by solid, papillary, glandular, or cribriform growth patterns with marked nuclear atypia, pleomorphism, and high mitotic activity. Immunohistochemical staining for WT1, PAX8, and p53 was performed in most cases to confirm the diagnosis." to the material and methods section.

---

2. The authors highlight that one of the major areas of impact of their work is the potential change in clinical management from multi-omics findings that extend beyond just genomic assessments. Please provide the actual changes in treatment recommendation provided by TuPro, as this information is necessary to evaluate the true clinical impact as suggested by the authors.

Response/ Action: TuPro was an observational study, and therefore no established treatment guidelines—such as first-line chemotherapy—were altered on the basis of TuPro data. However, TuPro findings were considered by treating physicians when selecting maintenance therapy, including the addition of letrozole, metformin, or bevacizumab. The specific ways in which TuPro influenced treatment decisions are now illustrated for four representative cases in **Fig. 2E**, and the complete decision history for all patients has been added to **Supplementary Table 12**.

We agree that the observational design limits the ability to draw firm conclusions regarding the clinical benefit of multi-omics–guided precision medicine. To address this limitation, we are now conducting a prospective, randomized interventional trial evaluating multi-omics–guided treatment versus routine clinical management in HRD-negative HGSOC (OVPrecision, NCT06466382). This information can be found in the discussion of the revised manuscript.

The concern raised by Reviewer 3 has been addressed in detail in our response to Reviewer 1, Comment 1. We have updated **Fig. 2** and the corresponding Results text accordingly. We hope that these additions and clarifications fully resolve the reviewer's concerns.

---

3. Figure 2. This is a mix of both biomarkers and drug response assays (drug response profiling and pharmacoscopy). The relative contribution of these different sets of data to treatment decisions is hard to

tease out as shown, and would be clearer if the molecular and drug screening assays were separated out. Of the two categories, which is more commonly driving treatment changes?

Response: We thank the reviewer for this important suggestion. In the revised version, we have updated **Figure 2A** to more clearly distinguish between molecular biomarkers and *ex vivo* drug response data. Specifically, the figure now separates treatment-influencing features based on their data origin (molecular profiling vs. functional drug sensitivity assays).

Regarding the second question referring to frequent use of both "categories": both molecular markers and *ex vivo* drug response data (4iDRP and PCY) influenced treatment recommendations, often in a complementary fashion. Higher confidence in a treatment recommendation was typically achieved when multiple, orthogonal data types supported the same therapeutic mechanism. We have illustrated this with four selected case studies in a new **Figure 2E**. Importantly, some treatment suggestions were uniquely driven by either molecular biomarkers (e.g., *BRCA1/2*, LOH, pathway activation markers) or by *ex vivo* response (e.g., PARPi sensitivity or resistance), depending on data availability and the nature of the drug. For example, standard treatments like the combination of carboplatin and paclitaxel or carboplatin and olaparib lack established predictive biomarkers and were thus informed primarily through either one or both functional assays. Conversely, not all targeted agents were included in the *ex vivo* panels, in which case molecular data served as the basis for recommendations. We would also like to emphasize that the Tumor Profiler study was a non-interventional observational trial; thus, first-line therapy could not be modified based on MTB recommendations. These aspects are now clarified in the revised text and figure legend.

Action: We have modified Figure 2 accordingly (see below). The revised figure, corresponding legend, and results section are provided above in our response to comment 1 from Reviewer #1.

Minor:

4. A number of box-and-whisker plots lack y-axis labels, e.g., Figure 7A right panel, Figure 6H top panel. Please check throughout.

Response/ Action: We thank the reviewer for this careful observation. We have systematically reviewed all figures and added or clarified missing y-axis labels throughout the manuscript slightly enlarged and standardized the axis annotations in the revised figure to ensure they are easily visible in both print and digital formats, including Figures 3A, 5C, 6G–H, 7A, 7C–D, S5B, S7H, 8E–F, 9C, 10A–C, 11A, 11D, 12A, and 12C. Labeling was adjusted where necessary to ensure consistency and interpretability (e.g., replacing "frequency" with "cell type abundance" in Extended Data Fig. 7H and improving UMAP/tSNE scale definitions).

---

5. Please clarify how the pH2AX biomarker influenced treatment decisions.

Response: We thank the reviewer for this comment. The rationale for using γH2AX (phospho-H2AX Ser139) is based on the well-established principle that DNA double-strand breaks induce phosphorylation of histone H2AX in the chromatin flanking the break site; antibodies directed against γH2AX can therefore be used to monitor endogenous or treatment-induced DNA damage [10]. Consistent with its potential role as a surrogate marker, previous studies have reported γH2AX as a predictive or pharmacodynamic biomarker in various contexts, including acute myeloid leukemia [11–13], circulating tumor cells following PARP inhibitor or topotecan exposure [14], and in HGSOC ascites-derived primary cell cultures showing differential HR pathway activity in PARPi-sensitive and -resistant cells [15].

Accordingly, we assessed γH2AX as a surrogate of endogenous DNA damage. In mTB discussions, γH2AX was not used as a stand-alone biomarker, but as supportive evidence alongside HRD status and other potential molecular and functional readouts. Specifically, elevated baseline γH2AX reinforced recommendations for DNA damage response (DDR)–targeting regimens (e.g., platinum and/or PARP inhibitors) when concordant with HRD positivity (defined by BRCA1/2 alterations or elevated LOH). Conversely, low γH2AX in HRD-negative tumors did not preclude DDR therapy but deprioritized it in the absence of other supporting signals (e.g., *ex vivo* platinum sensitivity, additional genomic markers).

Taken together, high basal γH2AX likely reflects defective DNA repair or replication stress, supporting its role as orthogonal, confidence-boosting evidence rather than a determinant by itself. We therefore included γH2AX as a potential surrogate marker relevant for therapies interfering with DNA damage repair. No changes were required in the main text, as this clarification is now provided here.

Reviewer #4

This paper presents findings from the Tumor Profiler (TuPro) observational study, a comprehensive multi-omic profiling initiative for patients with high-grade serous ovarian cancer (HGSOC). The authors applied up to 11 high-resolution DNA, RNA, protein, and functional platforms on samples from 62 patients, integrating single-cell, spatial, and ex vivo drug-response data. A key innovation is the delivery of clinically actionable data within a 4-week turnaround, used to guide hypothetical treatment decisions via molecular tumor boards (MTBs). The study highlights how chemotherapy alters cancer cell heterogeneity, and identifies whole-genome amplification (WGA) as a poor prognostic factor.

Detailed comments:

1. The authors state that “In 21 out of 39 cases (54%) where TuPro altered the treatment decision, this change was also reflected in the physician’s final treatment choice.” Would it be possible to compare clinical outcomes between patients whose treatments aligned with the TuPro recommendations and those whose did not? Even an exploratory retrospective analysis could provide valuable insight into the clinical impact of implementing TuPro-guided decisions and would help strengthen the study’s claims regarding clinical relevance. Also, it would be interesting to see whether the effect of TuPro-informed

treatment decisions on clinical outcomes differs by molecular subgroup (e.g., WGA vs. non-WGA, HRD vs. HRP).

Response: We thank the reviewer for this suggestion. As also requested by reviewer #1, we have now conducted a comparison of overall survival (OS) in newly diagnosed patients where TuPro affected the real-life treatment *versus* those where it did not (new **Fig. 2**, see response to comment 1 made by reviewer 1). While this analysis was limited by the number of patients and the study design, the results highlighted improved OS for patients where the TuPro influenced the treatment.

---

2. The paper notes that while TuPro recommendations influenced treatment decisions in 76% of cases, most changes were related to maintenance therapy (e.g., addition of letrozole, bevacizumab, or PARPi), and not to first-line (neo)adjuvant chemotherapy, which was generally administered per standard of care. This distinction is important because it raises the question of how early in the treatment course TuPro data can have actionable value. Can the authors clarify:

• **What proportion of patients received a TuPro-guided change to frontline therapy versus maintenance therapy?**

Response/Action: We thank the reviewer for this important question. The Tumor Profiler Ovarian Cancer study was designed as an observational clinical study, with the primary goal of assessing the feasibility and clinical utility of multi-omics tumor profiling in guiding decision-making. Treating physicians had access to TuPro data for all enrolled patients, but no randomization or mandated treatment modification was implemented. Final therapeutic decisions remained fully at the discretion of the treating physicians and within current clinical guidelines. Consequently, no TuPro-guided treatment changes were made in the frontline setting, as it would not have been ethically permissible to alter first-line chemotherapy in newly diagnosed patients with HGSOC. Instead, TuPro-guided adaptations occurred exclusively in the maintenance setting for 21 patients (see modified **Fig. 2**, feedback to comment 1 raised by reviewer 1) or in relapsed patients. However, to prospectively evaluate the clinical impact of TuPro-guided treatment in the frontline setting, we have now initiated the randomized clinical trial OV Precision (NCT06466382), a window-of-opportunity phase IIb study testing multi-omics–guided therapy administered for up to two cycles (42 days) prior to interval debulking surgery. Additional information have been added to the Results chapter “Multi-omics tumor profiling alters treatment recommendations.”

• **Whether the timing of TuPro recommendations (e.g., pre- vs. post-surgery) allowed meaningful integration into clinical decision-making?**

Response: The TuPro results were available within 4 weeks of sampling for all patients discussed at the MTB. This is in line with the turnaround time for other comprehensive tests like FoundationOne, and thus it could be incorporated into clinical routine. Because the TuPro only influenced the real-life choice of maintenance therapy, and not the selection of the front line chemotherapy, it was equally applicable to

chemo-naive patients and patients after neo-adjuvant chemotherapy. Indeed, the fraction of patients where the Tupro influenced the hypothetical or real treatment choice was independent of treatment status (see Figure 2C).

• **If there were any measurable short-term clinical effects (e.g., delayed progression, biomarker responses, fewer adverse events) in patients receiving TuPro-guided maintenance therapy?**

Response/ Action: We thank the reviewer for this important question. As detailed in our response to comment 1 from Reviewer #1 (see above), we have now performed a comparison of overall survival (OS) in non-relapse patients in whom TuPro profiling influenced maintenance therapy versus those in whom it did not (new **Fig. 2E**). In addition, we have included four representative cases illustrating how TuPro data informed treatment modifications (new **Fig. 2F**). The reviewer is correct that these TuPro-guided adaptations primarily occurred in the maintenance setting of newly diagnosed HGSOC patients, where multi-omics insights supported the selection or substitution of targeted therapies.

---

3. The paper presents several notable associations, such as the link between whole genome amplification (WGA) and earlier disease recurrence, and suggests that TuPro profiling offers added value beyond standard genomic tests like FoundationOne CDx. However, the paper does not present formal statistical models to evaluate how the integration of TuPro data improves prognostic or predictive accuracy. Have the authors considered multivariate survival models (e.g., Cox regression) that include clinical variables, standard genomic, and TuPro-derived new variables to quantify the incremental prognostic value of TuPro? Reporting model performance metrics such as the concordance index (C-index), Akaike Information Criterion (AIC), or likelihood ratio tests would strengthen this claim.

Response/ Action: We thank the reviewer for this valuable suggestion. A Cox proportional hazards model incorporating key clinical variables together with whole genome amplification (WGA) status is already presented in **Fig. 3C**, demonstrating its association with earlier disease recurrence. In addition, we have now incorporated outcome analyses including likelihood ratio statistics related to treatment changes in the maintenance setting (as described in our response to comment 1 from Reviewer #1). These analyses further support the prognostic relevance of TuPro-derived parameters when considered alongside standard clinical and genomic variables.

We acknowledge, however, that the statistical power of the current study is limited by the sample size (62 patients/sampling time points) and the observational design. To address this limitation, we have initiated the prospective, randomized OV Precision trial (NCT06466382), which will formally evaluate the clinical and prognostic impact of TuPro-guided treatment recommendations in a larger patient cohort. The currently recruiting and running trial has been mentioned in the discussion as an outlook.

---

4. The authors report that CN signatures 4 and 7 are enriched in samples with whole genome amplification (WGA), and that these signatures are associated with focal amplifications and genome doubling, respectively. However, the clinical implications of these findings remain unclear. Can the authors elaborate on whether these CN signatures provide predictive value for treatment response, beyond their descriptive association with WGA and genomic instability?

Response/ Action: We thank the reviewer for this insightful comment. The clinical relevance of WGA and CN signatures 4 and 7 is discussed in detail in the original manuscript. As shown in our analyses, WGA is significantly associated with poor outcome, consistent with previous reports linking whole-genome duplication events to increased chromosomal instability and adverse prognosis across several tumor types. In our cohort, both CN signatures 4 and 7—representing focal amplification and genome duplication processes, respectively—were predominantly observed in post-NACT and relapse samples (**Fig. 6K**), suggesting that these patterns reflect therapy-induced genomic evolution and instability rather than treatment sensitivity per se. Furthermore, our data indicate that chemotherapy exposure in *TP53*-mutated tumors promotes the emergence of WGA and altered CN signatures, which may contribute to disease progression through increased genomic complexity and reduced genomic integrity. This is consistent with prior studies associating these signatures with aggressive clinical behavior and reduced progression-free survival in late-stage HGSOC [16,17].

Together, these findings imply that CN signatures 4 and 7 serve as biological indicators of genomic instability and disease evolution, rather than direct predictors of treatment response. We believe this interpretation—supported by both our data and published literature—is adequately addressed in the Results and Discussion sections of the revised manuscript.

---

5. While the study shows modest differences between solid tumor and ascites samples, only a limited number of matched samples were profiled across all modalities. Can the authors specify the n for paired comparisons per platform? Similarly, for patients with longitudinal samples, can the authors provide more detail (e.g., a table) describing temporal changes in molecular features or treatment outcomes?

Response: Due to resource constraints, only CyTOF and PCY profiled matched ascites and solid tissue samples from the same patient at the same time point (n = 7 sample pairs profiled by CyTOF, n = 6 sample pairs profiled by PCY, see **Figures 4E** and **Extended Data Fig. 7G**). Our data suggest that inter-patient variability exceeds subtle differences between sampling sites. Hence, a different study design focusing on matched sample pairs would be necessary to further investigate the differences between ascites and solid tumor samples.

There were a total of 4 longitudinal patient cases (see table below). For two of these cases, one of two time points was only analysed by very few technologies, and for one additional case, the second time point (ascites) sample had very low tumor content, limiting our ability to systematically assess changes in multi-omics profiles over time.

Rebuttal table 1: Overview of patients with longitudinal samples, time point 1 (t_1), time point 2 (t_2).

Patient ID	Sample IDs t_1	Sample IDs t_2	Profiling technologies t_1	Profiling technologies t_2	Notes
TP-G2-USB-041	OLECAMO	AHACIJY	CyTOF, IMC, DigiPath	CyTOF, PCY, IMC, FMI, DigiPath	
TP-G2-USB-043	OJAJUHO	ADEGAKO	CyTOF, FMI, DigiPath	All	
TP-G1-USB-013	ODAJEGA, AFUFAGU	AWITEXO	All except scDNA	All except cfDNA	Very low tumor content in ascites sample at TP2
TP-G2-USB-004	ODAFUGU, OCIMOLY	AWOVAXI	All	All except FMI	

---

6. The paper suggests that TuPro data could guide frontline treatment de-escalation or substitution, yet the current cohort is largely retrospective and observational. Are there plans for a prospective interventional trial specifically testing TuPro-guided first-line therapy?

Response/ Action: Yes. Building on the results and experience gained from the TuPro study, we have initiated a prospective interventional "window-of-opportunity" trial investigating the benefit of multi-omics–guided neoadjuvant treatment in HRD-negative (HRP) high-grade serous ovarian cancer (HGSOC) patients (OVPrecision, NCT06466382). This information can be found at the end of the discussion: "... Similar shifts in treatment strategy have already been applied in breast cancer, where de-escalation trials are exploring the use of targeted therapies instead of chemotherapy at the time of diagnosis (e.g. WSG-ADAPT trial, NCT01779206). Our trial supports that (neo-) adjuvant treatment in HGSOC should be guided by the tumor's molecular profile, potentially favoring personalized molecularly guided targeted therapies over traditional chemotherapy. While this hypothesis is supported by the extensive data generated by our analysis, we acknowledge that further evidence from interventional clinical trials is needed to validate this approach. To further investigate this, we are currently enrolling patients in the prospectively randomized clinical OV Precision Trial (Swiss-GO Trial Group/ Tumor Profiler Center, NCT06466382). This trial aims to provide early insights into the feasibility and patient outcome of multi-modal, MTB-driven treatment guidance in clinical practice for newly diagnosed ovarian cancer patients…"

REFERENCES

1. Uppaluri, R. et al. Biomarker and Tumor Responses of Oral Cavity Squamous Cell Carcinoma to Trametinib: A Phase II Neoadjuvant Window-of-Opportunity Clinical Trial. Clin. Cancer Res. 23, 2186–2194 (2017).

2. Gilmartin, A. G. et al. GSK1120212 (JTP-74057) is an inhibitor of MEK activity and activation with favorable pharmacokinetic properties for sustained in vivo pathway inhibition. Clin. Cancer Res. 17, 989–1000 (2011).

3. Nougarede, L., Hazane-Puch, F., de Fraipont, F., Jacquet, E. & Bidart, M. BRCA1 promoter methylation predicts PARPi response in ovarian cancer: insights from the KOMET study. Clin. Epigenetics 17, 140 (2025).

4. Konecny, G. E. et al. Prognostic and therapeutic relevance of molecular subtypes in high-grade serous ovarian cancer. J Natl Cancer Inst 106, (2014).

5. Cancer Genome Atlas Research Network. Integrated genomic analyses of ovarian carcinoma. Nature 474, 609–615 (2011).

6. Lanickova, T. et al. Chemotherapy Drives Tertiary Lymphoid Structures That Correlate with ICI-Responsive TCF1+CD8+ T Cells in Metastatic Ovarian Cancer. Clin. Cancer Res. 31, 164–180 (2025).

7. Vázquez-García, I. et al. Ovarian cancer mutational processes drive site-specific immune evasion. Nature 612, 778–786 (2022).

8. Stur, E. et al. The dynamic immune behavior of primary and metastatic ovarian carcinoma. NPJ Precis. Oncol. 9, 120 (2025).

9. Mikulak, J. et al. Immune evasion mechanisms in early-stage I high-grade serous ovarian carcinoma: insights into regulatory T cell dynamics. Cell Death Dis. 16, 229 (2025).

10. Redon, C. E. et al. Histone H2AX and Poly(ADP-Ribose) as Clinical Pharmacodynamic Biomarkers. Clin. Cancer Res. 16, 4532–4542 (2010).

11. Fong, P. C. et al. Inhibition of poly(ADP-ribose) polymerase in tumors from BRCA mutation carriers. N. Engl. J. Med. 361, 123–134 (2009).

12. Pikkusaari, S. et al. Functional Homologous Recombination Assay on FFPE Specimens of Advanced High-Grade Serous Ovarian Cancer Predicts Clinical Outcomes. Clin. Cancer Res. 29, 3110–3123 (2023).

13. Faraoni, I. et al. BRCA1, PARP1 and γH2AX in acute myeloid leukemia: Role as biomarkers of response to the PARP inhibitor olaparib. Biochim. Biophys. Acta 1852, 462–472 (2015).

14. Wang, L. H. et al. Monitoring drug-induced gammaH2AX as a pharmacodynamic biomarker in individual circulating tumor cells. Clin. Cancer Res. 16, 1073–1084 (2010).

15. Sheta, R. et al. Development of a 3D functional assay and identification of biomarkers, predictive for response of high-grade serous ovarian cancer (HGSOC) patients to poly-ADP ribose polymerase inhibitors (PARPis): targeted therapy. J. Transl. Med. 18, 439 (2020).

16. Macintyre, G. et al. Copy number signatures and mutational processes in ovarian carcinoma. Nat. Genet. 50, 1262–1270 (2018).

17. Watkins, T. B. K. et al. Pervasive chromosomal instability and karyotype order in tumour evolution. Nature 587, 126–132 (2020).

We thank the reviewers for their constructive feedback on our manuscript "*A tumor profiling resource for ovarian cancer: insights into chemotherapy-driven heterogeneity and personalized treatment strategy*" (**NCOMMS-25-18573A)**, which we believe has been substantially improved based on their comments. Below, we provide our responses to the remaining points raised during the revision round. All changes made in the revised manuscript are highlighted using track changes. We hope that we have comprehensively addressed all comments and thank the reviewers for their careful evaluation of our work.

Reviewer feedback:

Reviewer #1 (Remarks to the Author)

The authors have mostly answered my comments. The only comment remaining is that the word integration is used interchangeably to incorporation and correlation. In their rebuttal letter they refer to integration, but finally the MOFA analysis did not reveal anything and was not added to the manuscript, and our comment on integration did not result in any change in the manuscript. As real integration of Multi-omics data is not presented in the manuscript, this should also be added to limitations in the discussion.

Response: We thank the reviewer for this important clarification. We agree that the term integration can be interpreted inconsistently and that, in parts of the manuscript and rebuttal, it may have been used too broadly to describe cross-modal incorporation and correlation rather than a fully unified computational integration framework.

Although we explored unsupervised multi-omics integration using MOFA during the first round of revision, this analysis did not yield stable or biologically robust patient groupings and was therefore not included in the revised manuscript, but is presented in the rebuttal letter only (**Rebuttal Figure 1**). Accordingly, the current study does not present a single formal multi-omics integration model. Instead, our analyses rely on coordinated, modality-aware interpretation of complementary datasets within a molecular tumor board setting. The results of the exploratory MOFA analysis shown in the rebuttal letter will be made available to the community alongside the manuscript.

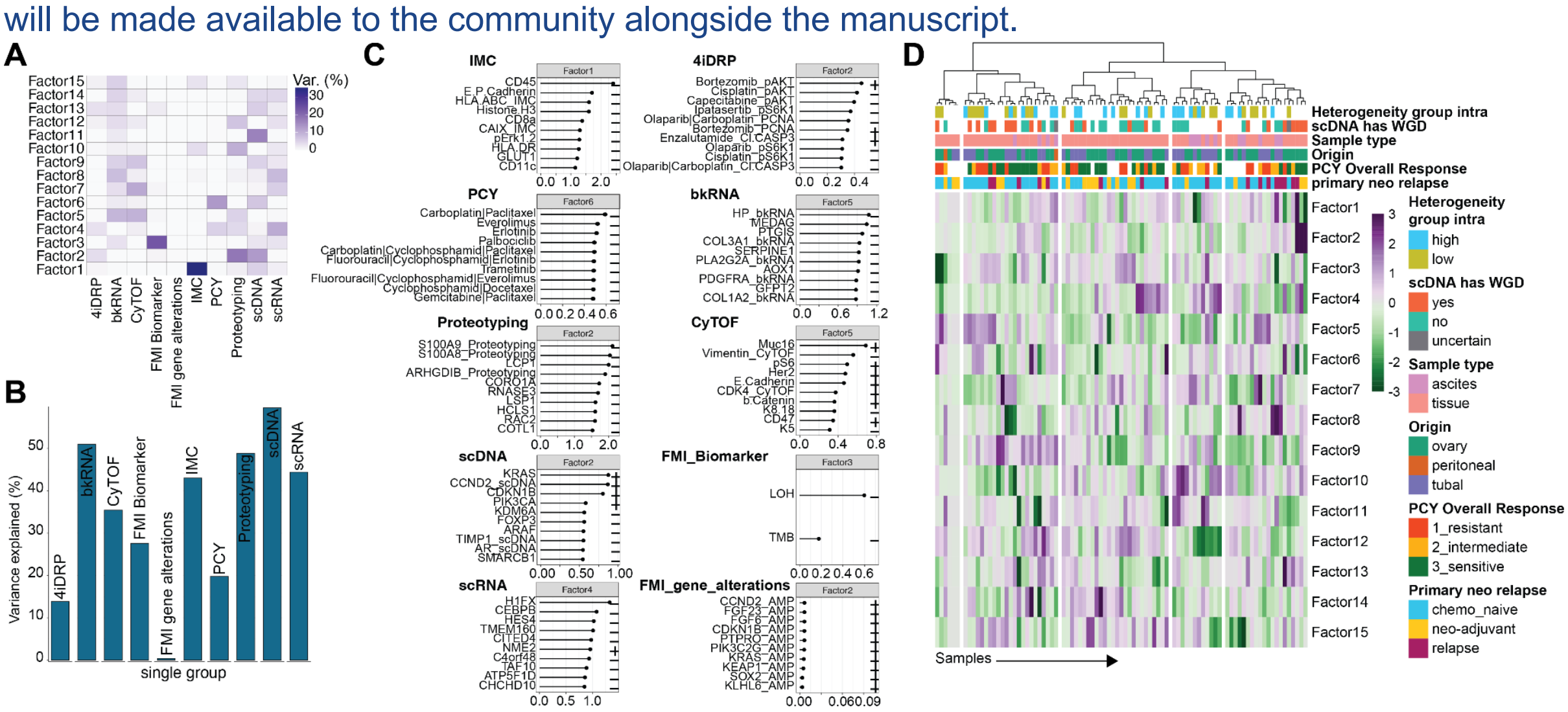

Rebuttal Figure 1. Exploratory Multi-Omics Factor Analysis (MOFA) showing shared and assay-specific sources of variation across the TuPro HGSOC cohort. **A)** Proportion of total variance explained (R^2) by each factor (rows) across the different omics layers (columns) included in the model. Each column represents an assay (4iDRP, bulk RNA-seq, CyTOF, IMC, proteotyping, scDNA-seq, scRNA-seq, Foundation Medicine gene alterations, and associated biomarkers). **B)** Cumulative variance explained by each single omics group, illustrating the relative contribution of each data modality to the global model. **C)** Exemplary top factors and their strongest molecular feature loadings per TuPro technology. Shown are absolute loading values for the genes, proteins, or markers with the highest weights. Plus (+) or minus (–) signs at the right side of the plot indicate the direction of the loading. **D)** Heatmap visualization of the top 15 factors derived from MOFA, with samples (columns) hierarchically clustered (euclidean distance, complete linkage) based on their factor values (rows). Annotations on top indicate key clinical and molecular features, including intratumoral heterogeneity group, WGD (whole-genome duplication) status from scDNA-seq, sample type, tumor origin, ex vivo drug response (PCY overall response), and clinical treatment setting (chemo-naïve, neoadjuvant, or relapse). Together, the analysis highlights both shared and modality-specific latent factors capturing major axes of molecular and phenotypic heterogeneity in the TuPro HGSOC cohort.

Action: To address the reviewer's comment explicitly, we have revised the Discussion and added a statement reflecting limitations (Nature communications do not have an explicit Limitation section) clarifying that full algorithmic integration of all data modalities was not achieved in this study. We now

emphasize that the work focuses on structured cross-modal interpretation rather than unified latent-variable modeling, and that the development of robust multi-omics integration frameworks remains an important objective for future studies. We have written the following: *"The applied technologies—implemented here in unprecedented breadth—characterized tumor cell composition together with genetic, transcriptomic, and proteomic features, as well as functional ex vivo drug responses. While multiple data modalities were analyzed in parallel, the study does not present a single unified, unsupervised multi-omics integration model, but instead relies on coordinated, modality-aware interpretation of complementary datasets. Although technologically comprehensive…"*

The word integration should be checked across the manuscript and changed. E.g.
Row 153 - Integration of a clinically implemented platform - do you mean application or usage? Discussion, first chapter, and Line 470 Integration of multi-omics data - It is actually incorporation.
Response/Action: In both cases, "integration" has been replaced with "incorporation". Additional changes were made as highlighted in the revised track changed manuscript submitted along with a clean version.

In Fig legend 6 F - Integration is more like a correlation? (shown in G).
Response/Action: We have revised the legend of Figure 6F, it now reads "*Comparison of differential pathway activation between chemo-naive and NACT was conducted separately in bulk and scRNA-seq. Each dot corresponds to a pathway, the values shown are the t-statistics obtained from a linear regression model. Positive values indicate increased pathway scores in samples taken after NACT.* "

Line 436 - inter-sample integration - what do you mean by this integration - likely not the right word for this. Intermixing? Please clarify.
Response/Action: We thank the reviewer for this clarification. We agree that the term *"integration"* was imprecise and have replaced it with *"inter-sample mixing"* to more accurately describe the observed overlap of T and myeloid cell clusters across samples in Fig. 7B, avoiding confusion with formal data integration methods..

Reviewer #2 (Remarks to the Author)

I co-reviewed this manuscript with one of the reviewers who provided the listed reports. This is part of the Nature Communications initiative to facilitate training in peer review and to provide appropriate recognition for Early Career Researchers who co-review manuscripts.
Response/Action: We thank Reviewer #2 for their contribution to the review process.

Reviewer #3 (Remarks to the Author)

Thank you for thoroughly addressing the prior feedback, which has strengthened the manuscript.
Response/Action: We thank Reviewer #3 for the positive feedback and for acknowledging that the revisions have strengthened the manuscript.

Reviewer #4 (Remarks to the Author)

The authors have provided thoughtful responses, and the addition of survival analyses together with clarification regarding the timing of TuPro-guided decisions meaningfully strengthens the manuscript. However, several issues related to the interpretation of clinical benefit and the incremental value of TuPro remain insufficiently addressed. My follow-up comments are below.

1. The newly added Kaplan–Meier analysis suggests improved overall survival among newly diagnosed patients whose treatment was influenced by TuPro recommendations (log-rank p = 0.026; HR = 0.36 [0.14–0.91]). While encouraging, additional clarification is needed to assess robustness.

Please clarify the analytic population and time origin for the OS analysis in Fig. 2E. Was OS measured from diagnosis, treatment initiation, or the start of maintenance? Because TuPro recommendations primarily affected

maintenance therapy, defining time-zero earlier than maintenance may introduce immortal-time bias. If OS was anchored earlier, a landmark or time-dependent Cox analysis would help mitigate this concern.

Response/ Action: We have added further details on survival analysis to the Material and Methods section. "*Survival analysis - Relapse-free survival was defined as the time from initial diagnosis to documented disease recurrence or death, whichever occurred first. Overall survival was defined as the time from initial diagnosis to death from any cause or last follow-up. All survival analyses were performed using the Cox proportional hazards model as implemented in the coxph function in the survival R package (version 3.8-3). Results were visualized using survminer (version 0.5.0)*."

The manuscript presents an adjusted Cox model for recurrence-free survival, but the OS analysis in Fig. 2E appears unadjusted. If feasible, please report an adjusted OS model including key prognostic covariates (e.g., stage, residual disease, BRCA/HRD status, age, ECOG). Even exploratory estimates would help determine whether the association persists after accounting for baseline differences.

Response: We thank the reviewer for this suggestion. We have now performed additional analysis using an adjusted Cox model including the following potential confounders: FIGO stage (III/IV, note that we now excluded one patient with FIGO stage I from all survival analyses), residual disease (0/1), HR status (based on FoundationOne loss-of-heterozygosity), and age range (4 groups). ECOG was not collected in this study, however, the inclusion criteria required an ECOG of 2 or lower.

Using this adjusted model, we obtained a hazard ratio of 0.27 (95% CI: 0.064–1.1; p = 0.07) for patients receiving TuPro-altered real-life treatment. The corresponding forest plot has been added to the **Extended Data Figure 4B**. Although this association does not reach conventional statistical significance, the observed effect size is substantial (HR=0.27), corresponding to a ~73% reduction in hazard. The confidence interval narrowly crosses unity, and given the limited number of events (n = 19) and the observational nature of our clinical study, the analysis is likely underpowered to detect effects of this magnitude with conventional significance thresholds. We therefore interpret this finding as a biologically and clinically meaningful trend rather than a definitive negative result.
The data to perform this analysis as well as all the obtained coefficients are now included in the Source Data, and the details of this analysis were added to the methods section.

Action: Additional findings have been added to the manuscript and related files: "*These alterations were involving the choice and type of PARPi, or the addition of letrozole, metformin or bevacizumab (Fig. 2E and 2F, see Supplementary Data 12 for details). In the group of newly diagnosed patients (chemo-naïve or post-NACT, FIGO stages III or IV) this maintenance alteration led to a significant overall survival benefit in patients compared to patients where TuPro did not alter the treatment (unadjusted Cox model, log-rank p=0.007; HR=0.30 [0.12-0.75], Cox model adjusting for age, FIGO Stage, residual disease, LOH and treatment status p=0.07, HR=0.27 [0.064-1.12]); Fig. 2E and Extended Data Fig. 4B)*".

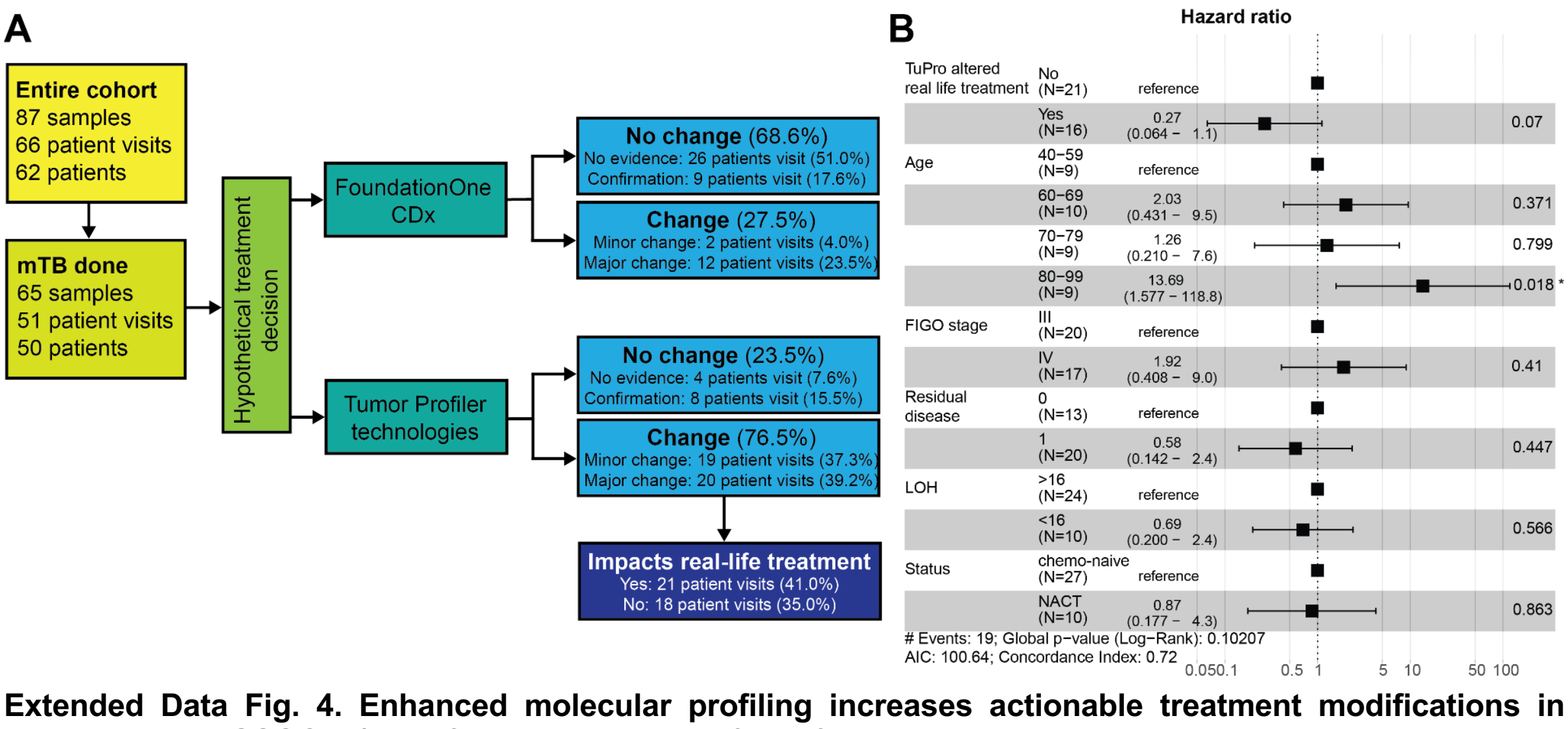

Extended Data Fig. 4. Enhanced molecular profiling increases actionable treatment modifications in patients with HGSOC. A) The figure illustrates the flow of patient samples analyzed and presented to the mTB summarizing the impact of molecular profiling on hypothetical treatment decisions. A total of 87 samples from 62 patients were included in the cohort, with 65 samples from 50 patients undergoing molecular tumor board (mTB)

analysis. **B)** Multivariable Cox proportional hazards regression analysis of overall survival in newly diagnosed HGSOC patients. Hazard ratios (HRs) and 95% confidence intervals are shown for TuPro-altered real-life treatment and clinical covariates, including age, FIGO stage, residual disease, loss of heterozygosity (LOH), and treatment status (chemo-naïve versus post-NACT). The reference category is indicated for each variable. The vertical dashed line denotes HR = 1. Model performance metrics are shown below the plot (number of events, global log-rank p-value, Akaike information criterion (AIC), and concordance index).

---

2. In the rebuttal, the authors note that likelihood-ratio statistics were incorporated for outcome analyses related to treatment changes to evaluate the incremental prognostic value of TuPro. Could you clarify where these LR statistics are presented in the manuscript? From the text and figures, the only reported outcome comparisons appear to be log-rank tests (Fig. 2E), and I did not identify likelihood-ratio tests comparing nested Cox models. If LR analyses were performed, please specify where they are reported and describe the models being compared.

Response/Action: We thank the reviewer for pointing out this inconsistency and appreciate the opportunity to clarify. The reference to “likelihood-ratio statistics” in our previous rebuttal was imprecise. In the revised manuscript, outcome comparisons related to TuPro-informed treatment changes are based on Kaplan–Meier analyses with log-rank tests, as shown in **Fig. 2E**. These log-rank tests are mathematically equivalent to a Cox proportional hazards model including a single categorical predictor (TuPro-altered *versus* non-altered treatment), but we did not perform or report formal likelihood-ratio tests comparing nested multivariable Cox models with and without TuPro-derived covariates. Given the limited sample size and number of events, we deliberately refrained from overfitting multivariable models, and instead present these results as exploratory, hypothesis-generating evidence supporting further prospective evaluation of TuPro-guided treatment strategies.

Reviewer feedback:

We thank the reviewers for their constructive feedback on our manuscript, which we believe has been substantially improved based on their comments. Below, we provide our responses to the remaining points raised during the revision round. All changes made in the revised manuscript are highlighted using track changes. We hope that we have comprehensively addressed all comments and thank the reviewers for their careful evaluation of our work.

Reviewer #1 (Remarks to the Author)

The authors have now addressed my final comment.

Response/Action: We thank Reviewer #1 for constructive feedback throughout the revision process.

Reviewer #2 (Remarks to the Author)

I co-reviewed this manuscript with one of the reviewers who provided the listed reports. This is part of the Nature Communications initiative to facilitate training in peer review and to provide appropriate recognition for Early Career Researchers who co-review manuscripts.

Response/Action: We thank Reviewer #2 for their contribution to the review process.

Reviewer #4 (Remarks to the Author)

The additional adjusted analysis and clarification of methods meaningfully strengthen the manuscript. I appreciate the inclusion of the multivariable OS model and transparent reporting of effect sizes and confidence intervals. The interpretation as exploratory and hypothesis-generating is appropriate.

Response/ Action: We thank Reviewer #4 for constructive feedback throughout the revision process.
