# Supplementary material for: A tumor profiling resource for ovarian cancer: insights into chemotherapy-driven heterogeneity and personalized treatment strategy
Source: Nat Commun. 2026 Jul 13;17:8624. doi: 10.1038/s41467-026-74585-w (PMC13486661; doi:10.1038/s41467-026-74585-w)
Supplement: Supplementary file 3 [file 41467_2026_74585_MOESM1_ESM.pdf]

A tumor profiling resource for ovarian cancer: insights into chemotherapy-driven heterogeneity and personalized treatment strategy

SUPPLEMENTARY DATA

<u>Supplementary figures:</u> There are 15 Supplementary Figures in total.

Supplementary Fig. 1.
Metadata of the HGSOC cohort.
Supplementary Fig. 2.
Overview of the technology coverage per sample collection time point.
Supplementary Fig. 3.
Routine diagnostics for characterization of HGSOC pathology by immunohistochemistry.
Supplementary Fig. 4.
Enhanced molecular profiling increases actionable treatment modifications in patients with HGSOC.
Supplementary Fig. 5.
The HGSOC genomic heterogeneity and TCGA integration.
Supplementary Fig. 6.
Integrated analysis on whole-genome amplification subtypes reveals marginal differences on gene and protein level.
Supplementary Fig. 7.
Cell type determination and marker expression across technologies.
Supplementary Fig. 8.
Cell type composition is partly reflected in bkRNAseq-derived molecular subtype gene expression signatures.
Supplementary Fig. 9.
Single-cell RNAseq metadata and cluster definition.
Supplementary Fig. 10.
Each tumor consists of large cancer cell fates revealed by subcluster analysis of scRNA-derived.
Supplementary Fig. 11.
Ex vivo treated cancer cells are more sensitive to chemotherapy accompanied with altered gene expression.
Supplementary Fig. 12.
Cancer cell heterogeneity defined by CyTOF.
Supplementary Fig. 13.
Heterogeneity measures and their association with clinico-pathological characteristics.
Supplementary Fig. 14.
Cancer cell heterogeneity coincides with altered cell type composition.
Supplementary Fig. 15.
Integrated analysis of clinical and molecular features reveals associations of GeneSets, WGA, and structural variations with cancer cell heterogeneity.

<u>Supplementary tables:</u> There are 2 supplementary tables in total.

Supplementary table 1 Details on technology requirements.

Supplementary table 2 Summarized clinico-pathological details.

Supplementary scRNA-seq cancer cluster description

In detail, unsupervised clustering of scRNA-derived cancer cells revealed a total of 13 clusters (**Fig. 5B**, **Supplementary Fig. 10A**), each showing variations in cell number and quality metrics. Notably, clusters 8 and 9 displayed lower gene and unique molecular identifier (UMI) of RNA transcript content, while clusters 2 and 13 had intermediate levels (**Supplementary Fig. 9A-B**). We performed differential gene expression and GeneSet Variation Analysis (GSVA) in order to manually annotate the identified cancer cell subsets. comparing all 13 cancer cell clusters together with reviewing related literature and identified distinct gene expression signatures across the entire cohort (**Fig. 5B**, **Supplementary Fig. 10B-C**, and Supplementary Data 13 **Supplementary table 3**). The largest clusters (> 10,000 cells), such as cluster 3 and 9, were enriched for samples derived from solid tumor tissue and ascites, respectively (**Fig. 5B**, **Fig. 5D**, and **Supplementary Fig. 10A**). They exhibited no specific pathway activity; however, cluster 3 displayed elevated expression of protein translation-related genes (*RACK1*, *UBA52*, or *PABPC1*), while the diffuse ascites-enriched cluster 9 was elevated for *MTX1* expression. Medium-sized clusters (such as clusters 2, 4, 6, and 8, each with > 5,000 cells) exhibited distinct expression profiles, indicative of specific cellular functions. For instance, cluster 2 was characterized by RNA and protein processing-related gene expression, featuring genes such as *CCNK, HERC2, GIGYF1, RNF207, CCNL1*. This cluster also showed expression of classical HGSOC markers including *PAX8, ESR1, MUC1, MUC16, WT1, ERBB2, CDH1,* and *SOX17*. Additionally, it demonstrated increased mitochondrial (MT) gene content, and enriched autophagy. However, there was a decrease in energy and metabolic pathway activity, indicative of stress and nutrient deprivation. Cluster 4 displayed classical epithelial gene signatures (*LGALS3, MAL2, CLDN7, KRT7*), cluster 6 a proliferative-like signature (*MKI67, MELK, BRCA1, EZH2, PARP1*), and cluster 8 had an immune-like profile (*LEF1, GZMB, SH3KBP1, HLA-DRA, CXCR4*). Clusters 1, 10, 11, and 12 had less than 5,000 cells. Cluster 1, characterized as immunoreactive-like, expressed interferon-stimulated genes such as *ISG15, OAS1, IFI35, IFI44, IFIT3*, along with literature-curated genes (*IDO1, CXCL11, and CXCL10*). Cluster 10 exhibited high expression of epithelial-to-mesenchymal transition (EMT)-like secreted chemokines of the C-X-C motif family and TNF. Cluster 11 was identified as a cancer stem cell-like group displaying elevated expression of stem cell markers, including *CLU, LY6E, ALDH2,* and *CD9.* Additionally, this cluster also exhibited expression of epithelial genes such as *EPCAM, CLDN7,* and *MSLN*. Cancer cell stress-like cluster 12 was characterized by its high and confined expression of stress-related genes (*ZFAND2A, BAG3, HSPA6, HSPE1, FKBP4, HSP90AB1, TCP1, HSPA1B,* and *SERPINH1*). Cluster 13 enriched in ascites samples exhibited diffuse gene expression, while clusters 5 and 7 had a low cell count. Cluster 5 expressed the genes *SST* and *MIF* and cluster 7 exhibited a ciliated signature (*PIFO*). Further investigations using GSVA scores supported curated cancer cell cluster annotations (**Supplementary Fig. 10**). Here, the proliferative cluster 6 was enriched for *KEGG_MISMATCH_REPAIR* and *04_Cell_cycle_FT* [67], and Proliferative_DNA_repair. Immune-like cluster 8 was elevated in GeneSets indicating cytokine production and chemotaxis. Cluster 2 displayed enriched GeneSets assigned to RNA processing and autophagy while metabolic pathways were decreased (e.g. TCA_cycle or *WP_GLYCOLYSIS_AN_GLUCONEOGENESIS*). The immunoreactive cluster 1 displayed

upregulated GeneSets *JNCI_HGSOC_immuno-reactive* [64] and was primarily linked to interferon signaling (**Supplementary Fig. 10C**).

Supplementary Table 1. Due to limited cell numbers and different quality criteria from the different technology nodes, not all samples could be analyzed by all technologies. Cells were allocated based on their number and priority. When the total cell number did not meet the technology requirements, priority was given to drug testing nodes. Excess cells were stored and returned to the biobank upon project completion. In cases of low cell numbers, a priority list based on technology capabilities, reference data availability, and robustness was used, or clinical requests guide distribution. For samples with an insufficient number of cells, the cells were distributed according to a priority list that has been established based on the ability of the different technologies to support targeted therapeutic prediction.

**Tumor tissue and ascites-derived single-cell suspensions**			
Technology	Minimal cell number in million	Optimal cell number in million	Priority ranking
CyTOF	0.4	0.6	1
Pharmacoscopy	0.5	2.5	2
scOMICS	0.2	0.5	3
4i Drug Response Profiling	0.5	2.5 (1.7*)	4
Proteotyping	0.2	0.5	5
**PBMCs**			
CyTOF	0.3	0.6	1
Pharmacoscopy	1.5	8	2
Proteotyping	0.2	0.5	3
**Tumor tissue sections (FFPE)**			
Technology	Number of tissue sections		Priority ranking
FoundationOne	11		1
Digital Pathology	17		2
Proteotyping	3		3
Imaging Mass Cytometry	2		4

**in case two samples are sent out simultaneously*

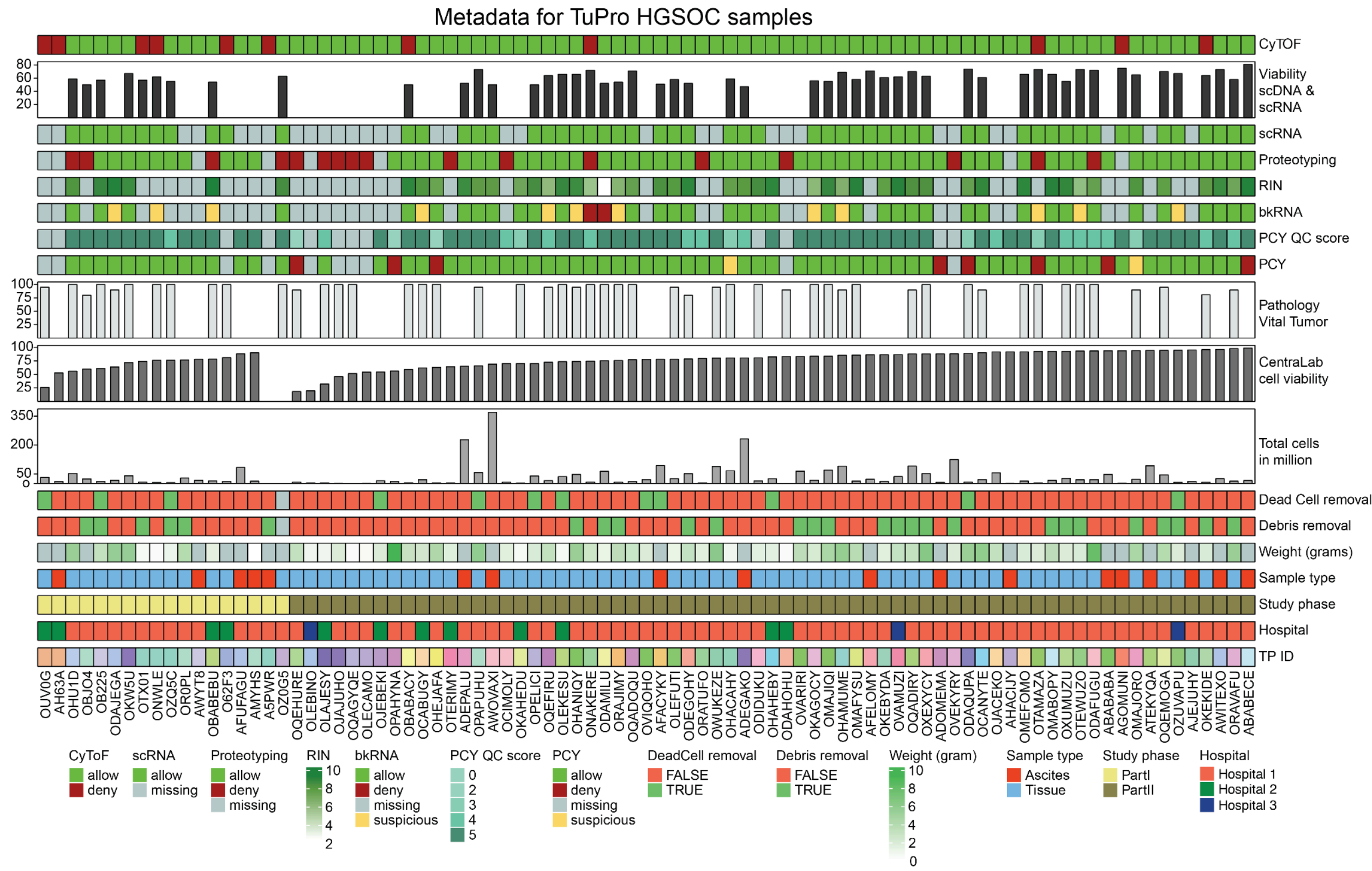

Supplementary Fig. 1. Metadata of the HGSOC cohort determined by the central lab and individual technologies. Quality control (QC) measures of HGSOC samples sorted by study phase (Part I followed by Part II) and central lab determined cell viability (gray bar chart).

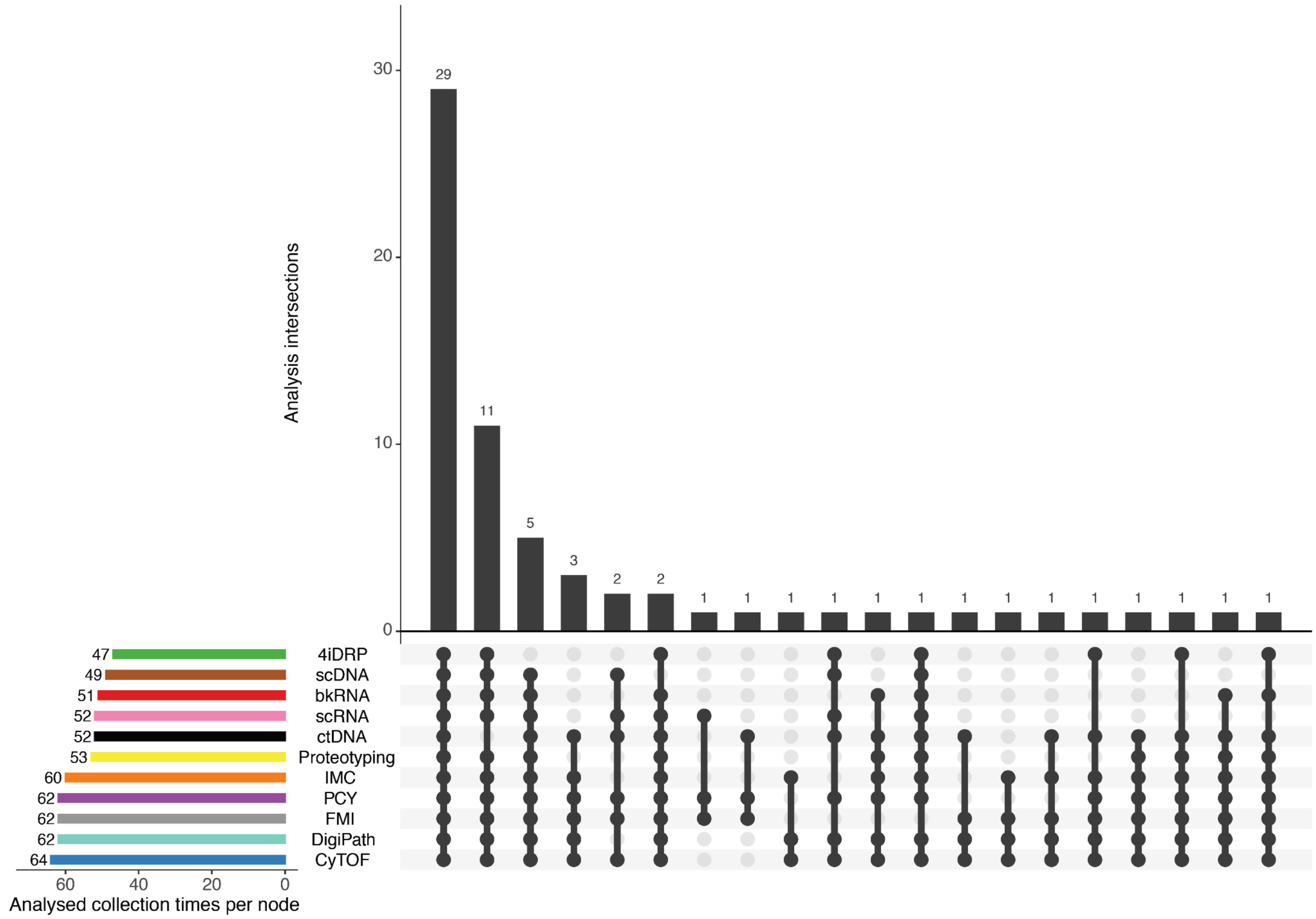

Supplementary Fig. 2. Overview of the technology coverage per sample collection time point. For each patient (n = 62) multiple samples were collected at a collection time point. Each patient had at least one collection time point, four patients had two collection time points. If a technology was applied to at least one of the patient samples collected at a specific collection time point, this technology is considered to have been applied to this time point. For each applied combination of technologies across samples collected at one specific time point (black bullets), the Upset plot shows the number of collection time points for which the respective technology combination was applied (black bars). The colored horizontal bars show the number of time points for which a specific technology was applied.

Supplementary Table 2. The table presents the summarized clinicopathological characteristics of the HGSOC patient cohort (n=62 patients), categorized by patients enrolled with adjuvant (chemo-naive) or neoadjuvant chemotherapy (NACT) and disease recurrence (relapse). *P*-values for categorical variables were derived using a chi-square test of independence.

	Chemo_ naive (N=35)	NACT (N=15)	Relapse (N=16)	Overall* (N=66)	*p*-value
**Hospital**					
Hospital 1	26 (74.3%)	11 (73.3%)	15 (93.8%)	52 (78.8%)	0.518
Hospital 2	6 (17.1%)	4 (26.7%)	1 (6.3%)	11 (16.7%)	
Hospital 3	3 (8.6%)	0 (0%)	0 (0%)	3 (4.5%)	
**TuPro_phase**					
PoC	4 (11.4%)	3 (20.0%)	2 (12.5%)	9 (13.6%)	0.847
PartII	30 (85.7%)	11 (73.3%)	14 (87.5%)	55 (83.3%)	
Missing	1 (2.9%)	1 (6.7%)	0 (0%)	2 (3.0%)	
**Number of biospecimens per patient sent to central lab**					
1	21 (60.0%)	12 (80.0%)	15 (93.8%)	48 (72.7%)	0.286
2	12 (34.3%)	2 (13.3%)	1 (6.3%)	15 (22.7%)	
3	2 (5.7%)	1 (6.7%)	0 (0%)	3 (4.5%)	
**Priority sample central node**					
omentum	26 (74.3%)	12 (80.0%)	1 (6.3%)	39 (59.1%)	7.4e-05
ovary	5 (14.3%)	3 (20.0%)	0 (0%)	8 (12.1%)	
ascites	2 (5.7%)	0 (0%)	5 (31.3%)	7 (10.6%)	
peritoneum	1 (2.9%)	0 (0%)	4 (25.0%)	5 (7.6%)	
lymph_node	1 (2.9%)	0 (0%)	4 (25.0%)	5 (7.6%)	
other	0 (0%)	0 (0%)	2 (12.5%)	2 (3.0%)	
**Age in years**					
40-49	3 (8.6%)	0 (0%)	0 (0%)	3 (4.5%)	0.971
50-59	6 (17.1%)	4 (26.7%)	3 (18.8%)	13 (19.7%)	
60-69	8 (22.9%)	3 (20.0%)	5 (31.3%)	16 (24.2%)	
70-79	11 (31.4%)	3 (20.0%)	6 (37.5%)	20 (30.3%)	
80-89	6 (17.1%)	4 (26.7%)	2 (12.5%)	12 (18.2%)	
90-99	1 (2.9%)	1 (6.7%)	0 (0%)	2 (3.0%)	
**Tumor origin (diagnosis)**					
ovary	18 (51.4%)	8 (53.3%)	9 (56.3%)	35 (53.0%)	0.973
tubal	16 (45.7%)	6 (40.0%)	7 (43.8%)	29 (43.9%)	
peritoneal	1 (2.9%)	1 (6.7%)	0 (0%)	2 (3.0%)	

**Histology**					
serous	35 (100%)	15 (100%)	16 (100%)	66 (100%)	3.48e-11
**Histological grade**					
3	35 (100%)	15 (100%)	16 (100%)	66 (100%)	3.48e-11
**FIGO stage**					
I	1 (2.9%)	0 (0%)	1 (6.3%)	2 (3.0%)	0.591
III	20 (57.1%)	7 (46.7%)	12 (75.0%)	39 (59.1%)	
IV	14 (40.0%)	8 (53.3%)	3 (18.8%)	25 (37.9%)	
**Lymph node (N) at diagnosis**					
0	8 (22.9%)	1 (6.7%)	3 (18.8%)	12 (18.2%)	0.909
1	10 (28.6%)	3 (20.0%)	5 (31.3%)	18 (27.3%)	
Missing	17 (48.6%)	11 (73.3%)	8 (50.0%)	36 (54.5%)	
**Distant metastasis (M) at diagnosis**					
0	9 (25.7%)	4 (26.7%)	5 (31.3%)	18 (27.3%)	0.825
1	12 (34.3%)	4 (26.7%)	3 (18.8%)	19 (28.8%)	
Missing	14 (40.0%)	7 (46.7%)	8 (50.0%)	29 (43.9%)	
**Residual disease at primary or interval debulking surgery**					
0 (no residual disease)	17 (48.6%)	3 (20.0%)	5 (31.3%)	25 (37.9%)	0.216
1 (Residual disease)	14 (40.0%)	10 (66.7%)	10 (62.5%)	34 (51.5%)	
Missing	4 (11.4%)	2 (13.3%)	1 (6.3%)	7 (10.6%)	
**Platinum status**					
no_chemotherapy	3 (8.6%)	0 (0%)	0 (0%)	3 (4.5%)	0.0949
not_available	11 (31.4%)	1 (6.7%)	1 (6.3%)	13 (19.7%)	
refractory	3 (8.6%)	4 (26.7%)	0 (0%)	7 (10.6%)	
resistant	0 (0%)	0 (0%)	1 (6.3%)	1 (1.5%)	
sensitive	18 (51.4%)	10 (66.7%)	14 (87.5%)	42 (63.6%)	

*clinicopathological data provided refer to diagnosis given to the patient

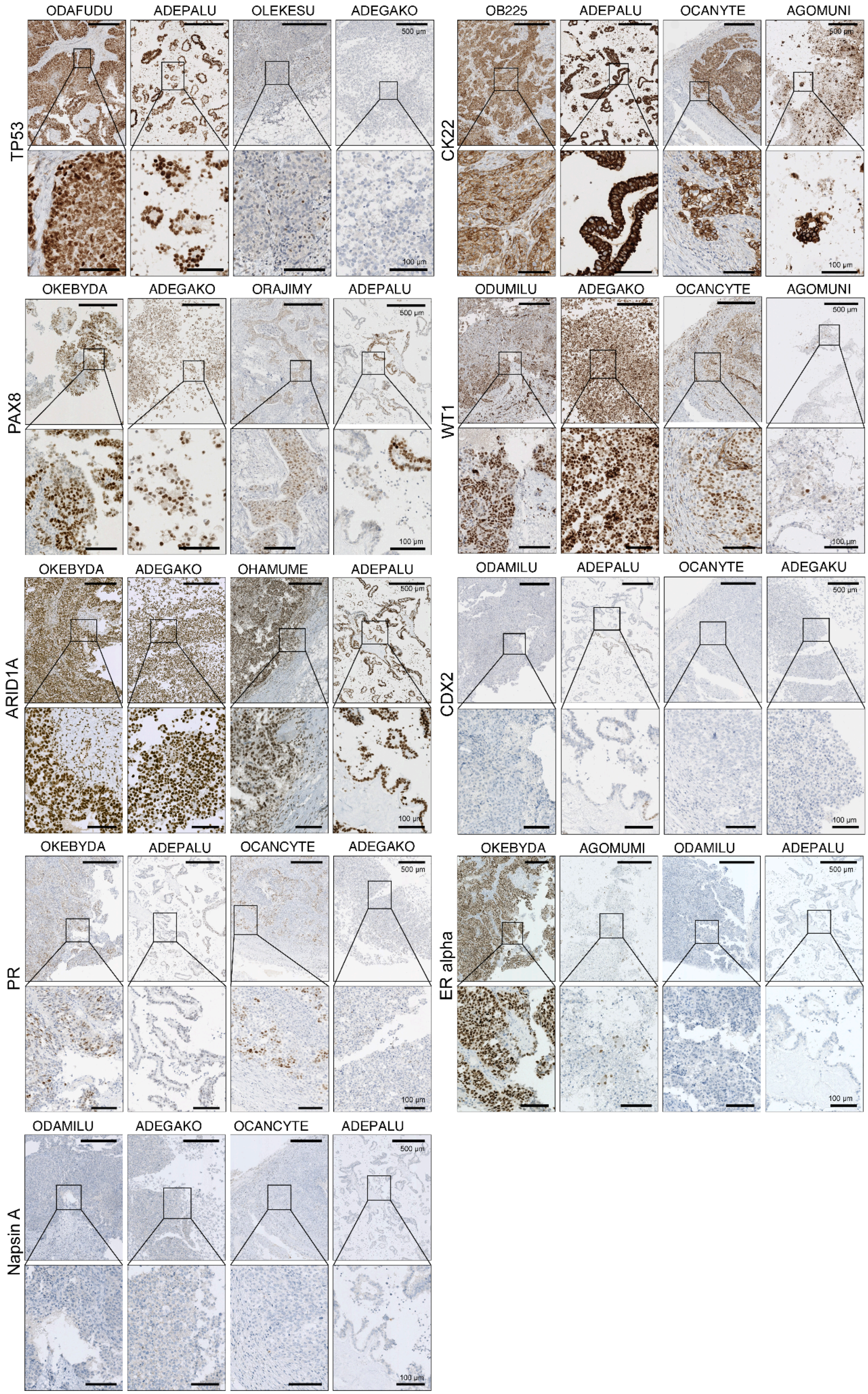

TP53
ODAFUDU
ADEPALU
OLEKESU
ADEGAKO
CK22
OB225
ADEPALU
OCANYTE
AGOMUNI
PAX8
OKEBYDA
ADEGAKO
ORAJIMY
ADEPALU
WT1
ODUMILU
ADEGAKO
OCANCYTE
AGOMUNI
ARID1A
OKEBYDA
ADEGAKO
OHAMUME
ADEPALU
CDX2
ODAMILU
ADEPALU
OCANYTE
ADEGAKU
PR
OKEBYDA
ADEPALU
OCANCYTE
ADEGAKO
ER alpha
OKEBYDA
AGOMUMI
ODAMILU
ADEPALU
Napsin A
ODAMILU
ADEGAKO
OCANCYTE
ADEPALU
500 µm
100 µm

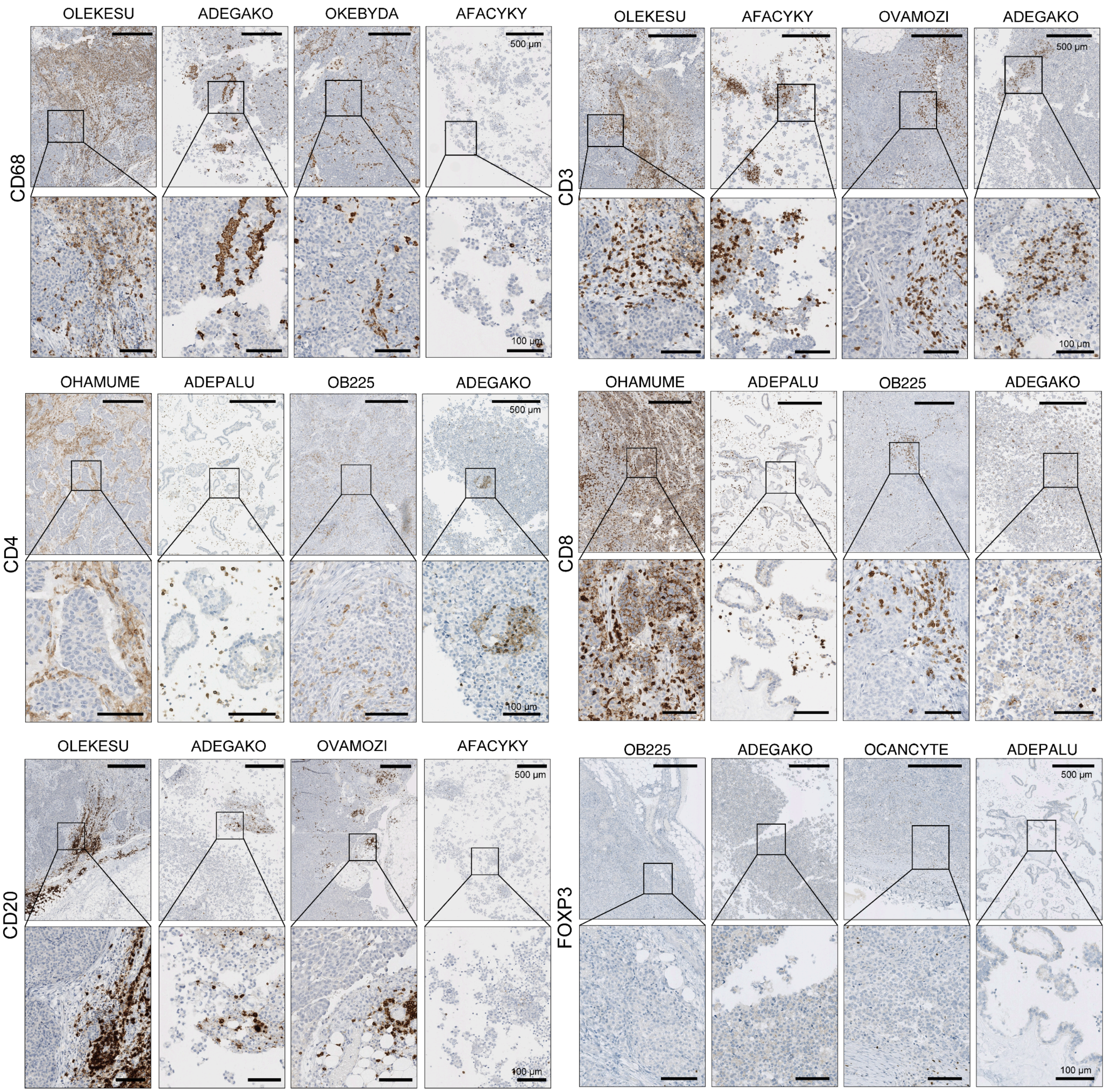

Supplementary Fig. 3. Routine diagnostics for characterization of HGSOC pathology by immunohistochemistry. Tumor (top panel) and immune (bottom panel) marker set run on each prioritized sample using immunohistochemistry and digital pathology. A total of four representative images per marker.

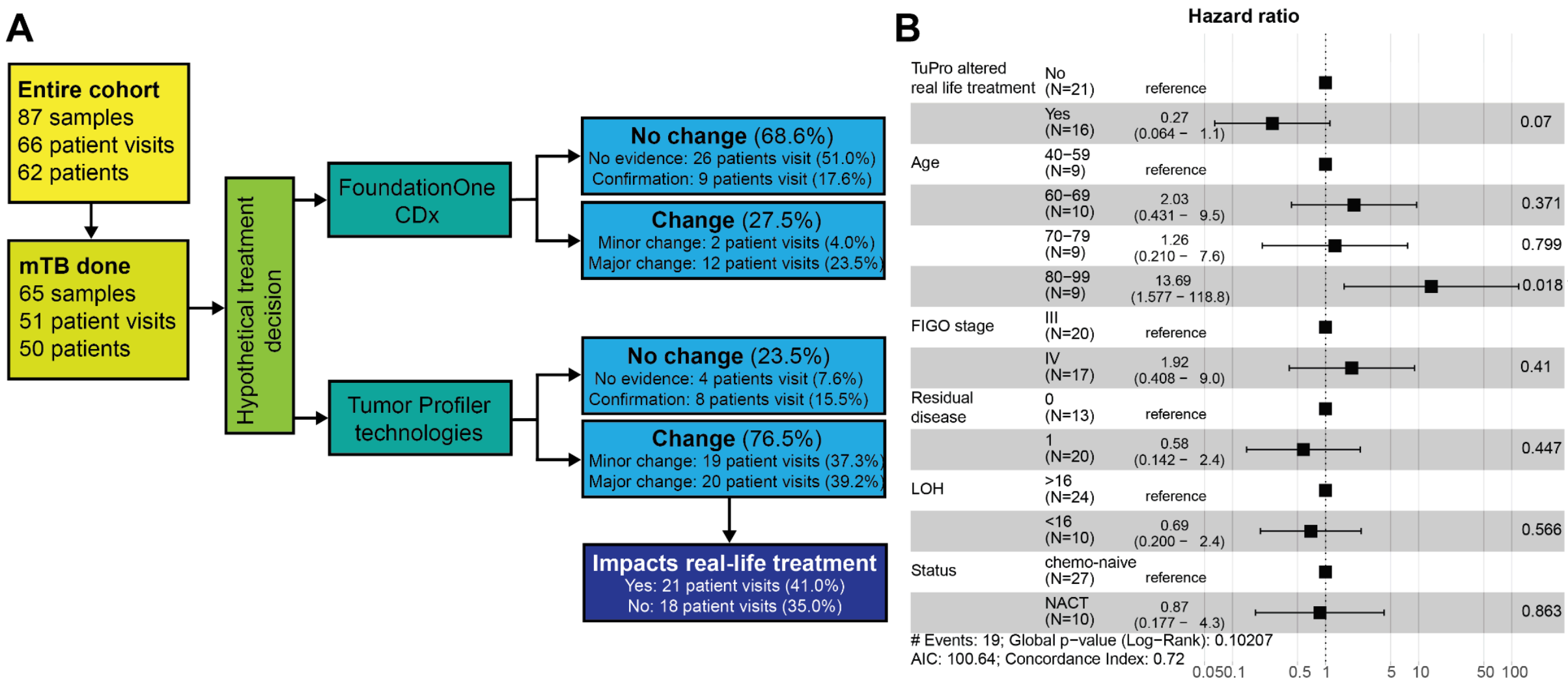

Supplementary Fig. 4. Enhanced molecular profiling increases actionable treatment modifications in patients with HGSOC. A) The figure illustrates the flow of patient samples analyzed and presented to the mTB summarizing the impact of molecular profiling on hypothetical treatment decisions. A total of 87 samples from 62 patients were included in the cohort, with 65 samples from 50 patients undergoing molecular tumor board (mTB) analysis. **B)** Multivariable Cox proportional hazards regression analysis of overall survival measured from initial diagnosis in newly diagnosed HGSOC patients. Estimated hazard ratios (HRs, squares) and corresponding 95% confidence intervals (error bars) are shown for TuPro-altered real-life treatment and clinical covariates, including age, FIGO stage, residual disease, loss of heterozygosity (LOH), and treatment status (chemo-naïve versus post-NACT). The reference category is indicated for each variable. The vertical dashed line denotes HR = 1. Model performance metrics are shown below the plot (number of events, global log-rank p-value, Akaike information criterion (AIC), and concordance index).

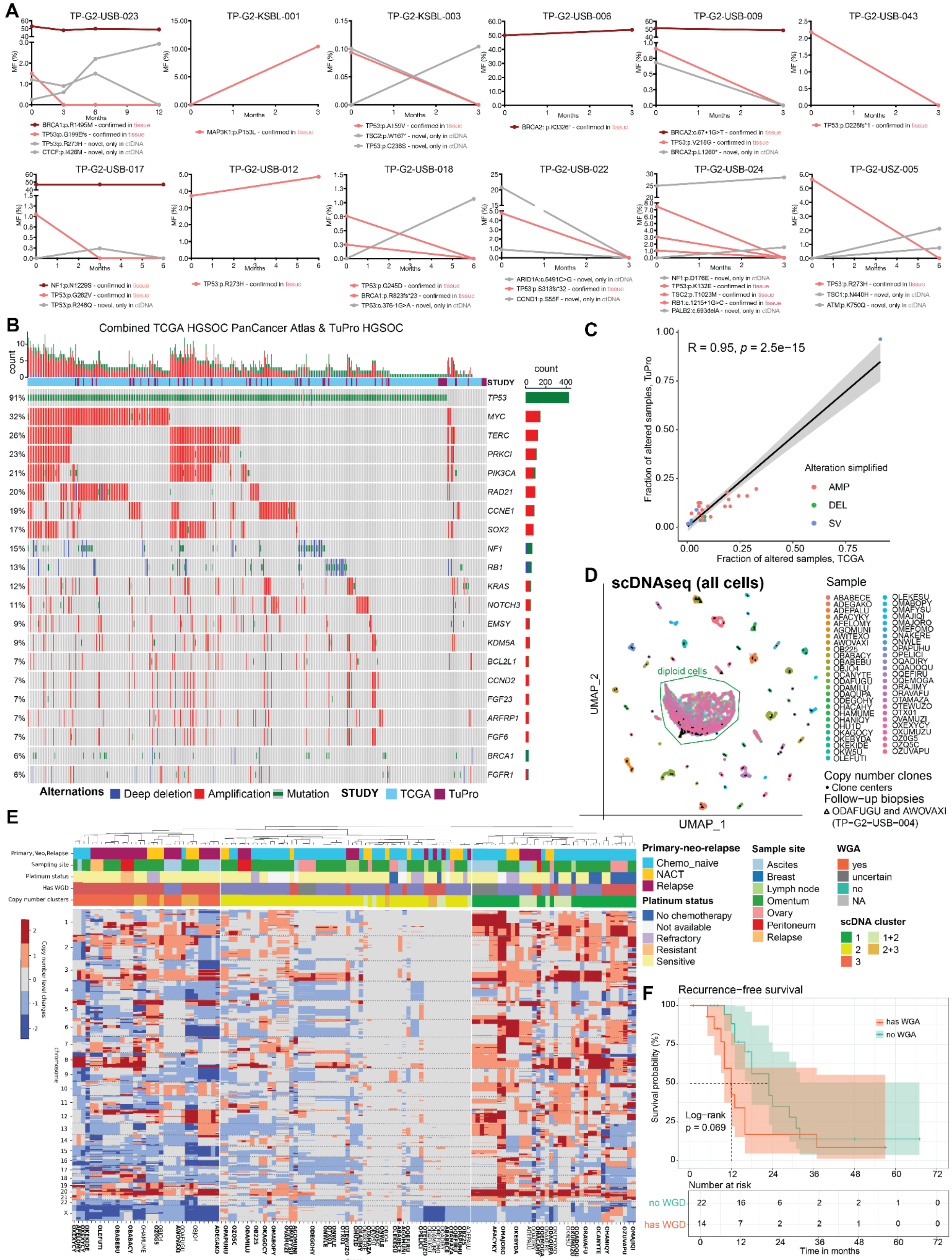

Supplementary Fig. 5. The HGSOC genomic heterogeneity and TCGA integration. A) Line charts with all detected mutations over time in ctDNA for individual patients. **B)** TuPro HGSOC integration into

Ovarian Serous Cystadenocarcinoma TCGA PanCancer mutational profile showing TuPro most frequently (≥ 5%) altered genes. **C)** Correlation plot of most frequently occurring mutational alterations in TuPro aligned with Ovarian Serous Cystadenocarcinoma TCGA PanCancer, statistical results are from Pearson's correlation test. The black line represents a linear regression fit, and the shaded area the corresponding 95% confidence interval. **D)** Normalised read counts per bin for each cell projected into 2D, along with the called copy number profiles of each clone showing strong separation by patient apart from for the diploid (non-tumour) cells in the center. **E)** The copy number profile of each clone detected (rows), samples where all the clones are in the same clade of the hierarchical dendrogram are highlighted bold, unlike those with clones in different clades. **F)** Kaplan-Meier curve for recurrence-free survival on groups stratified by scDNAseq derived WGA status (no WGA *versus* has WGA). Lines indicate the Kaplan-Meier survival probability estimate, shaded areas correspond to the 95% confidence interval and tick marks represent censored events.

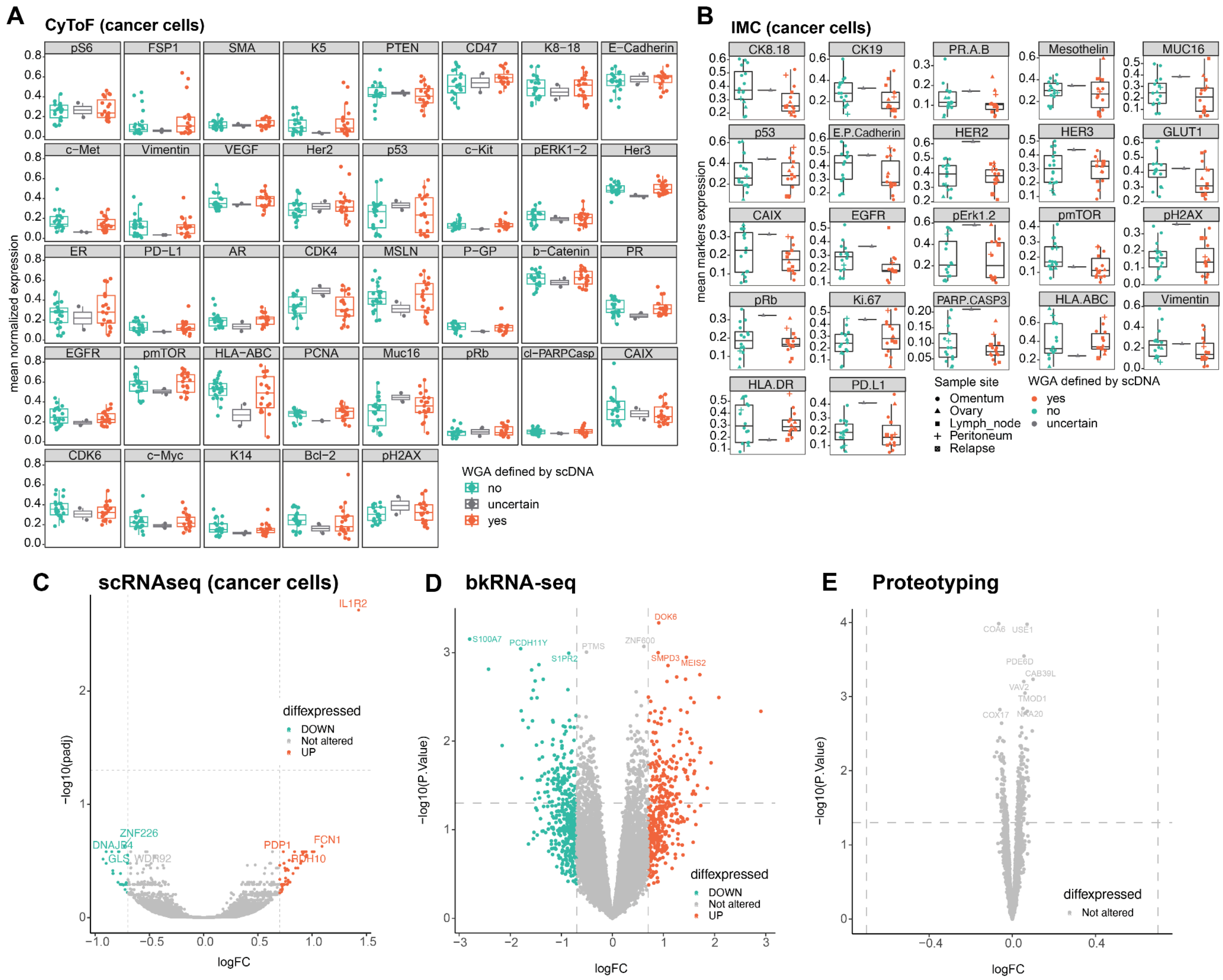

Supplementary Fig. 6. Integrated analysis on whole-genome amplification subtypes reveals marginal differences on gene and protein level. A) Mean protein expression across all HGSOC cells per sample, measured by CyTOF, for samples with and without WGA (n=46 samples). Boxes represent the median (horizontal line), interquartile range (box), and whiskers extending to 1.5x the interquartile range. Individual samples are shown as dots. **B)** Boxplots as in A) showing single antibody staining in FFPE sections using IMC (n=31 samples). **C)** Volcano plot depicting differentially expressed genes (DESeq2, see Methods) in the entire HGSOC TuPro cohort, comparing samples with whole genome amplification (WGA) to those without WGA, all defined by scDNAseq, “uncertain” samples were not included. **D)** Volcano plot bkRNA-seq and **E)** Volcano plot proteotyping.

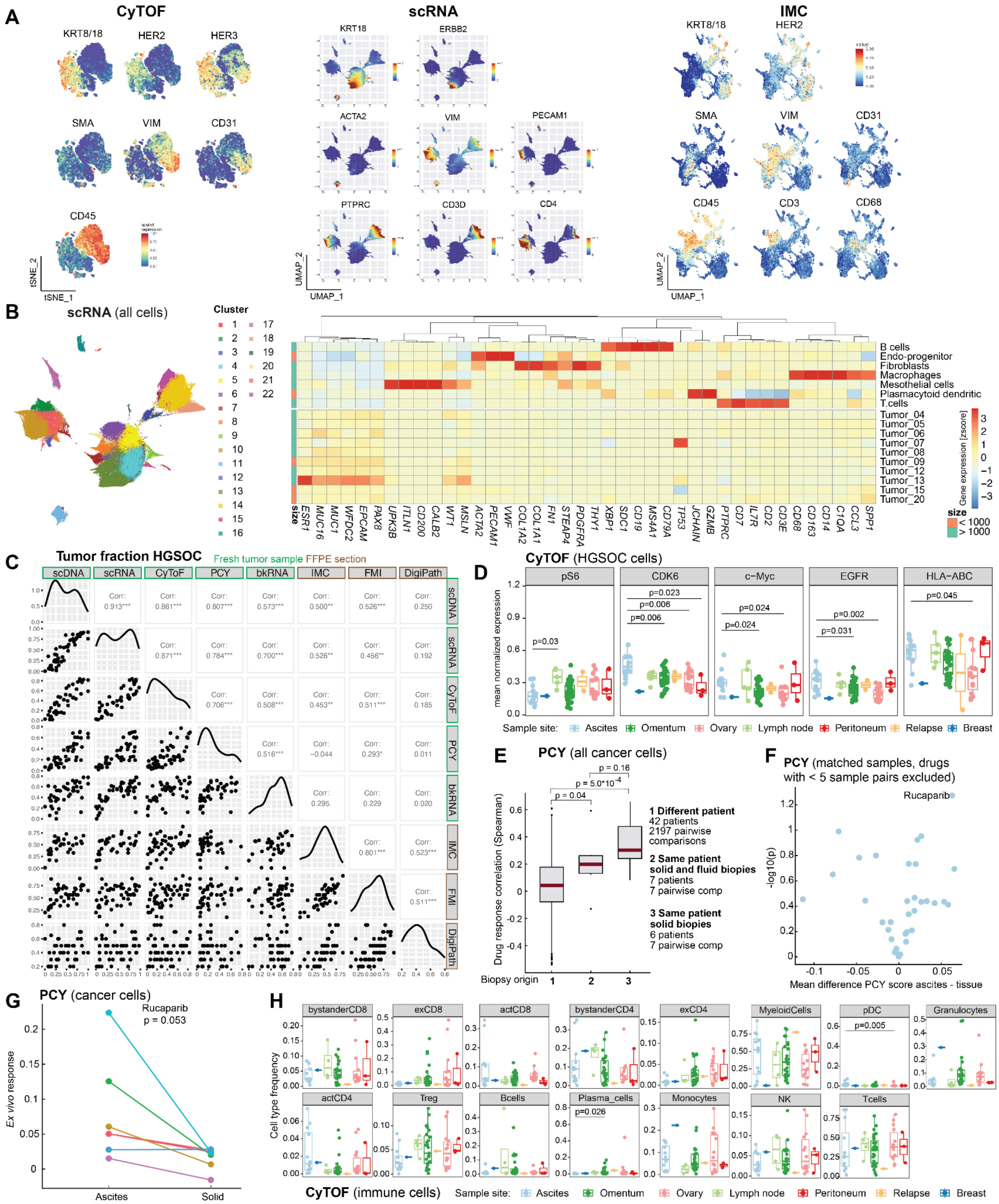

Supplementary Fig. 7. Cell type determination and marker expression across technologies. A) Visualization using *t*-SNE and UMAPs depicting the expression of selected markers for HGSOC (keratins 8 and 18 (K8/18), HER2, and HER3), fibroblasts (SMA, VIM), endothelial cells (CD31), and immune cells (CD45) across the entire cohort for data obtained by CyTOF, scRNA-seq, and IMC (from left to right). **B)** Major cell cell type classification on scRNA-seq data selected cell type marker expression. **Left:**

Cohort-wide UMAP representation of all cell types based on highly variable genes, colored by Louvain clustering. **Right:** heatmap of the mean gene expression per cell type or cluster, averaged over all cells of all samples. Average gene expression per cluster was calculated only across the most abundant cell type per cluster. **C)** Multi-technology visualization illustrates correlation for tumor fraction across 87 samples between the nodes that utilize fresh tumor samples or FFPE sections (Pearson correlation coefficient, ***$p < 0.001$, **$p < 0.01$, and *$p < 0.05$), technology [number of cells analyzed]: scRNA-seq [161,102], scDNA-seq [10,457], CyTOF [3,585,307], and PCY [91 million]**. D)** CyTOF analysis with boxplots illustrating sampling site-specific marker expression in HGSOC cells, *p*-values were calculated with a Dunn test, using BH method to correct for multiple testing across sampling sites (n=35 omentum, n=15 ascites, n=15 ovary, n=5 lymph node, n=3 peritoneum. n=2 other, n=1 breast; n=76 samples in total). **E)** Comparison of PCY-based drug responses between 1) Biopsies originating from the same patient; 2) Two solid tissue biopsies originating from the same patient and sampling time point; 3) a solid tissue biopsy and ascites originating from the same patient and sampling time point. Values represent Spearman correlation coefficient, *p*-values from two-sided two-sample Wilcoxon test. **F**) Volcano plot comparing solid tissue vs ascites only for matched sample pairs (paired *t*-test, no adjustment for multiple testing). **G**) Scatter plot illustrating a trend towards higher *ex vivo* response to rucaparib evaluated by PCY when comparing ascites and patient-matched solid tissue. Dots correspond to individual samples, colors and lines indicate matched pairs. **H)** CyTOF analysis with boxplots showing differences in immune cell type composition including plasmacytoid dendritic cells (pDC), exhausted CD8 T cells, regulatory T cells (T reg) and plasma cells, when comparing ascites with other solid tissue sampling sites. *P*-values were calculated with a Dunn test, using BH method to correct for multiple testing across sampling sites. Sampling sites included omentum (n = 27), ovary (n = 16), ascites (n = 12), lymph node (n = 4), peritoneum (n = 3), and other sites including breast (n = 2). All boxplots are formatted as described in Supplementary Fig. 6A.

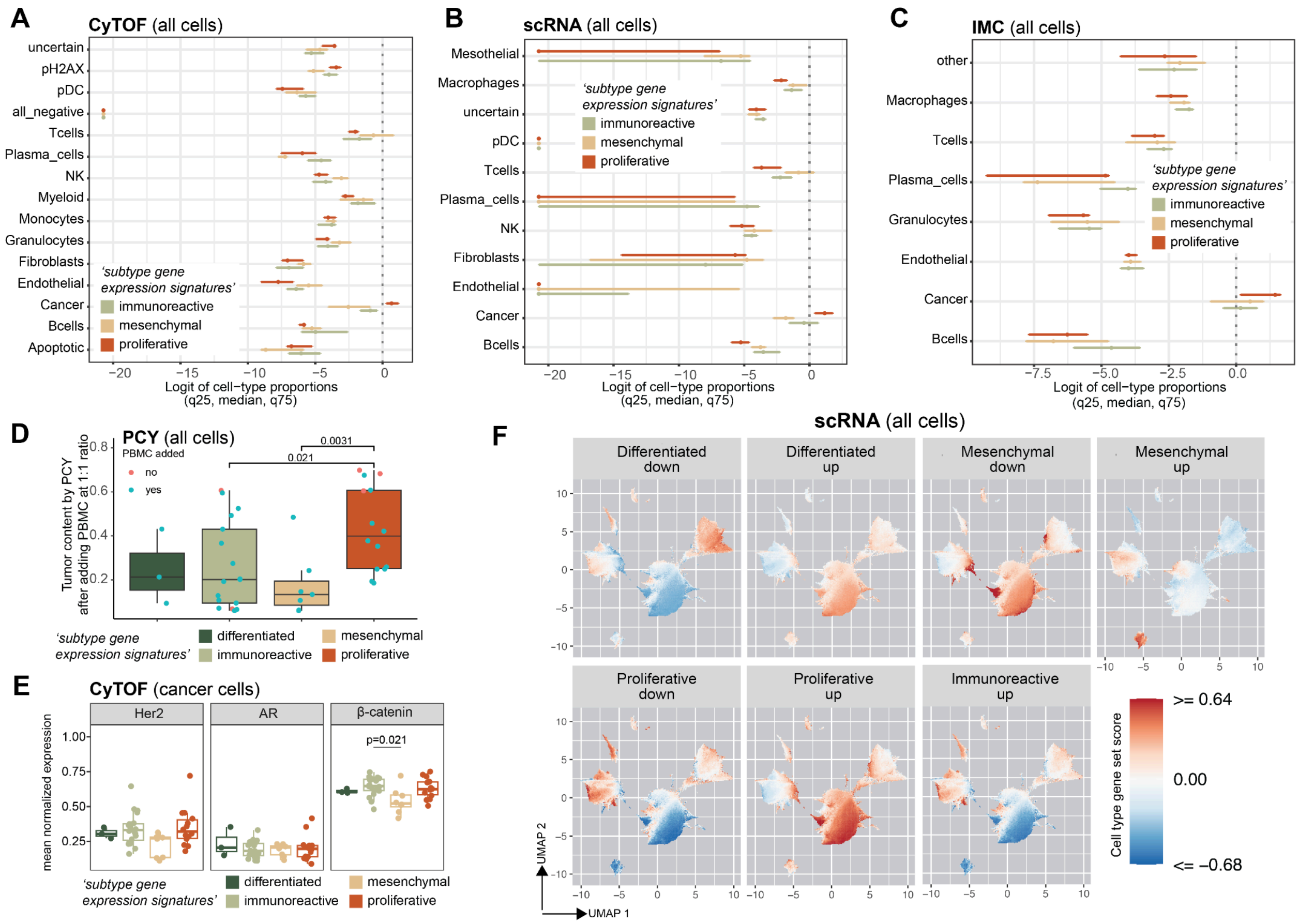

Supplementary Fig. 8. Cell type composition is partly reflected in bkRNAseq-derived molecular subtype gene expression signatures. A-C) Cell type composition of HGSOC cohort, stratified by molecular subgroups. The X-axis represents the logit cell-type proportions. The lines depict interquartile range, with dots corresponding to the median of different cell types (Y-axis) logit of proportions. Colors represent different molecular subtypes, indicating an increased proportion of cancer cells in the proliferative subtype from CyTOF, scRNAseq, and IMC data. **D**) Boxplots illustrate an increase in tumor content determined by PCY aligned with the proliferative molecular subtype. *P*-values from pairwise Wilcoxon tests, only $p < 0.05$ are shown. Molecular subtypes included immunoreactive (n = 17), proliferative (n = 15), mesenchymal (n = 7), and differentiated (n = 4). **E**). Boxplot showing the expression of selected markers on cancer cells as assessed by CyTOF. *P*-values were calculated using a Dunn test and the BH method to correct for multiple testing within each marker. Molecular subtypes included immunoreactive (n = 19), proliferative (n = 15), mesenchymal (n = 8), and differentiated (n = 3). **F**) UMAP visualization of the molecular subtypes among all cell types. Color scale represents the respective cell type gene set score. All boxplots as in supplementary Fig. 6A.

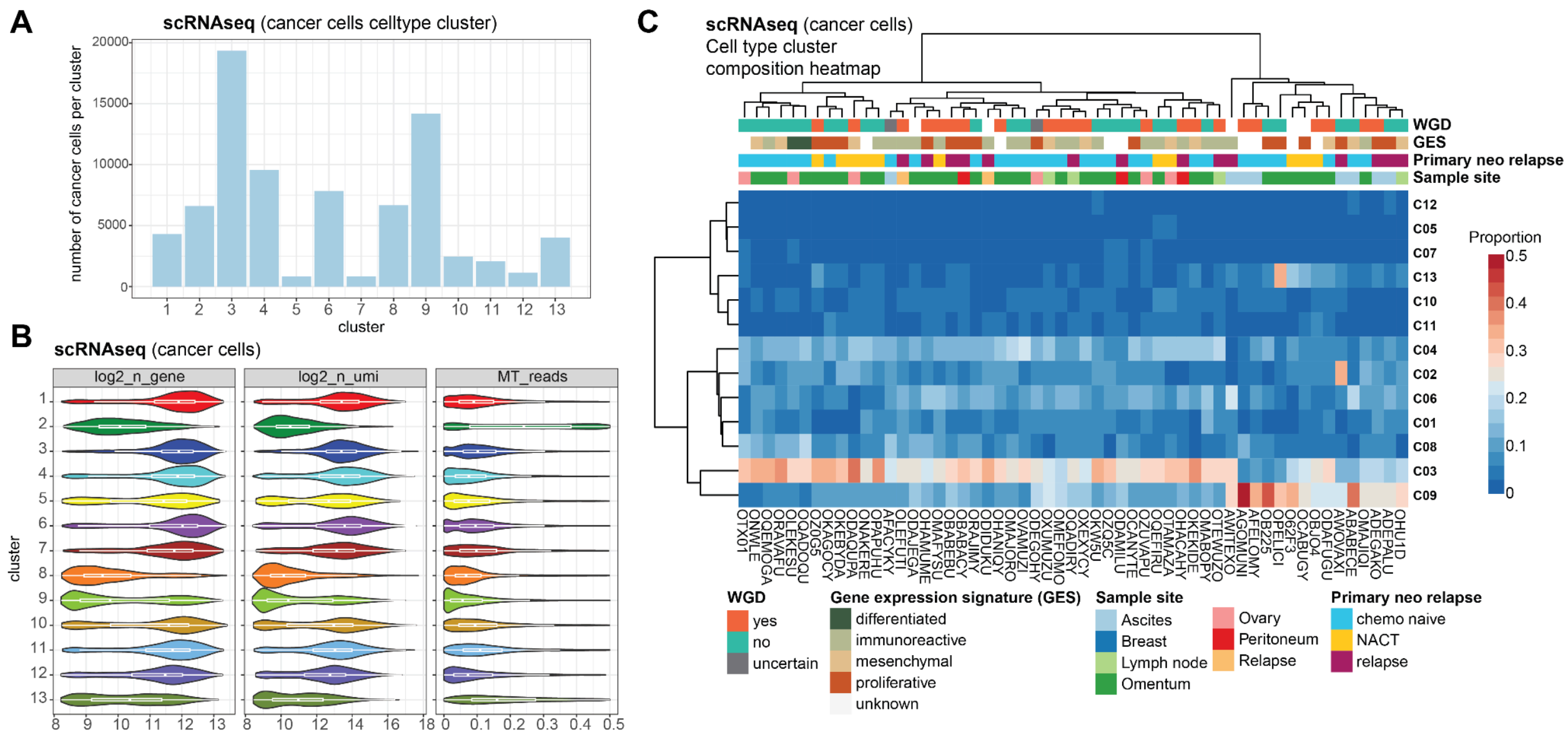

Supplementary Fig. 9. Single-cell RNAseq metadata and cluster definition. A) Barplot with the number of cancer cells for the 13 clusters. **B)** Violin plots with QC metrics showing the number of genes, the number of unique molecular identifiers (UMI), and MT content for the 13 clusters. Colors of the violins correspond to cluster IDs. Violins represent data density estimate, boxplot overlay as in Supplementary Figure 6A. **C)** Heatmap representing the composition of the 13 clusters for the patients. Patient ID is indicated at the bottom and cluster ID is on the right side. Additionally, clinical information for the patient is annotated at the top.

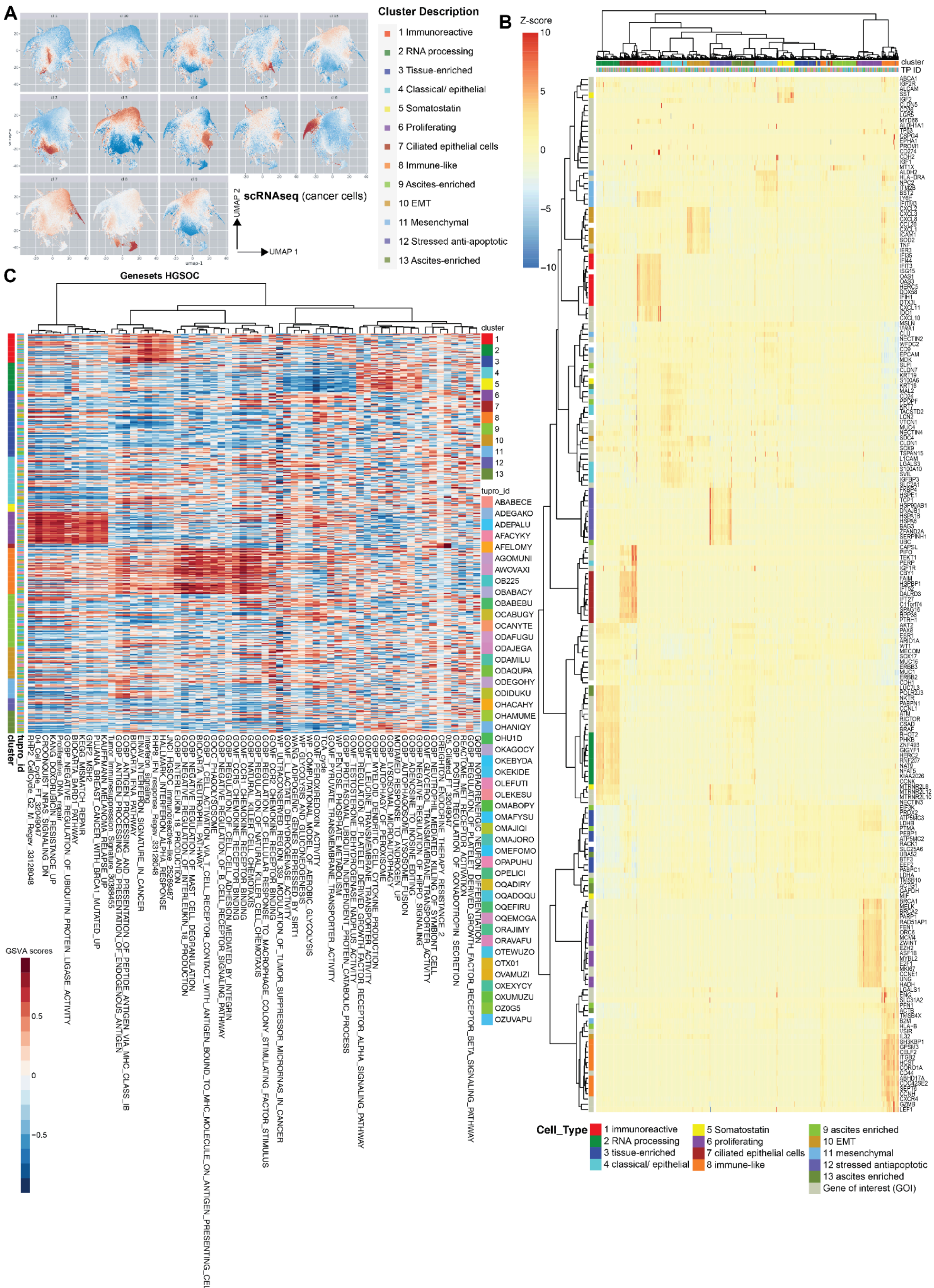
A
Cluster Description
1 Immunoreactive
2 RNA processing
3 Tissue-enriched
4 Classical/ epithelial
5 Somatostatin
6 Proliferating
7 Ciliated epithelial cells
8 Immune-like
9 Ascites-enriched
10 EMT
11 Mesenchymal
12 Stressed anti-apoptotic
13 Ascites-enriched
scRNAseq (cancer cells)
UMAP 2
UMAP 1
B
Z-score
10
5
0
−5
−10
cluster
TP ID
Cell_Type
1 immunoreactive
2 RNA processing
3 tissue-enriched
4 classical/ epithelial
5 Somatostatin
6 proliferating
7 ciliated epithelial cells
8 immune-like
9 ascites enriched
10 EMT
11 mesenchymal
12 stressed antiapoptotic
13 ascites enriched
Gene of interest (GOI)
C
Genesets HGSOC
cluster
1
2
3
4
5
6
7
8
9
10
11
12
13
tupro_id
ABABECE
ADEGAKO
ADEPALU
AFACYKY
AFELOMY
AGOMUNI
AWOVAXI
OB225
OBABACY
OBABEBU
OCABUGY
OCANYTE
ODAFUGU
ODAJEGA
ODAMILU
ODAQUPA
ODEGOHY
ODIDUKU
OHACAHY
OHAMUME
OHANIQY
OHU1D
OKAGOCY
OKEBYDA
OKEKIDE
OLEFUTI
OLEKESU
OMABOPY
OMAFYSU
OMAJIQI
OMAJORO
OMEFOMO
OPAPUHU
OPELICI
OQADIRY
OQADOQU
OQEFIRU
OQEMOGA
ORAJIMY
ORAVAFU
OTEWUZO
OTX01
OVAMUZI
OXEXYCY
OXUMUZU
OZ0G5
OZUVAPU
tupro_id
cluster
GSVA scores
0.5
0
−0.5

Supplementary Fig. 10. Each tumor consists of large cancer cell fates revealed by subcluster analysis of scRNA-derived. A) UMAP visualization and distribution of identified and annotated thirteen cancer cell clusters. Legend and description of cluster IDs refer to Fig. 5C. **B)** Gene expression heatmap and unsupervised clustering of the top ten differentially expressed genes of the 13 defined clusters over all cancer cells as well as a selection of genes of interest and extended tumor markers. **C)** Heatmap representation of Gene Set Variation Analysis (GSVA) scores from a selected list of top pathways. Only pathways that had a |logFC|>0.5 and adjusted *p*-value < 0.05 were used for the heatmap. The patient ID (TP ID) and the cluster ID (cluster) are annotated at the bottom. Differential expression analysis and GeneSet comparisons between groups of samples were performed on pseudobulk data (2). To this end, in each sample, raw counts were summed up per gene over all cells of a given type. The characteristics of the resulting data are similar to bulk sequencing count matrices and were analyzed according to the standard DESeq2 workflow (3).

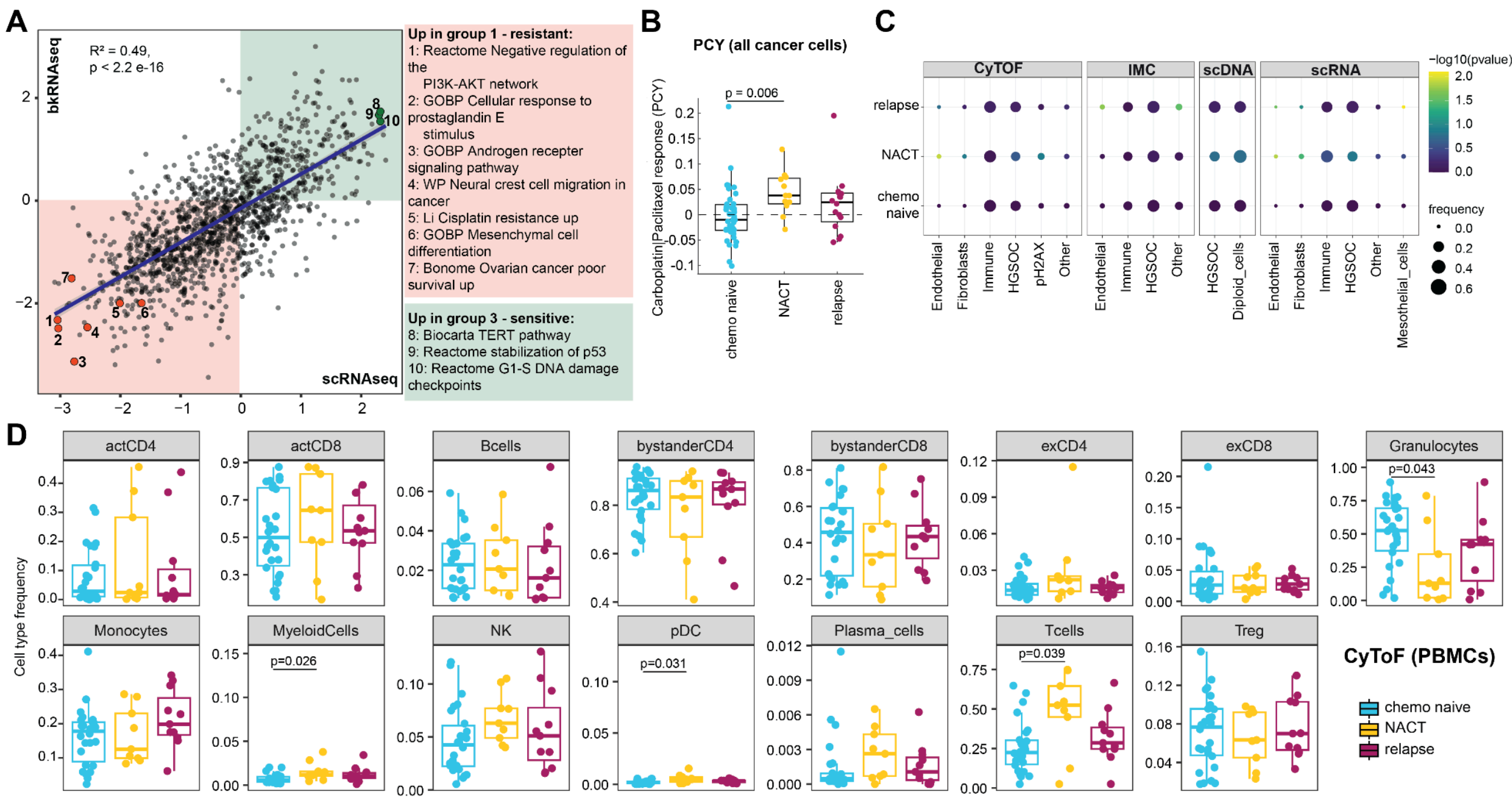

Supplementary Fig. 11. ***Ex vivo*** **treated cancer cells are more sensitive to chemotherapy accompanied with altered gene expression. A)** Differential pathway activation analysis comparing PCY response group 1 (resistant) to PCY response group 3 (sensitive). Values represent the *t*-statistic of a linear regression model applied to pathway scores derived from bulkified scRNA-seq gene expression in HGSOC cells (x-axis) and bulk RNA-seq (y-axis). Selected pathways are annotated. **B)** PCY-based *ex vivo* response to carboplatin + paclitaxel combination treatment, stratified by treatment status. *P*-values from Kruskal-Wallis anova followed by Tukey's HSD, $p > 0.05$ are not shown. Treatment status included chemo naive (n = 39), relapse (n = 16), and NACT (n = 12). **C)** Sample composition across different TuPro technologies, stratified by treatment status. Color indicates -log10 *p*-value comparing NACT to chemo naive, size of the dot indicates cell type frequency. **D)** Frequency of the indicated immune cell types identified in PBMC upon CyTOF analysis. *P*-values were calculated with a Dunn test using the BH method to correct for multiple testing across treatment types. PBMC CyTOF data were available for 46 samples, including 26 chemo-naive, 9 neo-adjuvant, and 11 relapse samples.

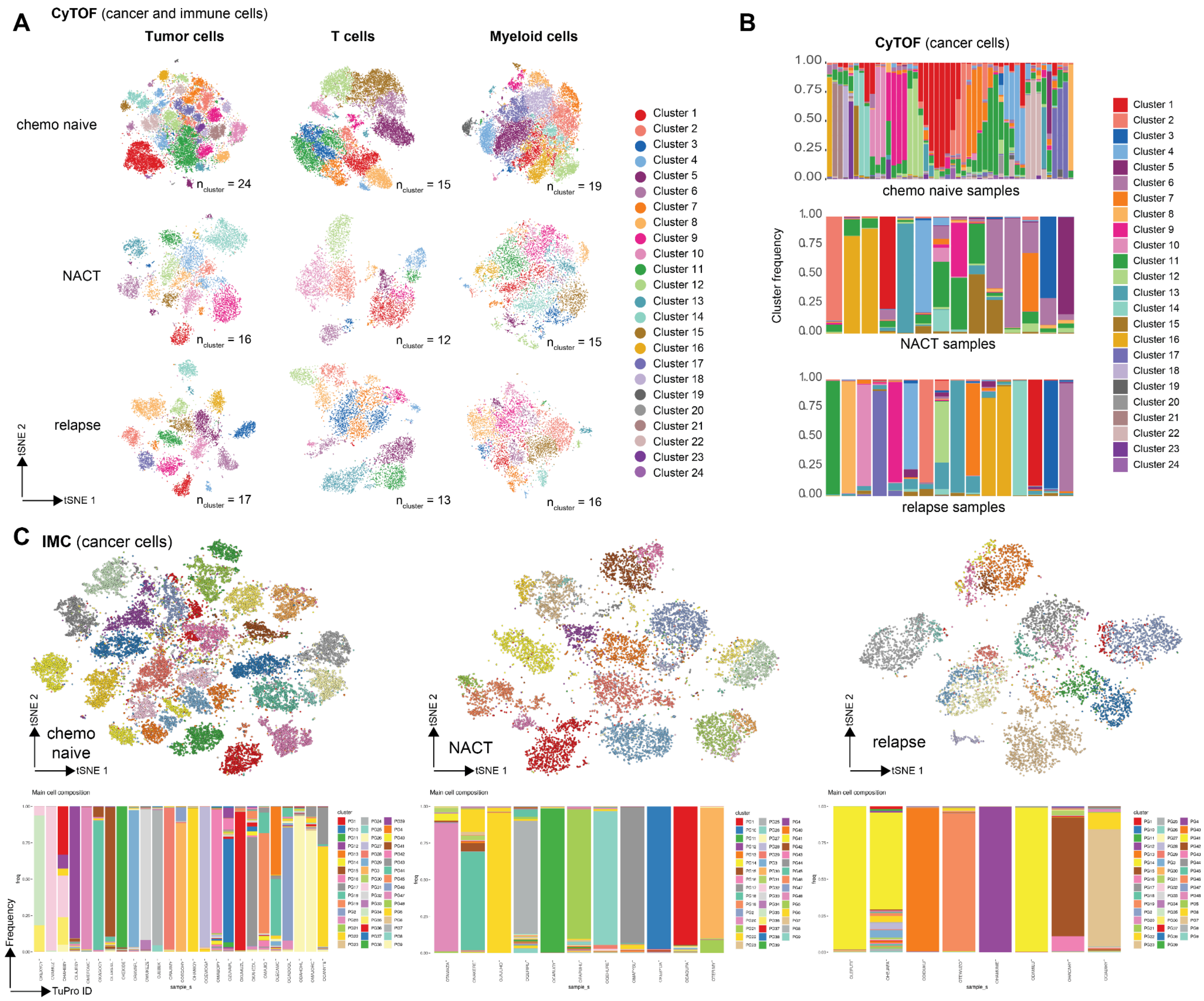

Supplementary Fig. 12. Cancer cell heterogeneity defined by CyTOF. A) t-SNE maps a maximum of 600 tumor cells, T cells, and myeloid cells per sample, with cell types in columns and split by treatment type in rows, as assessed by CyTOF. Each t-SNE map is colored by PhenoGraph clusters performed individually for each cell type and treatment. The number of clusters identified in each case is indicated below each t-SNE map. **B)** Stacked histograms showing the frequency of PhenoGraph clusters across samples for the three treatment types, generated as described in A. **C)** t-SNE maps of a maximum of 600 tumor cells per sample split by treatment type, as assessed by IMC. Each t-SNE map is colored by PhenoGraph clusters performed individually for each treatment. The frequency of the PhenoGraph clusters for each treatment type is displayed on the bottom row.

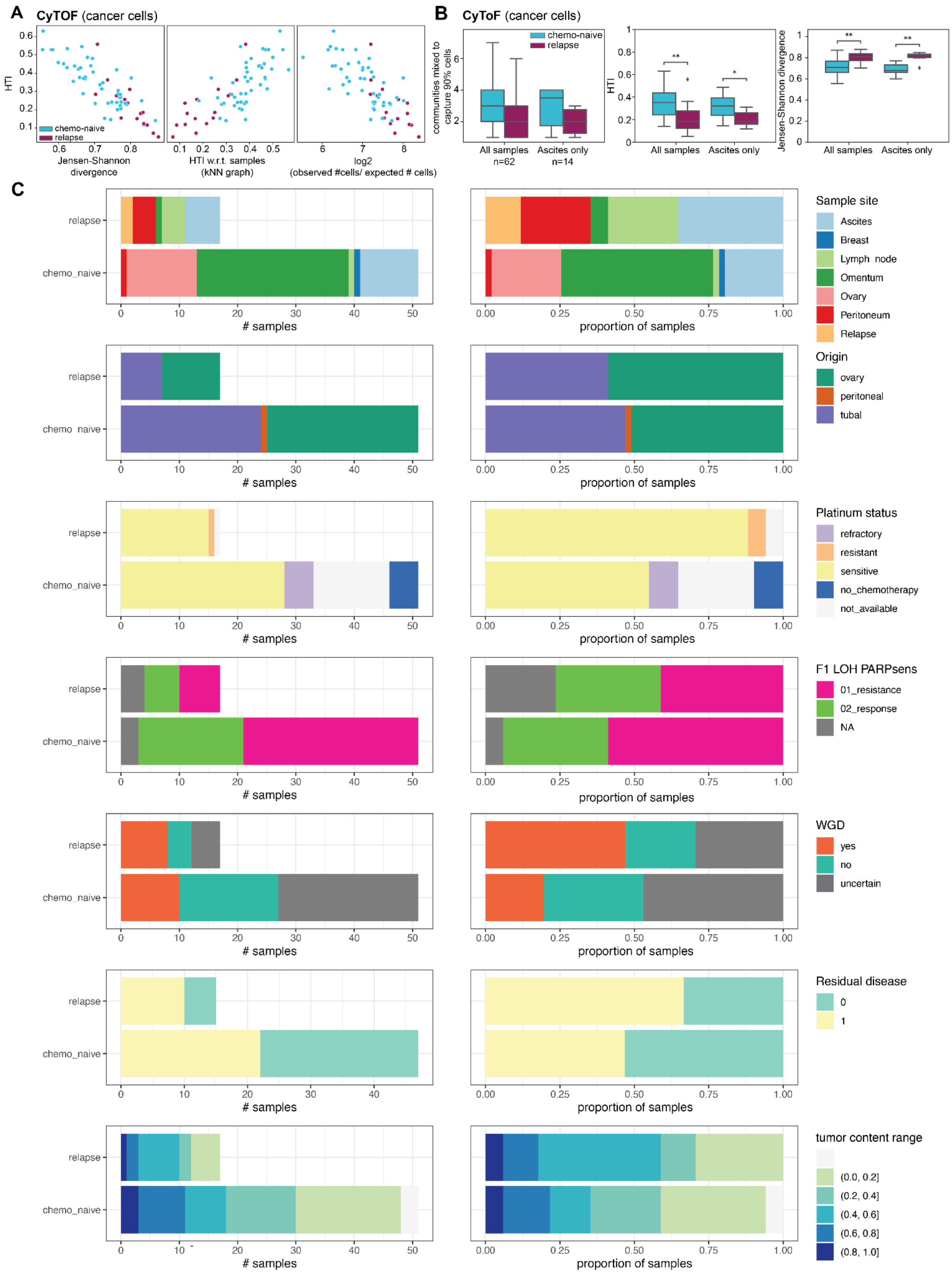
A
CyTOF (cancer cells)
HTI
chemo-naive
relapse
Jensen-Shannon divergence
HTI w.r.t. samples (kNN graph)
log2 (observed #cells/ expected # cells)
B
CyToF (cancer cells)
communities mixed to capture 90% cells
chemo-naive
relapse
All samples n=62
Ascites only n=14
HTI
All samples
Ascites only
Jensen-Shannon divergence
All samples
Ascites only
C
relapse
chemo_naive
samples
proportion of samples
Sample site
Ascites
Breast
Lymph node
Omentum
Ovary
Peritoneum
Relapse
Origin
ovary
peritoneal
tubal
Platinum status
refractory
resistant
sensitive
no_chemotherapy
not_available
F1 LOH PARPsens
01_resistance
02_response
NA
WGD
yes
no
uncertain
Residual disease
0
1
tumor content range
(0.0, 0.2]
(0.2, 0.4]
(0.4, 0.6]
(0.6, 0.8]
(0.8, 1.0]

Supplementary Fig. 13. Heterogeneity measures and their association with clinicopathological characteristics. A) Comparison of intra-tumor/heterogeneity index (y-axis) and inter-sample (x-axes) heterogeneity based on CyTOF measurements in tumor cells only. Left plot shows comparison using Jensen-Shannon divergence, while the inter-sample heterogeneity in the middle and right plots are based on a cellular kNN graph. **B)** Comparison of intra-tumor (left, middle) and inter-patient (right) heterogeneity between chemo-naive and relapse groups (color-coded), based on phenograph clustering of tumor cells profiled with CyTOF. **C)** Clinical characteristics of HGSOC cohort, stratified by chemo-status (y-axis). Each row corresponds to a different clinical feature, such as a sampling site. Left column represents the number of samples in each cluster-clinical variable combination, while the plots on the right display relative value, i.e., composition of each cluster with respect to a given clinical characteristic. All boxplots as in Supplementary Fig. 6A.

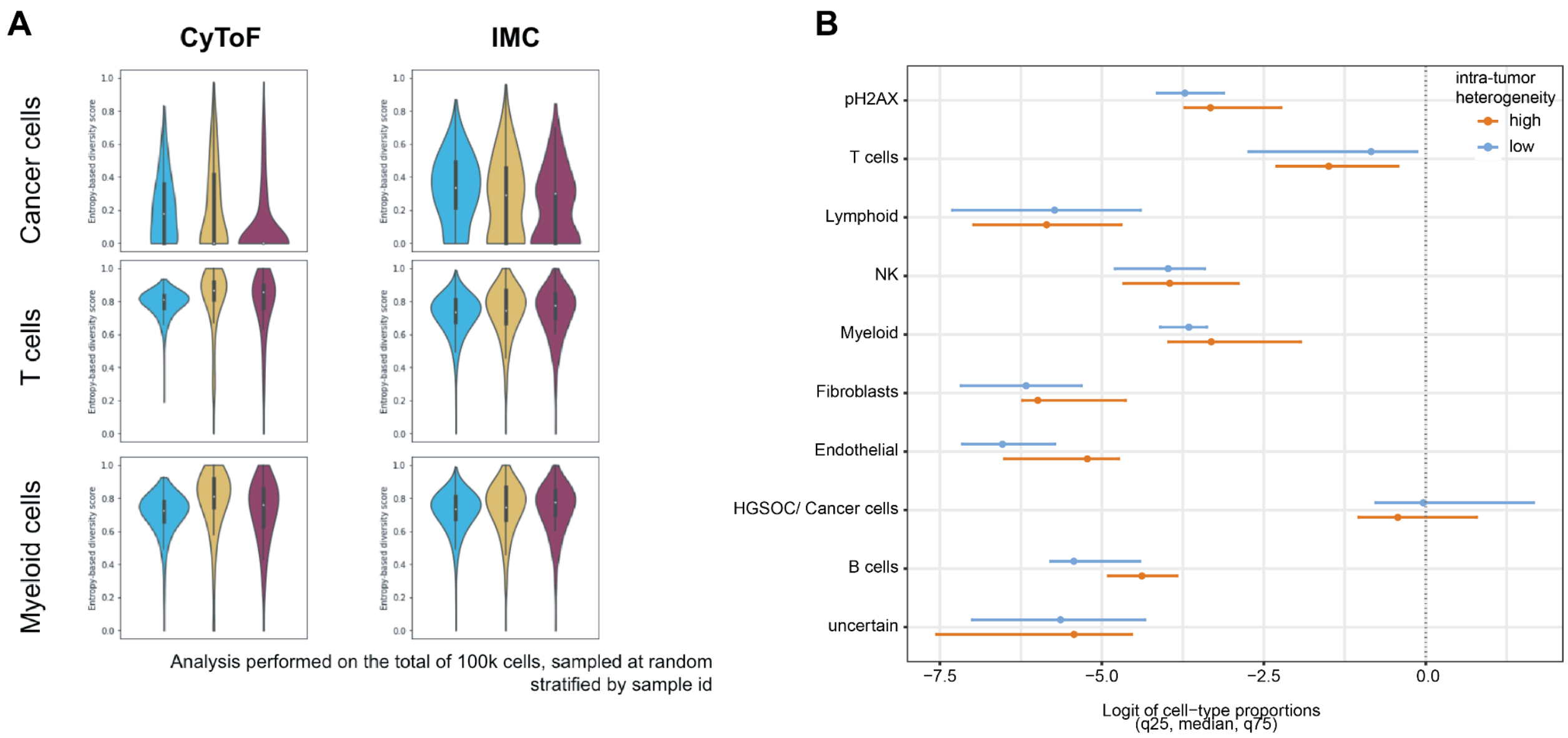

Supplementary Fig. 14. Cancer cell heterogeneity coincides with altered cell type composition. A) HTI scores in CyTOF and IMC data broken down by cell-type and sample groups (e.g. chemo-naive, NACT, relapse). **B)** Cell-type composition of chemo-naive HGSOC subcohort, stratified by median intra-tumor heterogeneity. X-axis represents the logit cell-type proportions. The lines represent IQR with dots corresponding to the median of different cell type (y-axis) logit of proportions, with colors representing high (orange) and low (blue) intra-tumor heterogeneity groups.

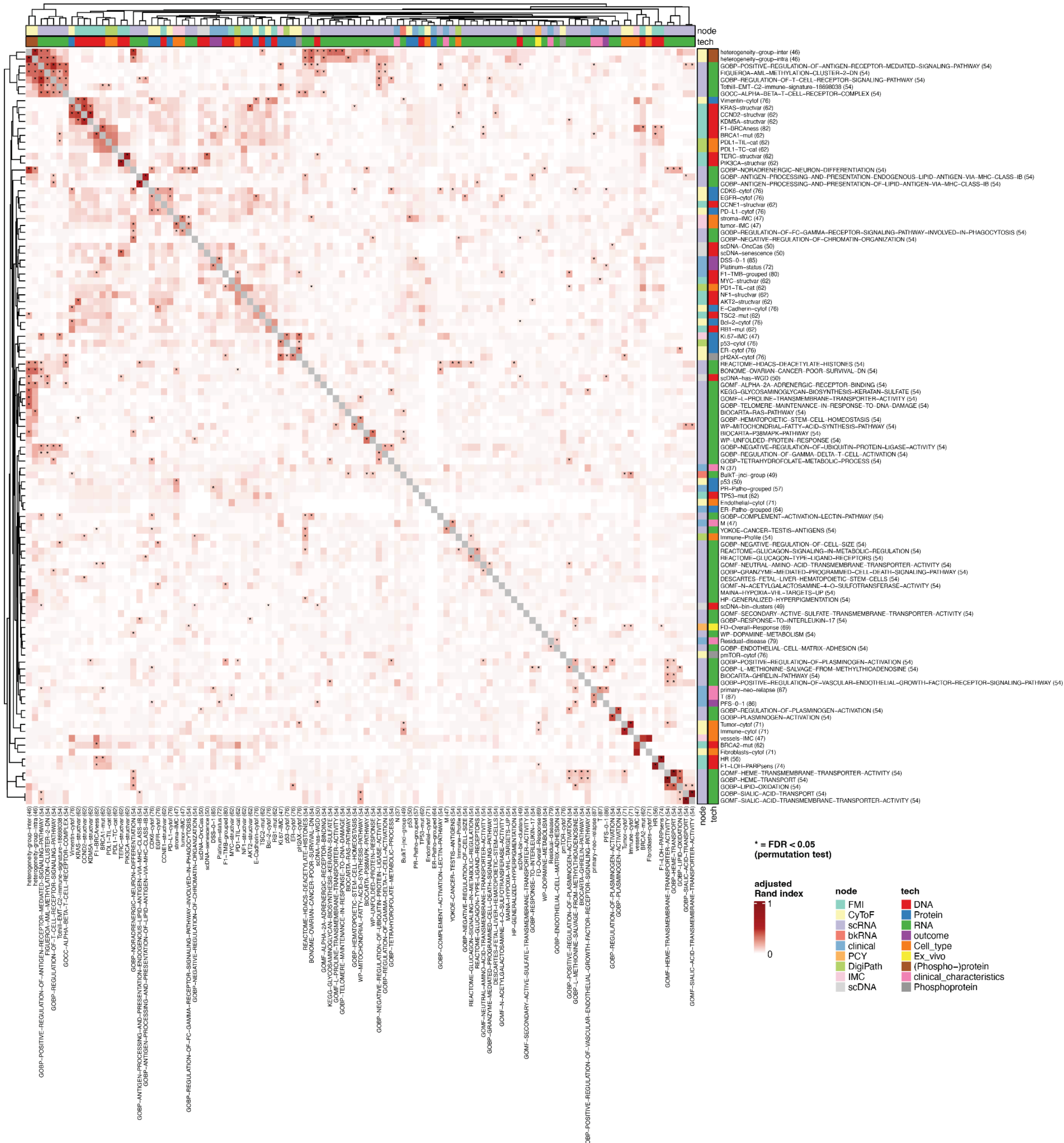

Supplementary Fig. 15. Integrated analysis of clinical and molecular features reveals associations of GeneSets, WGA, and structural variations with cancer cell heterogeneity. Heatmap representing similarity of patient groupings derived by different nodes and technologies using adjusted Rand index, with row legends indicating the respective node and technology for each grouping. Comparisons for which the adjusted Rand Index is statistically significant using permutation testing (FDR < 0.05) are marked (*).
