# Supplementary material for: A tumor profiling resource for ovarian cancer: insights into chemotherapy-driven heterogeneity and personalized treatment strategy
Source: Nat Commun. 2026 Jul 13;17:8624. doi: 10.1038/s41467-026-74585-w (PMC13486661; doi:10.1038/s41467-026-74585-w)
Supplement: Supplementary file 4 [file 41467_2026_74585_MOESM2_ESM.pdf]

Descriptions of Additional Supplementary Files

Supplementary Data 1: Sample metadata

Sheet 1: Clinical metadata

Clinical parameters per sample: Sample_ID; Patient_ID; Time point and patient matched IDs for EDTA_blood, Streck_tubes, Tissue_01, Tissue_02, Ascites; Samples_sent_to_TuPro = total number of samples; TuPro_T1-3 = sampling site of the priority 1-3 samples. Site corresponds to the non-empty sample IDs in Tissue_01, Tissue_02, and Ascites columns; primary_neo_relapse = treatment status at the time of sampling; Age_range; Origin; Grade; FIGO_01; Residual_disease; Last_treatment_before_Tupro_chemotherapy Last_treatment_before_Tupro_secondary_drugs; CA125 in kU/l; MSStatus_patho = microsatellite stability determined by routine pathology; ER_Patho; ER_Patho_grouped; PR_Patho; PR_Patho_grouped; PFS_0_1 = Progression free survival event, i.e. whether the patient progressed during the TuPro follow-up time; PFS_Time_to_1st_relapse_mths = time to relapse in months; DSS_0_1 = Disease-specific survival event, i.e. whether a disease-specific death occurred during the Tupro follow-up time; DSS_Time_to_death_mths; Last_FU = time to last follow-up in months; Response_first_line_chemotherapy; Platinum_status at initial diagnosis; Treatment_after_Tupro_sampling_chemotherapy; Treatment_after_Tupro_sampling_secondary_drugs

Sheet 2: Sample groupings provided by Tupro technologies

- scDNA_OncCas: Whether the oncology cassette (3q26) was found to be amplified by scDNA-seq
- BulkT_jnci_group: Grouping of samples into the molecular subtypes mesenchymal, differentiated, immunoreactive and proliferative based on bulk RNA-seq (see Figure 5A)
- PCY_Overall_Response: Grouping of samples based on overall ex vivo response by PCY (see Figure 6A).
- scDNA_has_WGD: Whether a sample was characterized by whole genome duplication (WGD) by scDNA-seq.
- heterogeneity_group_intra: Grouping of samples based on intra-tumor heterogeneity (see Figure 7)
- heterogeneity_group_inter: Grouping of samples based on inter-tumor heterogeneity (see Figure 7)

Supplementary Data 2: FoundationOne-based genomic alterations

Sheet 1: Genetic alterations measured by FoundationOne CDx. Alterations are encoded by type (SV: Short variant, AMP: amplification, DEL: deletion, RA: rearrangement). For each SV, the affected amino acid change is indicated.

Sheet 2: Composite biomarkers (loss of heterozygosity, microsatellite stability and tumor mutational burden) derived from FoundationOne CDx.

Supplementary Data 3: Bulk RNA sequencing

Sheet 1: Raw gene counts per sample.

Sheet 2: Normalized, batch-corrected and variance-stabilized gene counts per sample.

Sheet 3: Normalized, batch- and tumor content corrected and variance-stabilized gene counts per sample.
Sheet 4: Pathway activation scores per sample.
Sheet 5: Tumor content corrected pathway activation scores per sample.

Supplementary Data 4: bulk proteomics (proteotyping)
Sheet 1: Protein abundance per sample. Values represent batch corrected raw intensities (Čuklina et al. 2021).
Sheet 2: Pathway scores from protein abundances, obtained from GSVA.
Sheet 3: Tumor-content corrected pathway activation scores.

Supplementary data 5: Mutational profiles by ctDNA-seq.

Supplementary Data 6: scRNA-seq sample-level data
Sheet 1: Bulkified gene expression in HGSOC cells.
Sheet 2: Pathway activation scores derived from bulkified gene expression in HGSOC cells.

Supplementary Data 7: Clone frequencies and associated copy number profiles per sample from scDNA-seq
This table summarizes the different cancer cell clones detected per sample by scDNA-seq. The column "clone_fraction" indicates the frequency of each clone among all detected clones in the tumor, all subsequent columns indicate the copy number profile for selected disease-relevant genes (0 = no copy number change, negative values = copy number loss, positive values = copy number gain). Clones without alterations in the selected genes are not included.

Supplementary Data 8: CyTOF sample-level data
Sheet 1: Marker expression per sample. Values represent normalized aggregate intensities across all HGSOC cells per sample.
Sheet 2: Sample composition

Supplementary Data 9: IMC sample-level data
Sheet 1: Aggregated marker expression per cell type and sample.
Sheet 2: Cell composition per sample.

Supplementary Data 10: Pharmacoscopy (PCY)
Sheet 1: PCY scores per drug and sample. The PCY score represents 1 - DMSO-normalized cancer cell fraction, averaged across all replicates and drug concentrations. Scores were further scaled to the tumor content (see methods for detail) to correct for systematic biases in the effect size.
Sheet 2: Fraction and number of cells per cell type and well. Values are corrected for systematic effects (e.g. row / column gradients) across the assay plate.
Sheet 3: Drug annotation
Sheet 4: Experiment details per sample.

Supplementary Data 11: 4i DRP
Rows correspond to samples, columns to combinations of a drug treatment and a marker. Values represent average cellular fluorescence intensity values, z-scored to DMSO control.

Supplementary Data 12: Clinical utility
Clinical metadata and MTB variables used for clinical utility analysis. The table only includes patients that were discussed at the MTB. If a patient ID occurs more than once (e.g. **TP-G2-USB-004**), this indicates that longitudinal data were collected for this patients at two different visits.

Supplementary Data 13: List of curated gene sets used in this study.

Supplementary Data 14: List of antibodies used in this study

Supplementary Data 15: Variables used in integrative analysis.
